# Novel sugar-conjugated Knoevenagel condensate curcumin derivatives as promising bioactive hybrids with antimalarial potential

**DOI:** 10.1039/d5ra09343k

**Published:** 2026-05-12

**Authors:** Siti Nur Hidayah Jamil, Natsuhisa Oka, Amatul Hamizah Ali, Yan Hong Ng, Nur Fatin Najihah Marzuki, Shevin Rizal Feroz, Su Datt Lam, Fauze Mahmud, Yusmazura Zakaria, Jalifah Latip

**Affiliations:** a International Joint Department of Materials Science and Engineering between National University of Malaysia and Gifu University, Graduate School of Engineering, Gifu University 1-1 Yanagido Gifu 501-1193 Japan sitinh.jamil@gmail.com oka.natsuhisa.f9@f.gifu-u.ac.jp; b Department of Chemical Sciences, Faculty of Science and Technology, Universiti Kebangsaan Malaysia UKM Bangi 43600 Selangor Malaysia jalifah@ukm.edu.my; c Department of Chemistry and Biomolecular Science, Faculty of Engineering, Gifu University 1-1 Yanagido Gifu 501-1193 Japan; d Center for One Medicine Innovative Translational Research (COMIT), Institute for Advanced Study, Gifu University 1-1 Yanagido Gifu 501-1193 Japan; e Institute for Glyco-core Research (iGCORE), Gifu University Gifu 501-1193 Japan; f Department of Biological Sciences and Biotechnology, Faculty of Science and Technology, Universiti Kebangsaan Malaysia UKM Bangi 43600 Selangor Malaysia; g Biomedicine Programme, School of Health Sciences, Universiti Sains Malaysia Health Campus Kota Bharu 16150 Kelantan Malaysia; h Structural Biology and Protein Engineering Research Group, Universiti Kebangsaan Malaysia Bangi 43600 Selangor Malaysia; i Department of Applied Physics, Faculty of Science and Technology, Universiti Kebangsaan Malaysia UKM Bangi 43600 Selangor Malaysia; j Center for Global Health Research (CGHR), Saveetha Medical College, Saveetha Institute of Medical and Technical Sciences (SIMATS), Saveetha University Chennai 602 105 Tamil Nadu India; k Faculty of Science and Technology, Universiti Malaysia Sabah Jalan UMS Kota Kinabalu 88400 Sabah Malaysia; l BioAgriTech Research (BioATR) Group, Faculty of Science and Technology, Universiti Malaysia Sabah Jalan UMS Kota Kinabalu 88400 Sabah Malaysia; m Smart Material and Sustainable Product Innovation (SMatSPIn) Research, Universiti Kebangsaan Malaysia UKM Bangi 43600 Selangor Malaysia; n Department of Chemistry, Faculty of Mathematics and Natural Sciences, State University of Malang (Universitas Negeri Malang) Jl. Semarang No. 5 Malang 65145 Indonesia

## Abstract

Malaria research is facing a concerning challenge due to the continuously increasing annual reported number of cases and deaths, along with the *Plasmodium* parasite's resistance against current first-line treatments, which emphasise the need for novel effective antimalarials to combat this resistance. While curcumin, a polyphenolic compound from *Curcuma longa*, has previously demonstrated *in vivo* efficacy in mediating malaria infections through glycogen synthase kinase-3 beta (GSK-3β) inhibition, the potential was suboptimal due to its bioavailability constraints. Building on this, novel sugar-conjugated Knoevenagel condensate curcumin derivatives were successfully synthesised, through *O*-glycosylation with galactose, glucose, and mannose, and evaluated for their antimalarial potential. Assessments revealed the influence of sugar moieties on bioactivity. Generally, sugar-conjugated compounds, which demonstrated enhanced aqueous solubility, showed enhancements in absorption, distribution, metabolism, excretion and toxicity (ADMET) profiles and density functional theory (DFT) results, along with better cytotoxicity profiles and favourable haemin (HMN) binding. Notably, the acetylated glucosyl derivative 2a-Glc exhibited significantly strong GSK-3β inhibition (−10.48 kcal mol^−1^) and evidently the most potent antiplasmodial compound with an EC_50_ of 1.53 ± 0.10 µM (3D7). Overall, the study demonstrated bioactivity improvements attributable to curcumin's structural derivatisation, with the acetylated glycoside 2a-Glc showing the greatest antimalarial potential based on *in silico* profiles, *Plasmodium falciparum* lactate dehydrogenase (pLDH) assays, HMN binding, and GSK-3β inhibition. This strategy highlighted refined synthesis strategies, *in silico* modelling, and biological evaluations in drug discovery, offering valuable insights into the benefits of exploring curcumin derivatives and glycosylation for developing promising bioactive compounds with antimalarial efficacy.

## Abstract 2

Malaria research is facing a concerning challenge due to the continuously increasing number of cases and deaths, along with the *Plasmodium* parasite's resistance against first-line treatments, emphasising the need for novel effective antimalarials.

## Graphical abstract

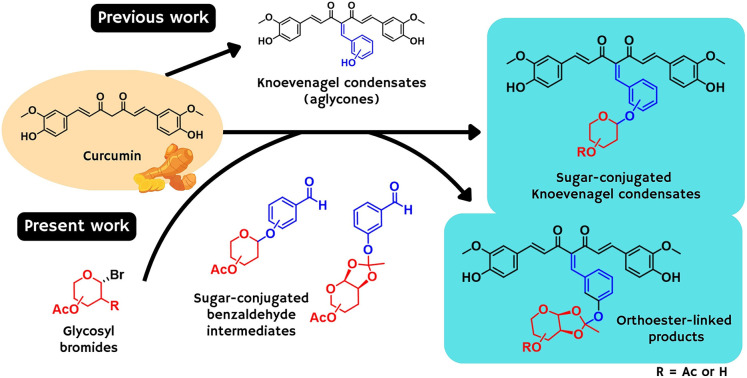

## Introduction

The global infection rate of malaria was estimated to have reached 263 million in 2023, covering 83 malaria-endemic countries, with a fatality rate of 13.7 per 100 000 population and an estimated 597 000 deaths.^1^ First identified in 1880, malaria is caused by *Plasmodium* parasites, mainly *P. falciparum* and *P. vivax*, infecting humans. Without timely and efficient intervention, the infection may progress to serious complications leading to cerebral and severe malaria, including, but not limited to, kidney failure, seizures, cognitive disorientation, coma, and ultimately, death.

Rapid emergence and spread of *Plasmodium* parasite resistance compromises the effectiveness of the present first-line medications for treating malaria patients, including the widely prescribed artemisinin, chloroquine (CQ) and artemisinin-based combination therapies (ACTs).^2–4^ Therefore, present works on antimalarial research are diverging towards finding alternative medicine in an attempt to take action before ACTs start to fail.^5–9^

The search and development of novel potent antimalarial compounds has currently revolved around hybrid compounds and bioactive natural products, which are anticipated to overcome the emergence of resistance against present medications. In research, glycosides are currently being extensively explored as promising bioactive compounds against various therapeutic conditions, including cancer,^10,11^ diabetes, microbial and bacterial diseases,^12–14^ viral and fungal infections,^15,16^ inflammation,^17,18^ as well as malaria.^19,20^ Recently, researchers have established the promising antimalarial potential of glycoside derivatives from dihydroartemisinin (DHA) and hydroxychloroquine (HCQ) derivatives against *Plasmodium falciparum* strains.^20–23^

Therefore, our work explored the sugar-conjugated derivatives from curcumin by conjugating several glycosyl donors to hydroxy-containing Knoevenagel condensate curcumin derivatives through *O*-glycosylation. As a naturally sourced compound, curcumin's accessibility potentially lowers development costs, attracting interest and investment from both research organisations and commercial entities, and fostering prospects for future antimalarial drug development. Its source availability could drive more comprehensive and cost-efficient endeavours in combating malaria through drug innovations. This polyphenolic compound from *Curcuma longa*, which is abundantly available in Asian countries, has been proven to potentially inhibit the plasmodial infection.

However, the therapeutic effects are generally unexceptional, showing only moderate efficacy in comparison to the present antimalarials. This is potentially due to its constraints within the bioavailability, which includes low solubility, rapid metabolism and excretion, which constrain curcumin's clinical application and recognition as an effective potential drug. Previous works demonstrated that derivatisation of curcumin through structural modification and metal-curcumin coordination improved its stability, bioavailability and delivery.^9,24–26^ The application of coordination chemistry by chelating curcumin to metal ion, including lanthanide, was shown to facilitate intersystem crossing, hence improving its delivery to the target cells, whereby the active curcumin could be released when the complex was photoactivated during photodynamic therapy (PDT) treatment.^27^

In this work, glycosylation is anticipated to overcome curcumin's limitation by improving curcumin's absorption and distribution for enhanced bioavailability, biological efficacy and cytotoxicity potential. These enhancements are attributable to the higher aqueous solubility and metabolic stability imparted by the presence of hydrophilic sugar moieties.^28–30^ Additionally, the ability of glycosides to potentially mimic glucose and exploit the carbohydrate transport mechanisms could not only facilitate controlled drug release and allow specifically targeted delivery,^31,32^ but also be preferentially taken up into the parasite and eventually interrupt the parasite's metabolic pathways.^6,33–35^ These highlight the significance of investigating the benefits of glycosides for discovering potential antimalarial agents. Herein we report the synthesis, antiplasmodial activity against pLDH 3D7 and K1 assays, *in silico* molecular docking to immunomodulatory GSK-3β protein, DFT calculations and ADMET assessments, and ITC HMN binding potential of novel glycosylated derivatives of hydroxybenzylidene curcumin.

## Results and discussion

### Synthesis of curcumin glycoside derivatives by Knoevenagel condensation

**Scheme 1:**
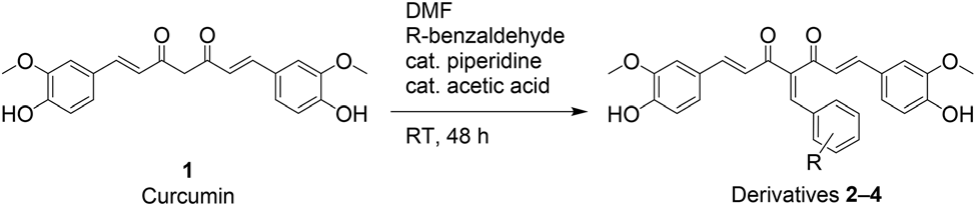
Synthesis of Knoevenagel condensates 2–4.

Glycosylation involves the process of conjugating a glycosyl donor at its anomeric position to a potentially bioactive aglycone. Previously, we have synthesised Knoevenagel condensates (Scheme 1) and established their promisingly enhanced antimalarial potential, relative to curcumin 1.^26^ However, the physicochemical properties, including the solubility of these compounds, remained suboptimal, which limits their bioavailability. Therefore, we selectively further derivatised Knoevenagel condensate curcumin derivatives 2, 3 and 4 with the presence of an additional hydroxy group through *O*-glycosylation with galactosyl, glucosyl and mannosyl bromides.

**Scheme 2:**
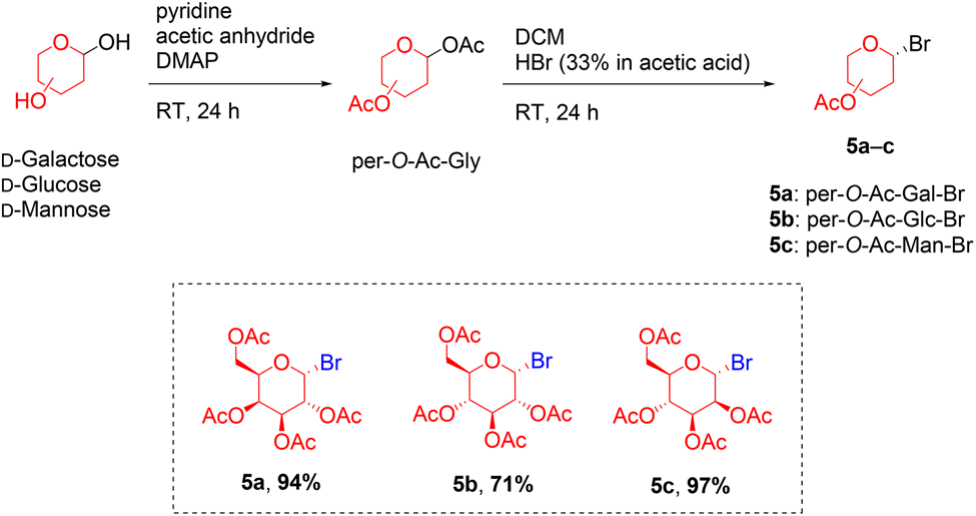
Synthesis of glycosyl donors; per-*O*-acetylated galactosyl bromide (5a), per-*O*-acetylated glucosyl bromide (5b) and per-*O*-acetylated mannosyl bromide (5c).

Glycosyl bromides (5a–c) were readily synthesised, according to the literature,^36,37^ from d-galactose, d-glucose and d-mannose to obtain the desired compounds in two synthetic steps; acetylation by acetic anhydride catalysed by DMAP, followed by treatment of HBr (33% in acetic acid), with 71–97% overall yield (Scheme 2).

**Scheme 3:**
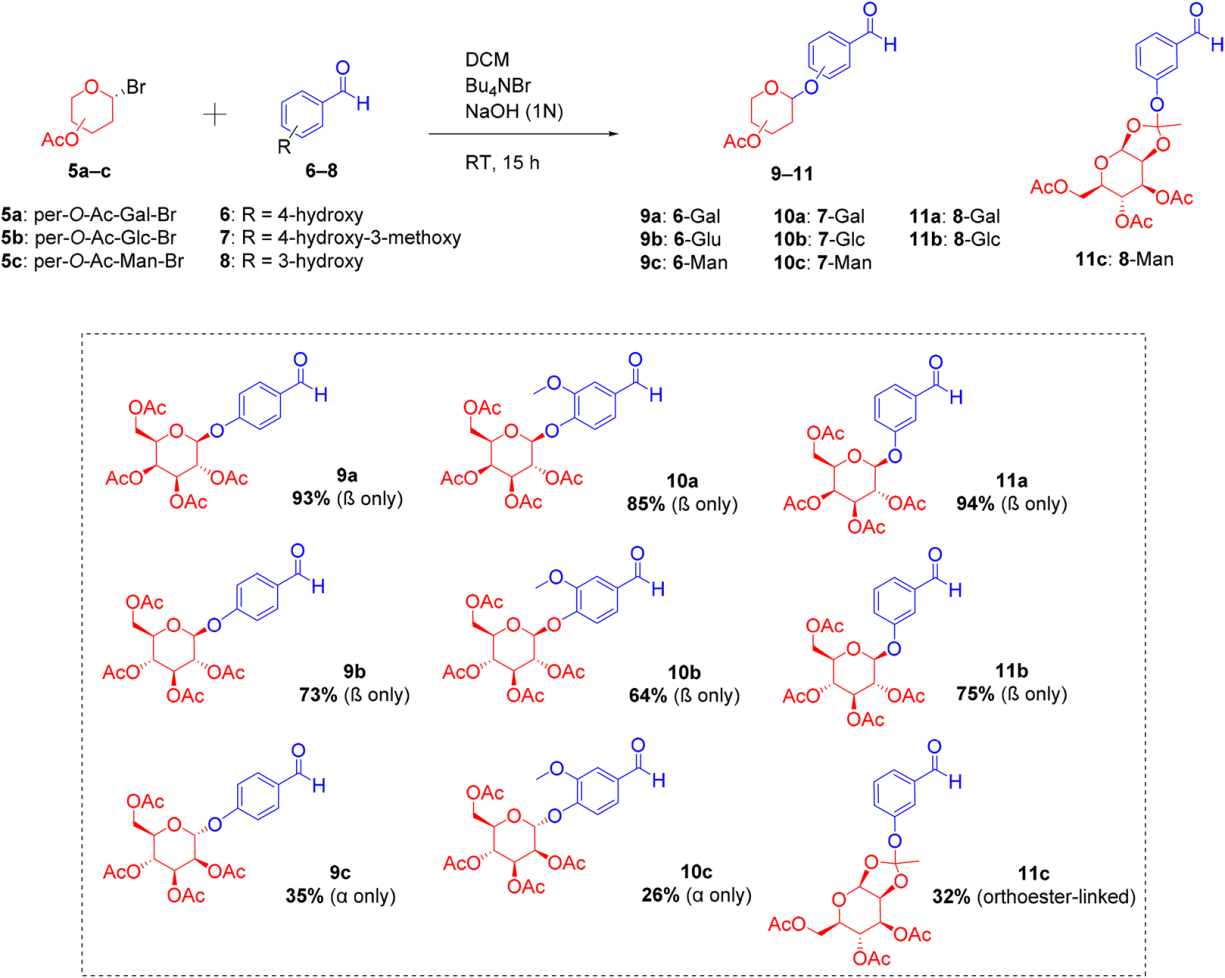
Synthesis of sugar-conjugated aldehyde intermediates; glycosylated 4-hydroxybenzaldehyde (9a–c), glycosylated vanillin (10a–c) and glycosylated 3-hydroxybenzaldehyde (11a–c).

Next, the glycosylation of the appropriate hydroxybenzaldehydes (6–8) was carried out through standard carbohydrate chemistry, using the acetyl-protected glycosyl bromides 5a–c (Scheme 3 and Table 1).^38^ The reaction adopted milder conditions, whereby, in literature, the frequent use of more expensive and toxic mercury salts- or silver salts-promoted Helferich method was not suitable for *O*-glycosylating acetoxybenzyl alcohols.^39^ Following this, the reactions for glycosylating hydroxybenzaldehydes 6–8 were performed in biphasic conditions with the presence of DCM and 1 N NaOH (aq.) and catalysed by Bu_4_NBr (15 hours).^38^ The presence of NaOH facilitated the reaction, deprotonating the phenolic hydroxy of the benzaldehyde, hence generating the essential highly nucleophilic phenoxide ion. The Bu_4_N^+^ will then facilitate the transport of phenoxide ion across the phase, allowing glycosylation to proceed in the organic phase. All desired compounds were successfully obtained, with the yields of mannose-conjugated intermediates (26–35%) being, however, lower than those of galactose (85–94%) and glucose (64–75%).

**Table 1:** Optimisation of the glycosylation reaction for *R*-benzaldehyde^a^

Entry	Glycosyl bromide : *R*-benzaldehyde molar ratio	Concentration, M	Time, h	Yield, %
1	1.00 : 1.10	0.10	3	15
2	1.00 : 1.50	0.10	3	18
3	1.00 : 2.00	0.40	3	44
4	1.00 : 2.00	0.80	15	85

a The optimised condition was based on the reaction between galactosyl bromide 5a and vanillin 7.

**Scheme 4:**
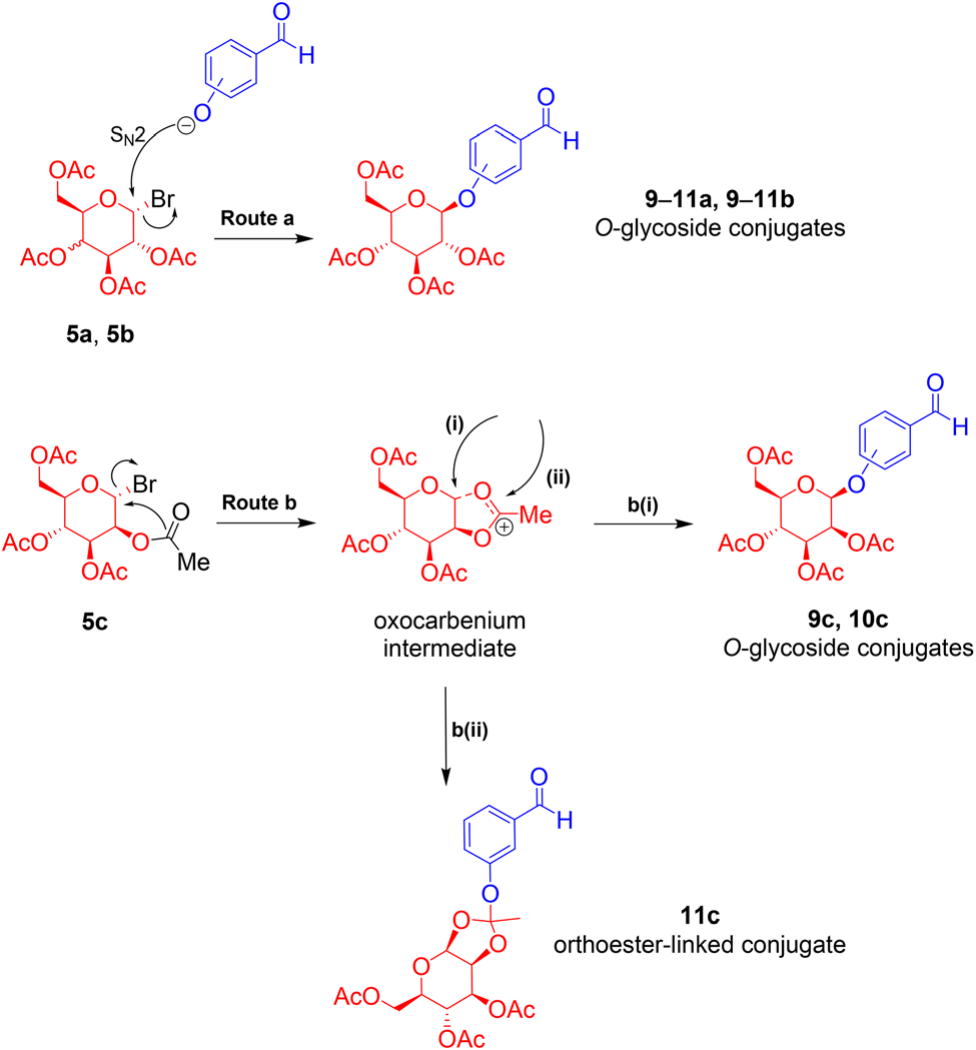
Reaction mechanisms showing the formation of the glycosidic bond and orthoester linker; (route a) *O*-glycosides of galactose and glucose conjugates, and (route b) *O*-glycosides and orthoester-linked mannose conjugates.

The reaction between 3-hydroxybenzaldehyde 8 and mannose derivative 5c, interestingly, yielded the orthoester-linked conjugates. This is due to the 1,2-*trans* (5c) relationships between the C-2 acetyl and anomeric position, which affected the stereoselectivity and reactivity of the glycosylation process (Scheme 4). Glycosylation reactions involving galactose 5a and glucose 5b proceeded through route a *via* S_N_2, with Br^−^ being a good leaving group. Whereas, the reaction with mannose 5c may preferentially happen *via* route b, *via* the generation of oxocarbenium intermediate, and proceed through b(i) or b(ii) pathway. The difference amongst these reactions was likely due to the differences in nucleophilicity of the phenoxides. While the less nucleophilic phenoxide ion from *para*-hydroxybenzaldehydes 6 and 7 attacked at position (i), yielding *O*-glycosides 9c and 10c, the more nucleophilic ion from *meta*-hydroxybenzaldehyde 8 preferentially attacked at position (ii) and readily transformed into stable orthoester-linked 11c.^40,41^

**Scheme 5:**
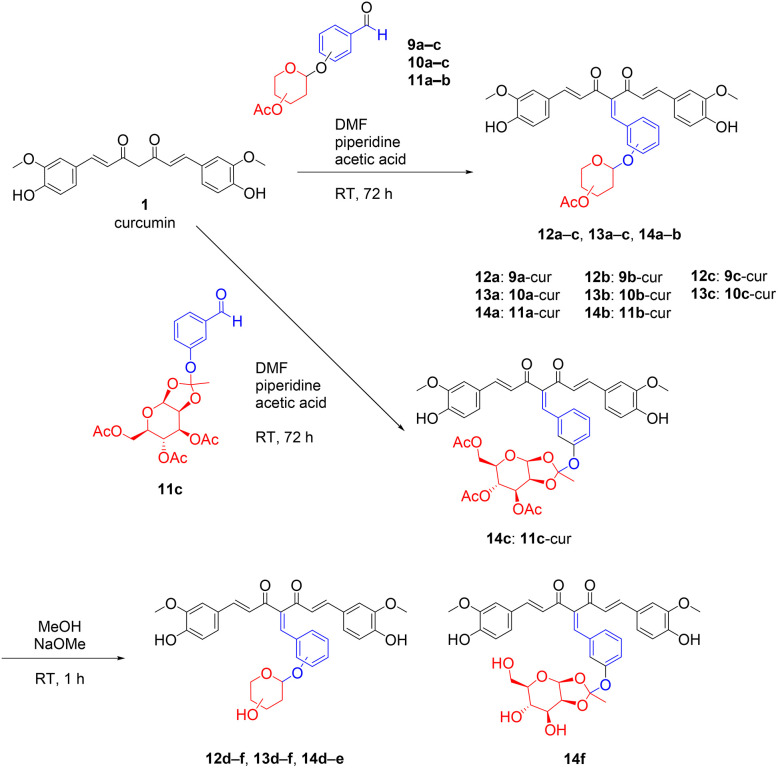
Synthesis of acetyl-protected (2a-Gly, 3a-Gly and 4a-Gly), and the deacetylated curcumin glycoside derivatives (3b-Gly, 4b-Gly and 6b-Gly).

Further, the conjugation of curcumin 1 with the sugar-conjugated benzaldehyde intermediates was carried out through Knoevenagel condensation, using optimised conditions (Scheme 5 and Table 2). Good and sufficient yields were obtained for the desired products of acetyl-protected glycoside derivatives 2a-Gly, 3a-Gly and 4a-Gly (23–79%) (Scheme 5).

**Table 2:** Optimisation of the Knoevenagel condensation reaction conditions for conjugating curcumin to sugar-conjugated benzaldehydes^a^

Entry	Curcumin : sugar-conjugated benzaldehyde molar ratio	Catalyst molar equivalent (piperidine : acetic acid)	Time, h	Yield, %
1	1.00 : 1.50	1.00 : 1.30	48	28
2	1.00 : 2.00	1.00 : 1.30	48	27
3	1.00 : 2.00	2.00 : 2.60	48	50
4	1.00 : 2.00	2.00 : 2.60	72	66

a The optimised condition was based on the reaction between curcumin 1 and glucose-conjugated vanillin 10b.

**Fig. 1:**
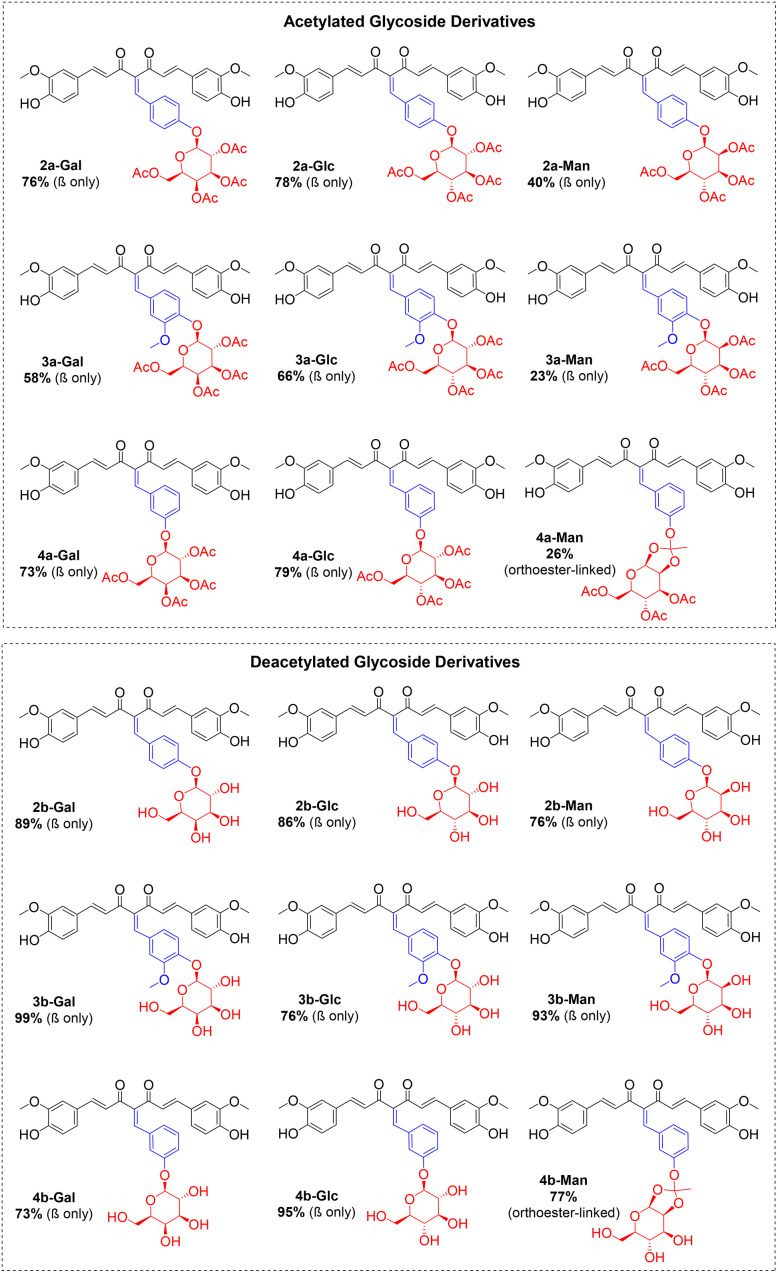
Structures of the synthesised acetylated and deacetylated curcumin glycoside derivatives.

In the final step, the acetyl-protected glycoside derivatives (2a-Gly, 3a-Gly and 4a-Gly) were deacetylated using NaOMe in MeOH (Scheme 5). The desired deacetylated products (3b-Gly, 4b-Gly and 6b-Gly) were obtained in high yields at ≥73% (Fig. 1).

The structures of the orthoester-linked compounds 11c, 4a-Man and 4b-Man were characterised and validated based on the presence of a more shielded singlet peak at 1.50–1.70 ppm on ^1^H NMR spectra, which represents the three protons from the methyl group of the orthoester linkage. In addition, based on the ^1^H NMR, the regions for protons from the sugar moiety were only observed at 3.00–5.50 ppm, representing the equatorial configuration for the β-isomer, and no peaks were detected at 5.50–6.50 ppm for the α-isomer product. Primarily, the use of glycosyl bromides as the donor for glycosylation will enforce 1,2-*trans* glycosidic linkages, hence, producing stereocontrolled *O*-glycosylated compounds.^39^ The neighbouring C2-*O*-acetyl group participation sterically shielded the axial side for α-isomer formation, and, additionally, the bulky benzylidene curcumin scaffold also sterically disfavours the α-product.^42^

### *In vitro* pLDH antiplasmodial activity and cytotoxicity

The *in vitro* pLDH assessment of the synthesised compounds is summarised in Table 3. The acetylated derivatives of the galactose and glucose conjugates generally showed better antiplasmodial activities against both 3D7 and K1 strains compared to the deacetylated compounds. Generally, among the galactoside derivatives, only 3a-Gal, 3b-Gal and 4a-Gal showed better activity against the 3D7 strain, while all compounds exhibited better activity against K1, compared to curcumin 1. Notably, the deacetylated galactoside 4a-Gal exhibited the most potent activities against 3D7 (EC_50_ = 1.71 ± 0.23 µM) and K1 (EC_50_ = 5.76 ± 0.25 µM). These values are relatively comparable to the bioactivity of 4 against 3D7 and better against K1 (EC_50_ = 1.15 ± 0.26 µM (3D7) and 11.01 ± 2.67 µM (K1)).

**Table 3:** Anti-plasmodial activities of the glycoside derivatives against *P*. *falciparum* 3D7 (CQ-sensitive strain), *P*. *falciparum* K1 (multi-drug-resistant strain), and cytotoxic evaluation against WRL-68 human cells^a^

Compound	EC_50_, µM		WRL-68 CC_50_ ± SD, µM	Selectivity index (SI)		Resistance index (RI)
	3D7	K1		3D7	K1	
CQ	0.008 ± 0.003	0.53 ± 0.12	98.82 ± 0.2	12 352.50	186.45	66.25
1	8.32 ± 2.62	30.66 ± 5.44	>99	11.90	3.23	3.69
2	4.35 ± 2.43	13.33 ± 7.23	3.34 ± 1.09	0.77	0.25	3.06
3	1.79 ± 0.35	18.84 ± 0.78	0.31 ± 0.11	0.17	0.02	10.53
4	1.15 ± 0.26	11.01 ± 2.67	0.19 ± 0.07	0.17	0.02	9.57

**Galactoside derivatives**						
2a-Gal	11.14 ± 1.04	26.37 ± 0.70	1.19 ± 0.07	0.11	0.05	2.37
3a-Gal	4.04 ± 0.60	13.51 ± 0.68	1.15 ± 0.02	0.28	0.09	3.34
4a-Gal	1.71 ± 0.23	5.76 ± 0.25	0.88 ± 0.08	0.51	0.15	3.37
2b-Gal	12.07 ± 1.08	26.38 ± 1.69	19.66 ± 1.34	1.63	0.75	2.19
3b-Gal	7.97 ± 0.87	16.98 ± 1.23	36.99 ± 1.82	4.64	2.18	2.13
4b-Gal	8.71 ± 0.90	10.28 ± 1.02	32.38 ± 0.81	3.72	3.15	1.18

**Glucoside derivatives**						
2a-Glc	1.53 ± 0.10	13.41 ± 0.51	1.27 ± 0.05	0.83	0.09	8.76
3a-Glc	7.72 ± 1.61	13.49 ± 0.78	0.09 ± 0.01	0.01	0.01	1.75
4a-Glc	14.2 ± 1.15	35.67 ± 1.50	0.54 ± 0.01	0.04	0.02	2.51
2b-Glc	7.60 ± 0.23	23.04 ± 1.36	43.81 ± 1.89	5.76	1.90	3.03
3b-Glc	15.74 ± 1.20	73.91 ± 1.03	34.18 ± 1.98	2.17	0.46	4.70
4b-Glc	4.65 ± 0.11	27.43 ± 1.43	21.63 ± 0.09	4.65	0.79	5.90

**Mannoside derivatives**						
2a-Man	12.12 ± 1.08	15.41 ± 0.11	0.37 ± 0.03	0.03	0.02	1.27
3a-Man	11.94 ± 0.31	26.78 ± 1.42	0.51 ± 0.07	0.04	0.02	2.24
4a-Man	13.38 ± 0.30	33.88 ± 1.53	0.51 ± 0.01	0.04	0.02	2.53
2b-Man	13.41 ± 0.43	42.37 ± 1.62	2.98 ± 0.12	0.22	0.07	3.16
3b-Man	9.11 ± 0.95	13.39 ± 0.34	7.92 ± 0.45	0.87	0.59	1.47
4b-Man	5.26 ± 0.71	52.16 ± 1.71	1.12 ± 0.11	0.21	0.02	9.92

a The data are presented as the average from triplicate measurements.

Further, with the exception of derivatives 4a-Glc and 3b-Glc, all glucoside derivatives generally showed better antiplasmodial activities against both strains, compared to curcumin. Particularly, derivative 2a-Glc exhibited enhanced EC_50_ against 3D7 strain (EC_50_ = 1.53 ± 0.10 µM) and comparable against K1 (EC_50_ = 13.41 ± 0.51 µM), and only derivative 3a-Glc was more effective against K1 (EC_50_ = 13.49 ± 0.78 µM), compared to the aglycones 2 and 3. In contrast with the galactose and glucose derivatives, the deacetylated series of mannose-conjugated compounds, except 3b-Man, generally possessed better activities compared to the acetylated compounds. The orthoester-linked derivative 4b-Man also showed enhanced activity against 3D7 (EC_50_ = 5.26 ± 0.71 µM) compared to curcumin, proposing the influence of linkage type for bioactivity. Furthermore, all mannose derivatives, except 3b-Man (EC_50_ = 13.39 ± 0.34 µM), were, however, less potent against the K1 strain compared to the parent aglycones, while only 2a-Man and 3a-Man demonstrated better EC_50_ values than curcumin (15.41 ± 0.11 µM and 26.78 ± 1.42 µM, respectively).

With regard to the safety profiles of the derivatives, the cytotoxicity and SI analysis revealed that most compounds exhibited substantially reduced cytotoxic potential compared to the aglycones, with, in general, the deacetylated derivatives showed relatively higher CC_50_ values than the acetylated compounds, while mannose derivatives particularly exhibited the lowest CC_50_ values, suggesting that these compounds possessed the highest potential for cytotoxicity among all sugar types. The SI, which represents the balance between efficacy and cytotoxicity for therapeutic potential, revealed that all derivatives possessed higher SI values against 3D7, but lower against K1, hence stronger selectivity for CQ-sensitive over the resistance strain. Meanwhile, the deacetylated galactoside 4b-Gal specifically showed comparable values against both strains (SI = 3.72 (3D7) and 3.15 (K1)), suggesting its potential to overcome resistance, better than other glycosides. Additionally, only the deacetylated galactoside and glucoside derivatives displayed higher SI values (SI > 1), suggesting that they have better selectivity and are more toxic towards the parasite than normal cells.

Overall, the *in vitro* antiplasmodial efficacy revealed that glycoside derivatives are generally more active against the CQ-sensitive 3D7 over the CQ-resistant K1 strain, with the acetylated series demonstrating more potent EC_50_ values compared to the deacetylated. While most derivatives relatively remained more effective as antiplasmodial agents than curcumin, the acetylated glycosides 2a-Glc and 4a-Gal are the most potent. The overall pLDH results emphasised the significant influence of the types of conjugated sugar on bioactivity. Further cytotoxicity assessment unveiled that most sugar-conjugated derivatives possessed significantly better safety, hence validating the benefits of sugar moiety on toxicity.^43^ In addition, higher SI presented for these glycosides compared to their parent aglycones suggests that conjugating sugars favours enhanced therapeutic potential against the *Plasmodium* parasites.

### Binding interactions to GSK-3β from molecular docking

The molecular docking assessment of the glycoside derivatives further demonstrated the benefits attributable to glycosylation for GSK-3β inhibition potential. GSK-3β is recognised as a novel target for antimalarial drugs due to its involvement in the dysregulated PI3K/Akt signalling triggered by the invasion of *Plasmodium* parasites, whereby inhibiting GSK-3β has been proven to mediate antimalarial effects in humans.^44^ The function of GSK-3β is highly dependent on the hinge region containing conserved residues such as Asp133 and Val135 that make up an essential part of the ATP binding pocket.^45^ This facilitates the catalytic activity of GSK-3β, leading to the regulation of pro- and anti-inflammatory cytokines through NF-κB activation.

The potential of curcumin in inhibiting GSK-3β was preliminarily established through computational simulations,^46^ and recently proven *in vivo* by Ali *et al.*^44,47^ Further, our previous work has also presented the potential of the aglycone Knoevenagel condensates in inhibiting the protein based on enhanced binding profiles (binding energies of −9.07 to −9.42 kcal mol^−1^) compared to curcumin (−7.44 kcal mol^−1^).^26^ In this work, the incorporation of sugar moiety introduced scaffolds with significant additional H-bond donors and acceptors, which increased polarity and enhanced the solvation and orientation within the binding cavity, hence the capacity for stronger GSK-3β inhibition.

**Fig. 2:**
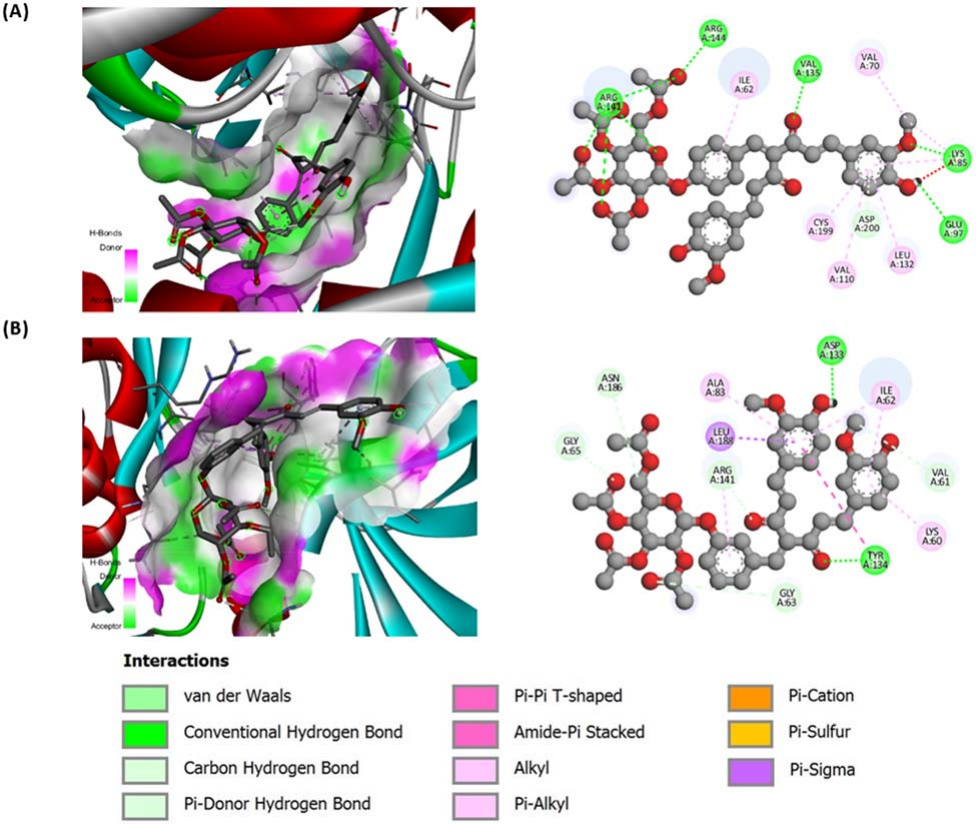
Visualisation of the interaction of (A) 2a-Glc and (B) 4a-Gal within the binding site of GSK-3β.

The potential of acetylated glucoside 2a-Glc to inhibit GSK-3β outperformed the aglycone and curcumin based on the stronger binding energies (−10.48 kcal) and smaller *K*_i_ (0.02 µM) (Table 4). The complex formed was primarily stabilised through conventional hydrogen bonds (Lys85, Glu97, Val135, Arg141, and Arg144), carbon hydrogen bond (Asp200), and hydrophobic interactions (Ile62, Val70, Lys85, Val110, Leu132 and Cys199) (Fig. 2). While the interaction between acetylated galactoside 4a-Gal and GSK-3β also involved conventional hydrogen bonds (Asp133 and Tyr134), carbon hydrogen bonds (Val61, Gly63, Gly65, Arg141 and Asn186) and hydrophobic interactions (Lys60, Ile62, Ala83, Tyr134, Arg141 and Leu188), with binding energy of −8.12 kcal mol^−1^ and *K*_i_ of 1.13 µM, however, less residues participated for conventional hydrogen bonding.

**Table 4:** Curcumin glycoside derivatives categorised according to the type of conjugated sugar and the affinity towards GSK-3β

Compound	Binding Energy, kcal mol^−1^	Inhibition constant (*K*_i_), µM	Type of interaction	Amino acid residues involved in binding
1	−7.44	1.02	Hydrogen bonds	Asp133, Tyr134, Arg141, Asn186, Cys199
			Hydrophobic interactions	Ile62, Val70, Ala83, Leu188, Cys199
2	−9.42	1.04	Hydrogen bonds	Ile62, Asp133, Tyr134, Pro136, Arg144, Cys199
			Hydrophobic interactions	Ile62, Val70, Ala83, Val110, Leu132, Arg141, Leu188, Cys199
3	−9.07	1.01	Hydrogen bonds	Val61, Arg141, Asp133
			Hydrophobic interactions	Ile62, Gly63, Val70, Ala83, Leu132, Leu188, Cys199
4	−9.08	0.28	Hydrogen bonds	Ile62, Lys85, Val135, Arg141, Cys199, Asp200
			Hydrophobic interactions	Ile62, Val70, Ala83, Lys85, Val110, Leu132, Tyr134, Cys199
2a-Glc	−10.48	0.02	Hydrogen bonds	Lys85, Glu97, Val135, Arg141, Arg144, Aps200
			Hydrophobic interactions	Ile62, Val70, Lys85, Val110, Leu132, Cys199
2b-Glc	−9.75	0.07	Hydrogen bonds	Ile62, Lys85, Glu97, Tyr134, Val135, Pro136, Aps200
			Hydrophobic interactions	Ile62, Val70, Lys85, Met101, Val110, Leu132, Cys199
4a-Gal	−8.12	1.13	Hydrogen bonds	Val61, Gly63, Gly65, Asp133, Tyr134, Arg141, Asn186
			Hydrophobic interactions	Lys60, Ile62, Ala83, Tyr134, Arg141, Leu188
4b-Gal	−10.31	0.03	Hydrogen bonds	Val61, Ile62, Asn64, Lys85, Glu97, Tyr134, Val135, Aps200
			Hydrophobic interactions	Lys60, Ile62, Val70, Lys85, Val110, Leu132, Cys199

These observations showed that derivative 2a-Glc exhibited a better binding profile compared to 4a-Gal, presumably due to a greater instance of conventional hydrogen bonds. In addition, the docking profile also demonstrated that the deacetylated glucoside derivative 4b-Gal exhibited stronger GSK-3β inhibition with a binding energy of −10.31 kcal mol^−1^ and *K*_i_ of 0.03 µM, than the acetylated 4a-Gal. This was potentially due to the capacity for H-bonding from the preserved free hydroxy groups of 4a-Gal. In contrast, the deacetylated glucoside derivative 2b-Glc bound less favourably to GSK-3β compared to the acetylated 2a-Glc, with a binding energy of −9.75 kcal mol^−1^ and *K*_i_ of 0.07 µM. The acetyl groups of the acetylated derivative seemingly favour better interactions, likely through steric and orientation complementary with the residues within the binding site, hence stabilising the complex. Regardless of the types of glycoside derivatives, all assessed compounds possessed binding energies and *K*_i_ values stronger than curcumin. Throughout, this docking analysis underscores the potential sugar-type selectivity for GSK-3β inhibition, with the acetylated glucoside derivatives preferably bind more effectively than the galactoside.

### Physicochemical ADMET analysis

The physicochemical properties of the synthesised glycosides were also assessed based on the predicted ADMET profiles to understand their therapeutic potential at physiological conditions (Table 5). All assessed glycosides were predicted to maintain no PAINS alert, hence expected not to be associated with potential false positive results. Furthermore, the log *P* values showed that those glycoside derivatives were predicted to have lower lipophilicity than those aglycones. The predicted values were much lower, especially for the deacetylated derivatives 2b-Glc and 4b-Gal. The predicted log *S* were also higher (less negative) than the aglycones and curcumin, signifying that the glycoside derivatives are expected to have better aqueous solubility, hence improved dissolution and absorption into the bloodstream.

**Table 5:** Predicted ADMET profiles of curcumin 1, aglycone derivatives 2, 3 and 4, glycoside derivatives 2a-Glc, 2b-Glc, 4a-Gal and 4b-Gal

Properties	Parameters	ADMET scores							
		1	2	3	4	2a-Glc	2b-Glc	4a-Gal	4b-Gal
Absorption	MW, g mol^−1^	368.38	472.49	502.51	472.49	802.77	634.63	802.77	634.63
	H-bond acceptor	6	7	8	7	16	12	16	12
	H-bond donor	2	3	3	3	2	6	2	6
	log *P*, log(mol L^−1^)	3.03	4.05	4.16	4.02	3.98	2.06	4.03	2.06
	TPSA, Å^2^	93.06	113.29	122.52	113.29	216.72	192.44	216.72	192.44
	PAINS alert	0	0	0	0	0	0	0	0
	log *S*, log(mol L^−1^)	−4.45	−5.98	−6.08	−5.98	−3.50	−3.62	−3.46	−3.63
	Intestinal absorption, %	75.49	76.07	70.19	76.14	73.90	29.80	78.90	31.00
Distribution	BBB permeability, log(mol L^−1^)	−0.53	−1.02	−1.32	−1.14	−0.77	−0.63	−0.82	−0.67
	CNS permeability, log(mol L^−1^)	−2.96	−2.94	−3.10	−2.98	−3.42	−3.75	−3.39	−3.81
Metabolism	CYP2D6/CYP3A4 substrate	No/yes	No/yes	No/yes	No/yes	No/yes	No/yes	No/yes	No/yes
	CYP2D6/CYP3A4 inhibitor	No/yes	No/yes	No/yes	No/yes	No/no	No/no	No/no	No/no
	CYP1A2 inhibitor	No	No	No	No	No	No	No	No
	CYP2C19/CYP2C9 inhibitor	Yes/yes	Yes/yes	Yes/yes	Yes/yes	Yes/no	No/no	Yes/no	No/no
Excretion	Total clearance, log(mL min^−1^ kg^−1^)	0.07	0.01	0.01	0.02	0.36	0.40	0.53	0.36
	Renal OCT2 substrate	No	No	No	No	No	No	No	No
Toxicity	MTRD (human)	0.51	0.19	0.01	0.16	1.43	0.89	1.37	0.91
	Ames toxicity	No	No	No	No	No	Yes	Yes	Yes
	Hepatotoxicity	No	No	No	No	Yes	No	Yes	No
	Cytotoxicity	No	No	Yes	No	Yes	No	Yes	No

In addition, the glycoside derivatives were also experimentally tested for their solubility in solvents commonly used in organic synthesis, including chloroform (CHCl_3_), dichloromethane (DCM), ethanol (EtOH), methanol (MeOH), acetonitrile (MeCN), dimethylformamide (DMF) and dimethylsulfoxide (DMSO), which were classified based on the empirical standards described by the United States Pharmacopeia (USP) (Table 6).^48^ The designed and synthesised derivative compounds developed from curcumin through structural progression led to a build-up of solubility profile across solvents with varying polarity. From curcumin, which is highly soluble in aprotic solvents but poorly soluble in methanol and ethanol, the glycoside derivatives are more freely and readily soluble in polar protic solvents. This underscores the solubilising effect of glycosylation, where the addition of a sugar moiety enhances aqueous compatibility and is favourable for developing compounds with optimal solubility and permeability in physiological environments.

**Table 6:** Qualitative solubility profile of curcumin 1, the aglycone and glycoside derivative compounds in common organic solvents at 500 mg per mL concentration, under RT

Compound	Solubility in organic solvents						
	CHCl_3_	DCM	EtOH	MeOH	MeCN	DMF	DMSO
Curcumin 1	Slightly soluble	Poorly soluble	Poorly soluble	Poorly soluble	Very soluble	Very soluble	Very soluble
Aglycones 2–4	Freely soluble	Freely soluble	Poorly soluble	Poorly soluble	Very soluble	Very soluble	Very soluble
Acetylated glycosides	Freely soluble	Freely soluble	Slightly soluble	Slightly soluble	Very soluble	Very soluble	Very soluble
Deacetylated glycosides	Poorly soluble	Poorly soluble	Freely soluble	Very soluble	Slightly soluble	Very soluble	Very soluble

Despite this, the glycoside derivatives were predicted to possess diverging intestinal absorption. While most compounds were expected to have favourable values of above 30%, the deacetylated derivatives 2b-Glc and 4b-Gal showed much lower absorption rate (30% and 31%, respectively) than the acetylated 2a-Glc and 4a-Gal (74% and 79%, respectively). In addition, highly water-soluble glycosylated compounds are also often absorbed in the gastrointestinal tract more efficiently, hence ultimately enhancing their bioavailability and minimised toxicity.^43^ Thus, these glycoside derivatives were expected to be sufficiently absorbed into the systemic circulation for optimal therapeutic effect.

The values of log BB and log PS, which were regarded as the blood–brain-barrier (BBB) and central nervous system (CNS) permeabilities, appeared to be negative and very similar to those of the aglycones and curcumin. This classified that the glycoside derivatives remained unable to readily cross the BBB and penetrate the CNS, hence, reducing the risk of neurological side effects.

Further, the excretion profile of the glycoside derivatives was predicted that the total clearance values suggested that the glycoside compounds could potentially be excreted faster and have shorter *in vivo* availability than the aglycones and curcumin. Nevertheless, the potential of these glycosides as non-substrates of renal OCT2 suggests otherwise, that these compounds would slowly be taken up to the renal cells. Thus, these derivatives are also expected to be less rapidly excreted, leading to slower clearance and less likely to be accumulated in renal tubular cells. This will reduce the risk of nephrotoxicity while also being favourable for a longer effective duration of action and enhanced bioavailability compared to curcumin.

Lastly, the toxicity assessments showed that the predicted maximum tolerated dose (MTRD) of all conjugates was higher compared to the aglycones, with the values for acetylated derivatives (2a-Glc and 4a-Gal) being higher than those of the deacetylated (2b-Glc and 4b-Gal). This suggests that those with higher MTRD could potentially exert biological effects with lower risk for adverse effects if overdosed. In addition, glycoside 2a-Glc showed improved safety with no potential Ames toxicity (mutagenicity). However, it appeared to be potentially hepatotoxic and cytotoxic.

Overall, the designed glycoside derivatives are generally predicted to possess better physiochemical properties, hence bioavailability, relative to the aglycones and curcumin. Most derivatives were predicted to have enhanced ADMET properties, consistently across all parameters including lipophilicity, water solubility, BBB and CNS permeability, excretion profile and toxicity. Further experimental solubility analysis revealed the enhancement in aqueous solubility brought in through the addition of sugar moiety. Thus, this presented the significance of exploring the potential of sugar-conjugated derivatives of curcumin, with improved systemic absorption, solubility profiles, and formulation potential, as promising antimalarial agents.

### DFT geometry optimisation analysis

Analysis based on DFT calculations enables the understanding of the potential of compounds as drug candidates with optimal pharmacokinetics and bioavailability.^49^ The geometry and charge distribution of a molecule, comprising the molecular orbital (MO) descriptors including molecular structure energy *E*, highest occupied molecular orbitals (HOMO), lowest unoccupied molecular orbitals (LUMO), and dipole moment, are important factors affecting the biological activities of a compound.^50,51^ In addition, the reactivity profile of these glycosides was also analysed based on the global reactivity parameters, including ionisation potential IP, electron affinity EA, chemical potential *µ*, electronegativity *χ*, chemical hardness *η* and electrophilicity index *ω*.^52–55^

Generally, DFT results revealed that all glycoside derivatives possessed enhanced molecular and electronic properties as well as the global reactivity profiles, hence better chemical reactivity and stability for optimal pharmacokinetics and bioavailability,^49^ compared to the aglycones (Table 7). All glycoside derivatives demonstrated significantly more negative values of molecular structure energy *E*, thus, structurally more stable, compared to the aglycones, with the acetylated derivatives (2a-Glc and 4a-Gal) presenting better energy than the deacetylated (2b-Glc and 4b-Gal). In addition, the glycosides, both acetylated and deacetylated derivatives, also demonstrated very comparable values of IP, but better EA values, than the aglycones. This signifies that conjugation of the sugar moiety does not compromise the effectiveness and degradability of curcumin derivatives.

**Table 7:** Molecular structure energy *E*, dipole moment, molecular orbital energy gap and the global reactivity parameters of the glycoside derivatives obtained from DFT calculations

Compound	*E*, a.u.	Energy gap, eV	Dipole moment, debye	IP, eV	EA, eV	*µ*, eV	*ω*, eV	*χ*, eV	*η*, eV
1	−1263.64	3.60	6.08	5.99	2.40	−4.20	4.89	4.20	1.80
2	−1608.03	3.61	4.80	5.89	2.28	−4.09	4.62	4.09	1.81
3	−1722.55	3.40	5.49	5.79	2.39	−4.09	4.92	4.09	1.70
4	−1608.03	3.61	5.22	5.98	2.37	−4.17	4.82	4.17	1.81
2a-Glc	−2828.74	2.93	18.73	5.92	2.99	−4.45	6.77	4.45	1.46
2b-Glc	−2217.87	2.92	24.80	5.84	2.93	−4.39	6.59	4.39	1.46
4a-Gal	−2828.35	2.89	27.19	5.86	2.97	−4.42	6.76	4.42	1.44
4b-Gal	−2217.86	2.90	28.20	5.86	2.96	−4.41	6.72	4.41	1.45

The HOMO–LUMO band gap, which resonates with the chemical reactivity of a compound, showed that all glycosides possessed significantly smaller energy gaps with values lower than 3 eV, more negative chemical potential *µ* and higher electrophilicity *ω*. This suggested that these glycoside derivatives could potentially interact more readily with the target protein. Next, the overall change distribution, portrayed through the higher dipole moment and electronegativity *χ*, further supports that these glycoside compounds possessed enhanced polarity and aqueous solubility than those of the aglycones. Thus, this suggested that these compounds potentially possessed better absorption and distribution in the gastrointestinal tract, hence, enhanced bioavailability.^28,29,43^

Further, while the glycosides demonstrated slightly lower chemical hardness *η* compared to curcumin and the aglycones, the values remained within the acceptable range for drug candidates with balanced reactivity and stability. Therefore, these glycoside derivatives were also expected to remain non-degradable for shelf-life stability.

### HMN binding potential

Additionally, the potential of binding to haemin (HMN), an essential intermediate in the HMN detoxification pathway of *Plasmodium*, as one of the mechanisms of action of antimalarial drugs such as CQ and artemisinin, was also investigated. The HMN released from haemoglobin hydrolysis by the malaria parasite is detoxified into inert hemozoin; interference with this process leads to toxic HMN accumulation and death of the parasite.^56,57^ This harmful HMN detoxification, if left without inhibition, will have the tendency to lead to the formation of reactive oxygen species (ROS). A strong binding of compounds to free HMN can inhibit its detoxification and serve as a validated antimalarial mechanism of action, as exemplified by CQ. Therefore, the binding characteristics of the synthesised compounds with HMN were assessed using the ITC experiments, elucidating key parameters including association constants (*K*_a_), binding ratio (*n*), and changes in enthalpy (Δ*H*), entropy (Δ*S*), and Gibbs free energy (Δ*G*), thereby facilitating a direct comparison with the reference compounds curcumin and CQ.

Among the aglycones, derivative 2 was identified as the most potent for HMN binding that can disrupt the formation of hemozoin based on the most negative and thermodynamically spontaneous Δ*G* (−40.65 ± 1.26 kJ mol^−1^), hence potentially a multi-targeting antimalarial compound (Table 8).^26^ The assessed glycoside compounds demonstrated relatively weaker binding affinity to HMN with *K*_a_ values that are lower than aglycone 2. The observed lower affinities may potentially be due to larger molecular size of the glycosides which leads to weaker overall affinity to HMN. Among the assessed compounds, galactoside derivatives 2a-Gal and 2b-Gal exhibited the highest binding constants, while mannoside derivatives 2a-Man and 2b-Man possessed the lowest, reflecting the sugar-type dependency for potential interaction strengths.

**Table 8:** Thermodynamic properties from the binding of glycoside derivative compounds with HMN from ITC experiments

Compound	*n*	*K*_a_ × 10^6^, M^−1^	Δ*H*, kJ mol^−1^	Δ*S*, J mol^−1^ K^−1^	Δ*G*, kJ mol^−1^
1	0.93 ± 0.12	4.08 ± 2.64	−99.76 ± 0.11	−196.05 ± 5.44	−38.95 ± 1.80
2	0.72 ± 0.03	7.44 ± 3.48	−98.57 ± 1.34	−186.75 ± 8.41	−40.65 ± 1.26
3	0.39 ± 0.13	0.35 ± 0.27	−94.80 ± 1.56	−200.85 ± 1.91	−32.52 ± 2.16
4	0.57 ± 0.24	2.79 ± 1.17	−98.08 ± 1.31	−212.47 ± 7.56	−32.19 ± 1.06
2a-Gal	0.72 ± 0.20	2.60 ± 0.61	−31.19 ± 0.46	−161.45 ± 3.61	−38.05 ± 0.61
2b-Gal	0.56 ± 0.13	1.96 ± 2.61	−98.39 ± 2.28	−230.90 ± 7.78	−34.59 ± 6.34
2a-Glc	0.44 ± 0.16	1.27 ± 0.24	−37.09 ± 3.26	−130.35 ± 4.31	−36.21 ± 0.49
2b-Glc	0.69 ± 0.19	2.51 ± 2.53	−91.12 ± 3.62	−214.55 ± 6.00	−37.09 ± 3.26
2a-Man	1.56 ± 0.41	1.80 ± 0.32	−97.26 ± 3.87	−221.85 ± 1.48	−31.19 ± 0.46
2b-Man	1.82 ± 0.05	1.81 ± 0.57	−89.25 ± 2.08	−172.90 ± 3.39	−31.04 ± 0.66

Despite this, all examined glycosides remained to exhibit strongly exothermic interactions upon binding with HMN based on the negative Δ*H*, Δ*S* and Δ*G*. The enthalpy and entropy change of the glucoside derivatives 2a-Glc and 2b-Glc were also comparable to the aglycone 2 and the most negative among all assessed glycosides. Additionally, all assessed derivatives also demonstrated negative Δ*G* values, with the galactoside derivatives 2a-Gal and 2b-Gal specifically showing the most spontaneously negative values of Δ*G* (−38.05 ± 0.61 kJ mol^−1^ and −37.09 ± 3.26 kJ mol^−1^, respectively), signifying spontaneous and favourable binding interactions.

Nevertheless, the overall results presented enthalpically driven, becoming more ordered systems and spontaneously favourable thermodynamic characteristics of the glycoside derivatives, which reflect conformational stabilisation and enhanced interaction affinity of these compounds upon binding with HMN. In addition, the observed weaker affinity to HMN could also reflect polypharmacological potential of these glycosides from multiple additional mechanisms of action, such as interaction with carbohydrate-binding proteins. Throughout, even though the activity showed selectivity based on the types of conjugated sugars, glycosylation does not compromise the potential of binding to HMN, maintaining the capacity of these derivatives for inhibiting HMN detoxification, validating their promising potential as multi-targeting antimalarial candidates. While glycosylation proved to attenuates HMN binding, glycosides possess improved pharmacokinetic properties which enable interactions with multiple potential target proteins and glycosidases.

## Experimental

### General information

Chemicals and reagents were purchased from commercial sources and used without further purification. Solvents were purchased in gallons and were dried according to the conventional drying method. All reactions were performed in glassware with a Teflon-coated stirring bar. Kieselgel 60 F254 TLC plates (Merck, Germany) were used and visualised under ultraviolet (UV) light at 254 nm and/or by heating after treatment with ethanolic anisaldehyde solution. Purified products were obtained through column chromatography using Kanto silica gel 60N (spherical, neutral, 63–210 µm) or reverse-phase (RP) column chromatography using Waters Sep-Pak® Vac 35 cm^3^ C_10_ 10 g (Waters Corporation, Milford, MA, USA). Purified compounds were characterised and identified by Nuclear Magnetic Resonance (NMR) and high-resolution mass spectrometry (HR-MS). ^1^H and ^13^C NMR spectra were measured in CDCl_3_ and CD_3_OD using Bruker Fourier Transform FT-NMR (400 MHz) (Karlsruhe, Germany) available in the i-CRIM laboratory, Universiti Kebangsaan Malaysia (UKM) or JEOL JNM-ECS-400 spectrometer (Japan) available at the Graduate School of Engineering, Gifu University, Japan and analysed using ACD/Labs NMR Processor software with chemical shifts (*δ*) reported in ppm (parts per million). ^1^H NMR data is presented based on a standardised format, which includes chemical shift (multiplicity, *J* coupling constant, proton number), integration values, and multiplicity designations. HR-MS were obtained on a Waters Xevo Q-TOF mass spectrometer available at the Cooperative Research Facility Division, iGCORE, Gifu University, Japan in electrospray ionisation (ESI) mode. These general materials and procedures were adopted for the synthesis of all compounds, unless otherwise stated.

### Synthetic procedures

#### Synthesis of glycosyl bromides

Glycoside (2.70 mmol, 1.00 eq.) was dissolved in pyridine (2.40 mL) in the presence of acetic anhydride (12.4 mmol, 4.60 eq.). DMAP (2.70 mmol, 1.00 eq.) was added, and the reaction mixture was stirred at RT for 24 hours under argon. On completion (monitored by TLC), the reaction mixture was diluted with dichloromethane and washed from water. Diethyl ether was added to the organic layer and re-extracted from brine. The organic layer was dried over Na_2_SO_4,_ and the solvent was removed under reduced pressure to obtain the acetylated glycosides. The product obtained was used without further purification. Next, the appropriate acetylated glycoside (2.56 mmol, 1.00 eq.) was dissolved in dichloromethane (10.0 mL), followed by a dropwise addition of HBr (33% in acetic acid) (17.9 mmol, 7.00 eq.). The reaction mixture was stirred at RT for 24 hours under argon. On completion (monitored by TLC), the reaction was quenched with saturated NaHCO_3_ and further extracted with dichloromethane from water and brine. The organic layer was dried over Na_2_SO_4,_ and the solvent was removed under reduced pressure. Purified glycosyl bromides 5a–c were obtained by column chromatography on silica gel using a gradient eluent system (hexane : EtOAc = 100 : 0 → 40 : 60, v/v).

#### Synthesis of sugar-conjugated benzaldehyde intermediates

Appropriate glycosyl bromide (5a–c) (0.391 mmol, 1.00 eq.) and benzaldehyde (6–8) (0.782 mmol, 2.00 eq.) were dissolved in dichloromethane (0.500 mL) in the presence of catalyst Bu_4_NBr (0.391 mmol, 1.00eq.). 1 N NaOH (0.781 mmol, 0.782 mL, 2.00 eq.) was added, and the reaction mixture was stirred at RT for 15 hours under argon. On completion (monitored by TLC), the reaction mixture was diluted with ethyl acetate and washed with 1 N NaOH solution, followed by water and brine. Purified yield of intermediates 9a–c, 10a–c, 11a–c were obtained by column chromatography on silica gel using a gradient eluent system (hexane : EtOAc = 80 : 20 → 50 : 50, v/v) and dried under reduced pressure. Purified compounds were characterised and validated by ^1^H NMR and compared with the literature.^38^

#### Synthesis of acetylated glycoside derivatives

Acetylated Knoevenagel condensate curcumin glycosides 2a-Gly, 3a-Gly, 4a-Gly (Gly: Glc, Gal, Man) were synthesised according to the procedure for the synthesis of Knoevenagel condensate derivatives (2–4).^26^ A mixture of curcumin 1 (0.567 mmol, 1.00 eq.) and *O*-glycosylated benzaldehydes 9a–c, 10a–c, 11a–c (1.13 mmol, 2.00 eq.) was dissolved in dry DMF (1.00 mL). A catalytic amount of piperidine and acetic acid was added, and the reaction mixture was stirred at RT for 72 hours under argon. On completion (monitored by TLC), the reaction mixture was diluted with ethyl acetate and washed with water, followed by brine. Purified yields of 2a-Gly, 3a-Gly and 4a-Gly were obtained by column chromatography on silica gel using a gradient eluent system (hexane : EtOAc = 60 : 40 → 90 : 10, v/v) and dried under reduced pressure.

#### Deprotection of the acetylated glycoside derivatives

Appropriate glycoside (2a-Gly, 3a-Gly, 4a-Gly) (0.158 mmol, 1.00 eq.) was dissolved in methanol (4.00 mL). A catalytic amount of NaOMe was added, and the reaction mixture was stirred at RT for 1 hour. On completion (monitored by TLC), the mixture was then neutralised with Dowex 50WX4 resin (H^+^ type), filtered and dried under reduced pressure. The residues were then purified by reverse-phase (RP) C_10_ 10 g column using MeOH as eluent to obtain the deacetylated Knoevenagel condensate curcumin glycoside derivative compounds 2b-Gly, 3b-Gly, 4b-Gly.

### *In vitro* pLDH antiplasmodial assessment

#### *P. falciparum* 3D7 and K1 assays

The antiplasmodial activities of curcumin and the synthesised curcumin derivative compounds were assessed using the *P. falciparum*CQ-sensitive 3D7 and CQ-resistant K1 strains, obtained from the Malaria Research and Reference Reagent Resource Center (MR4), Manassas, Virginia. The parasites were revived from cryopreservation and cultured at 1% haematocrit using purified O+ human erythrocytes in RPMI 1640 medium supplemented with 0.5% Albumax I (GIBCO, Life Technologies, USA), 25 mM HEPES, 100 µM hypoxanthine, 12.5 µg per mL gentamicin, and 1.77 mM sodium bicarbonate. Cultures were maintained at 37 °C in a 5% CO_2_ atmosphere. Parasitaemia was assessed by microscopic examination of field-stained thin blood smears. Parasite sensitivity to curcumin was evaluated using the parasite lactate dehydrogenase (pLDH) assay, as described by Makler *et al.*,^58^ in flat-bottom 96-well microtiter plates. Prior to the assay, parasite cultures were synchronised using 5% d-sorbitol and plated at 2% haematocrit and 2% parasitaemia, predominantly in the ring stage. Curcumin derivatives were added at predetermined concentrations. Following a one-hour incubation at RT in the dark, colour development was measured at 655 nm using a Fluostar Optima microplate reader. Data were analysed *via* non-linear regression using GraphPad Prism 5 (GraphPad Software, Inc., San Diego, CA) to determine the 50% effective concentration (EC_50_).

#### Cytotoxicity MTT assay

Human normal liver cell line WRL-68 (ATCC: CL-48) was obtained from the American Type Culture Collection (ATCC), USA. Cytotoxicity of curcumin derivatives was assessed using the MTT assay, as described by Mosmann.^59^ Briefly, 100 µL of WRL-68 cells (2 × 10^4^ cells per mL) in complete medium supplemented with 10% foetal bovine serum (FBS) was seeded into 96-well flat-bottom microtiter plates. Cells were incubated with or without curcumin derivatives for 48 hours at 37 °C in a 5% CO_2_ atmosphere. After incubation, confluent cells were treated with curcumin derivatives at concentrations ranging from 10 to 0.01 µg mL^−1^. Wells containing untreated cells served as positive controls for cell viability. Following the treatment, 5 mg per mL MTT solution in phosphate-buffered saline (MTT–PBS) was added to each well, and the plates were incubated for an additional 3 hours under the same conditions. The medium was then removed and replaced with 100 µL of dimethyl sulfoxide (DMSO) per well to solubilise the formazan crystals, followed by a 10 minute incubation at 37 °C in a CO_2_ incubator. Absorbance was measured at 540 nm using a Fluorostar OPTIMA microplate reader. The 50% cytotoxic concentration (CC_50_) of the curcumin derivatives was determined from three independent experiments. Percentage growth inhibition and CC_50_ values were calculated by non-linear regression analysis using GraphPad Prism. Cytotoxicity was reported as the concentration required to inhibit 50% of cell growth.

#### Selectivity index (SI) and resistance index (RI)

The selectivity index (SI) was calculated as the ratio of the 50% cytotoxic concentration (CC_50_) to the 50% inhibitory concentration (IC_50_) of antimalarial activity (SI = CC_50_/IC_50_), based on data from MTT and pLDH assays. Compounds with SI values greater than 100 (SI > 100) were considered strong and selective antiplasmodial agents, as described by Sarr *et al.*^60^ Additionally, the resistance index (RI) was determined by calculating the ratio of IC_50_ values obtained from the resistant strain (K1) to those from the sensitive strain (3D7) (RI = IC_50_ K1/IC_50_ 3D7). A higher RI indicates reduced sensitivity of the resistant strain to the test compound.

### *In silico* assessments

#### Molecular docking

The design of curcumin derivative structures utilised ChemDraw Pro 20.0,^61^ Chem3D Ultra 20.0,^62^ and MarvinSketch 23.14.0,^63^ with the structures subsequently optimised to the corresponding minimum-energy geometries of each structure. Further, all prepared ligands were readied for molecular docking simulation using Autodock 1.5.7 and Open Babel, which were then saved in .pdb and .pdbqt file formats.^64,65^ The 3D X-ray crystallographic structure of GSK-3β was retrieved from the Protein Data Bank (PDB ID 1Q5K, resolution 1.94 Å, https://www.rcsb.org).^66^ It was prepared for molecular docking by removing water molecules and adding polar hydrogen as well as Kollman charges, before being saved as a .pdbqt file.^67^ The docking analysis was performed to screen curcumin derivatives that most effectively bind to GSK-3β while evaluating the optimal energy geometry of the docked compounds. Using the previously validated active site of the protein, the docking simulation was performed with a defined grid box dimension of 40 × 40 × 40 Å centred at *x* = 23.9226, *y* = 21.6961, and *z* = 9.76081.^67,68^ The active site of the protein is specified as the cavity that binds specific ligands (curcumin derivatives), which may lead to potential inhibition of its activity. The other docking parameters were set to default.^68^ Analysis of the docking results revealed the binding affinity, the types of molecular interactions and the amino acids involved in the binding, and the inhibition constant, *K*_i_. BIOVIA Discovery Studio Visualizer^69^ and Ligplot+^70^ were utilised to map the interactions and visualise the position and distance of the amino acid residues interacting with the ligands.

#### Physicochemical ADMET screening

Physicochemical analysis is useful to predict the absorption, distribution, metabolism, excretion and toxicity (ADMET) profiles of a compound, which is also an alternative method for screening a long list of compounds. The ADMET data forecasts the overall physicochemical behaviour of potential drug candidates within the body, including the blood, tissues, and organs, thus providing supplementary screening data to evaluate the possible effects of a compound upon administration into the body. The structures of all derivatives were drawn using ChemDraw Pro 20.0,^61^ to generate the Simplified Molecular Input Line Entry System (SMILES). The ADMET assessments were prompted and performed by inputting the generated SMILES into the web servers SwissADME (https://www.swissadme.ch/index.php), pkCSM (https://biosig.unimelb.edu.au/pkcsm/), and proTox-II (https://tox.charite.de/protox_II). The predicted ADMET properties of curcumin were compared with those of the derivatives.^9,71–73^

#### Density functional theory (DFT) geometry optimisation

The DFT analysis provides critical information on energy minimisation related to bonding, dipole moment, molecular orbital (MO) energy profiles and global reactivity parameters from geometry optimisation calculations. Theoretically, it enables geometry optimisation of a chemical structure in order to determine its stable geometry using a quantum chemical technique.^74^ The DFT geometry optimisation utilised Orca 6.0.^75–78^ The calculations were set at the B3LYP/6-311G++(d,p) level and employed for DMSO solvent phase calculations, which were adopted from the previously reported method by Hazarika and Kalita.^49^ The 3D structures of all derivative compounds were prepared in .sdf format and energy-minimised using Avogadro 1.2.0,^79,80^ to obtain the compounds' coordinates in .xyz format and generate each respective input .inp file. The generated output files in .out format were visualised and analysed using Avogadro. The utilisation of Orca was driven by computational efficiency. While the commonly used Gaussian/GaussView software for DFT calculations offers a simpler interface for calculation setup and visualisation of compounds, Orca benefits by providing better scalability and calculation capacity for larger molecules without compromising the expected outputs. This makes it preferable for reducing the simulation time when calculating extended conjugated frameworks of glycoside compounds.

### HMN binding assessment

#### Isothermal titration calorimetry (ITC) experiments

An ITC microcalorimeter (TA Instruments, New Castle, DE, USA) was utilised to assess the interaction of the synthesised curcumin derivative compounds with HMN at 37 °C as described by Feroz *et al.*^81^ Curcumin and CQ were used as reference compounds to enable comparative analysis of binding affinities and thermodynamic parameters. The synthesised compounds were dissolved in acetone at concentrations ranging from 1 to 1.5 mg mL^−1^ to prepare stock solutions, while the HMN stock solution (0.5 mg mL^−1^) was prepared by dissolving hemin in 0.5 M NaOH. The stock solutions were subsequently diluted with 10 mM sodium phosphate buffer (pH 7.4), resulting in the final samples containing 0.5 M NaOH and about 3% acetone. All samples were vacuum-degassed for 10 minutes before the titration experiments. For the titration, 20 µM hemin was placed in the sample cell, with the reference cell containing deionised water. The hybrid compound (100 µM) was loaded into a 250 µL syringe and introduced into the microcalorimeter for injection. The titration required 16 consecutive 15 µL injections into the sample cell, spaced each 400 s intervals, with stirring kept at 200 rpm to ensure adequate mixing. In the data analysis, the heat effects from solution mixing and dilution were manually corrected. An independent binding model was employed to process and analyse the data using NanoAnalyze software (v3.3.0). The results are presented as the average ± standard deviation of the selected replicates showing the best consistency.

## Conclusions

The exploration of sugar-conjugated compounds through glycosylation has gained significant attention in research for the discovery and development of promising bioactive compounds against malaria. This study has designed and synthesised novel sugar-conjugated Knoevenagel condensate curcumin derivatives. All designed compounds were successfully synthesised through *O*-glycosylation except for mannoside derivatives of 4, where orthoester-linked intermediate 11c and the subsequent glycosides 4a-Man and 4b-Man were generated. Further, this work presented their efficacy against *P. falciparum*CQ-sensitive 3D7 and multi-drug-resistant K1 assays, followed by GSK-3β inhibition potential from molecular docking analysis, physicochemical properties from ADMET assessment, electronic and reactivity profiles from DFT calculations and HMN binding potential from ITC experiments. These assessments have demonstrated the influence of conjugated sugar moiety on bioavailability, efficacy and safety. Particularly, the acetylated glucoside 2a-Glc and galactoside 4a-Gal demonstrated superior antiplasmodial activity against the pLDH assays and improved cytotoxic potential, relative to the aglycones and curcumin. Deprotection of the acetylated compounds, yielding the deacetylated glycosides containing preserved free hydroxy groups, has, however, demonstrated reduced efficacy, except for the orthoester-linked 4a-Man and 4b-Man. All assessed glycosides, particularly 2a-Glc, potentially inhibit GSK-3β more strongly than the aglycones and curcumin, while also demonstrating thermodynamically spontaneous HMN binding. This exemplifies the multi-targeting potential of the glycosides derived from curcumin in mediating malaria infections. Additionally, these derivatives also generally possessed favourable physicochemical properties based on the ADMET analysis, along with improvements in the electronic and reactivity properties. This further supports the benefits of glycosylation strategies and the influence of sugar for developing effective antimalarial compounds with optimal bioavailability and bioactivity.

## Author contributions

SNHJ contributed to conceptualisation, methodology planning, data curation, formal analysis, writing and editing the original draft. AHA, YHN and NFNM were involved in conceptualisation, methodology planning, writing, reviewing and editing the original draft. NO involved in funding acquisition, conceptualisation, methodology planning, reviewing and editing the draft, providing resources and laboratory access, supervising the progress, and data validation. SRF and SDL involved in conceptualisation, methodology planning, reviewing and editing the draft, providing resources and access to software, supervising the progress, and data validation. FM and YZ provided access to resources and supervised the progress. JL contributed to funding acquisition, project administration, access to resources, overall supervision, data validation, and reviewing and editing the draft. All authors have reviewed the manuscript.

## Conflicts of interest

There are no conflicts of interest to declare.

## Supplementary Material

RA-016-D5RA09343K-s001

## Data Availability

The data supporting this article have been included as part of the supplementary information (SI). Supplementary information is available. See DOI: https://doi.org/10.1039/d5ra09343k.
