# Supplementary material for: Novel sugar-conjugated Knoevenagel condensate curcumin derivatives as promising bioactive hybrids with antimalarial potential
Source: RSC Adv. 2026 May 12;16(27):24689–703. doi: 10.1039/d5ra09343k (PMC13162220; doi:10.1039/d5ra09343k)
Supplement: RA-016-D5RA09343K-s001 [file RA-016-D5RA09343K-s001.pdf]

Supplementary Information (SI) for RSC Advances.
This journal is © The Royal Society of Chemistry 2026

Novel sugar-conjugated Knoevenagel condensate curcumin derivatives as promising bioactive hybrids with antimalarial potential

Siti Nur Hidayah Jamil,*[a,b] Natsuhisa Oka,*[a,c,d,e] Amatul Hamizah Ali,[b] Yan Hong Ng,[f] Nur Fatin Najihah Marzuki,[g] Shevin Rizal Feroz,[f,h] Su Datt Lam,[h,I,j] Fauze Mahmud,[k,l] Yusmazura Zakaria [g] and Jalifah Latip *[b,m]

[a.] International Joint Department of Materials Science and Engineering between National University of Malaysia and Gifu University, Graduate School of Engineering, Gifu University, 1-1 Yanagido, Gifu 501-1193, Japan

[b.] Department of Chemical Sciences, Faculty of Science and Technology, Universiti Kebangsaan Malaysia, UKM Bangi 43600, Selangor, Malaysia

[c.] Department of Chemistry and Biomolecular Science, Faculty of Engineering, Gifu University, 1-1 Yanagido, Gifu 501-1193, Japan

[d.] Center for One Medicine Innovative Translational Research (COMIT), Institute for Advanced Study, Gifu University, 1-1 Yanagido, Gifu 501-1193, Japan

[e.] Institute for Glyco-core Research (iGCORE), Gifu University, Gifu 501-1193, Japan

[f.] Department of Biological Sciences and Biotechnology, Faculty of Science and Technology, Universiti Kebangsaan Malaysia, UKM Bangi 43600, Selangor, Malaysia

[g.] Biomedicine Programme, School of Health Sciences, Health Campus, Universiti Sains Malaysia, Kota Bharu 16150, Kelantan, Malaysia

[h.] Structural Biology and Protein Engineering Research Group, Universiti Kebangsaan Malaysia, Bangi 43600, Selangor, Malaysia

[i.] Department of Applied Physics, Faculty of Science and Technology, Universiti Kebangsaan Malaysia, UKM Bangi 43600, Selangor, Malaysia

[j.] Center for Global Health Research (CGHR), Saveetha Medical College, Saveetha Institute of Medical and Technical Sciences (SIMATS), Saveetha University, Chennai 602 105, Tamil Nadu, India

[k.] Faculty of Science and Technology, Universiti Malaysia Sabah, Jalan UMS, Kota Kinabalu 88400, Sabah, Malaysia

[l.] BioAgriTech Research (BioATR) Group, Faculty of Science and Technology, Universiti Malaysia Sabah, Jalan UMS, Kota Kinabalu 88400, Sabah, Malaysia

[m.] Smart Material and Sustainable Product Innovation (SMatSPIn) Research, Universiti Kebangsaan Malaysia, UKM Bangi 43600, Selangor, Malaysia

** Corresponding author: Siti Nur Hidayah Jamil, Email: sitinh.jamil@gmail.com; Prof. Dr. Natsuhisa Oka, Email: oka.natsuhisa.f9@f.gifu-u.ac.jp; Assoc. Prof. Dr. Jalifah Latip, Email: jalifah@ukm.edu.my.*

Supplementary Information

Table of contents

1. Characterisation of the synthesised sugar-conjugated derivatives S1
2. NMR spectra of the synthesised sugar-conjugated derivatives S6
3. *In vitro* pLDH antiplasmodial activity S29
4. Cytotoxicity from MTT WRL-68 assay S32
5. Binding interactions to GSK-3β from molecular docking S34
6. DFT geometry optimisation analysis S39
7. Haemin binding potential S43
8. References S44

1. Characterisation of the synthesised sugar-conjugated derivatives

1.1 General information

Chemicals and reagents were purchased from commercial sources and used without further purification. Solvents were purchased in gallons and were dried according to the established procedure [31]. All reactions were performed in glassware with a Teflon-coated stirring bar. Kieselgel 60 F254 TLC plates (Merck, Germany) were used and visualised under ultraviolet (UV) light at 254 nm and/or by heating after treatment with ethanolic anisaldehyde solution. Purified products were obtained through column chromatography using Kanto silica gel 60N (spherical, neutral, 63–210 µm) or reverse-phase (RP) column chromatography using Waters Sep-Pak® Vac 35cc C_{10} 10g (Waters Corporation, Milford, MA, USA). Purified compounds were characterised and identified by Nuclear Magnetic Resonance (NMR) and high-resolution mass spectrometry (HR-MS). ^{1}H and ^{13}C NMR spectra were measured in $CDCl_3$ and CD_3OD using Bruker Fourier Transform FT-NMR (400 MHz) (Karlsruhe, Germany) available in the i-CRIM laboratory, Universiti Kebangsaan Malaysia (UKM) or JEOL JNM-ECS-400 spectrometer (Japan) available at the Graduate School of Engineering, Gifu University, Japan and analysed using ACD/Labs NMR Processor software with chemical shifts (δ) reported in parts per million (ppm). ^{1}H NMR data is presented based on a standardised format, which includes chemical shift (multiplicity, *J* coupling constant, proton number), integration values, and multiplicity designations. Multiplicity is indicated as follows: s = singlet, d = doublet, t = triplet, q = quartet, br = broad, dd = doublet of doublet, dt = doublet of triplet, ddd = doublet of doublet of doublet, dq = doublet of quartet, tdd = triplet of doublet of doublet, m = multiplet. HR-MS were obtained on a Waters Xevo D-TOF mass spectrometer available at the Graduate School of Engineering, Gifu University, Japan in electrospray ionisation (ESI) mode.

1.2 Sugar-conjugated benzaldehyde intermediates

(2*R*,3*S*,4*S*,5*R*,6*S*)-2-(acetoxymethyl)-6-(4-formylphenoxy)tetrahydro-2*H*-pyran-3,4,5-triyltriacetate (9a)
This compound was obtained as yellowish concentrate (0.165 g, 0.364 mmol, 93%). ^{1}H-NMR (400 MHz, $CDCl_3$) δ (ppm): 9.85 (s, 1H), 7.80–7.76 (m, *J* = 9.2 Hz, 2H), 7.06–7.01 (m, *J* = 8.7 Hz, 2H), 5.30–5.20 (m, 2H), 5.20–5.14 (m, 1H), 5.14–5.08 (m, 1H), 4.22 (dd, *J* = 5.5, 12.4 Hz, 1H), 4.11 (dd, *J* = 2.3, 12.4 Hz, 1H), 3.92–3.86 (m, 1H), 2.00 (s, 3H), 1.98 (s, 3H), 1.97 (s, 3H), 1.96 (s, 3H).

(2*R*,3*R*,4*S*,5*R*,6*S*)-2-(acetoxymethyl)-6-(4-formylphenoxy)tetrahydro-2*H*-pyran-3,4,5-triyltriacetate (9b)
This compound was obtained as yellowish concentrate (0.129 g, 0.285 mmol, 73%). ^{1}H-NMR (400 MHz, $CDCl_3$) δ (ppm): 9.93 (s, 1H), 7.88–7.84 (m, *J* = 8.7 Hz, 2H), 7.14–7.09 (m, *J* = 8.7 Hz, 2H), 5.37–5.28 (m, 2H), 5.26–5.23 (m, 1H), 5.22–5.16 (m, 1H), 4.30 (dd, *J* = 5.5, 12.4 Hz, 1H), 4.19 (dd, *J* = 2.8, 12.4 Hz, 1H), 3.99–3.93 (m, 1H), 2.08 (s, 3H), 2.07 (s, 3H), 2.07 (s, 3H), 2.05 (s, 3H)

(2*R*,3*R*,4*S*,5*S*,6*S*)-2-(acetoxymethyl)-6-(4-formylphenoxy)tetrahydro-2*H*-pyran-3,4,5-triyl triacetate (9c)
This compound was obtained as yellowish concentrate (0.0619 g, 0.137 mmol, 35%). ^{1}H-NMR (400 MHz, $CDCl_3$) δ (ppm): 9.90 (s, 1H), 7.81–7.76 (m, *J* = 8.7 Hz, 2H), 7.25–7.21 (m, 2H), 5.45 (dd, *J* = 1.4, 2.8 Hz, 1H), 5.36–5.21 (m, 2H), 5.14–5.08 (m, 1H), 4.43–4.37 (m, 1H), 4.24–4.18 (m, 1H), 3.72–3.66 (m, 1H), 2.10 (s, 3H), 2.05 (s, 3H), 2.03 (s, 3H), 1.86 (s, 3H).

(2*R*,3*S*,4*S*,5*R*,6*S*)-2-(acetoxymethyl)-6-(4-formyl-2-methoxyphenoxy)tetrahydro-2*H*-pyran-3,4,5-triyltriacetate (10a)
This compound was obtained as yellowish concentrate (0.160 g, 0.332 mmol, 85%). ^{1}H-NMR (400 MHz, $CDCl_3$) δ (ppm): 9.84 (s, 1H), 7.38 (s, 1H), 7.36 (d, *J* = 1.4 Hz, 1H), 7.19 (d, *J* = 8.7 Hz, 1H), 5.50 (dd, *J* = 8.0, 10.3 Hz, 1H), 5.41 (d, *J* = 3.2 Hz, 1H), 5.08 (dd, *J* = 3.4, 10.3 Hz, 1H), 5.02 (d, *J* = 7.8 Hz, 1H), 4.21–4.09 (m, 2H), 4.05–4.02 (m, 1H), 3.84 (s, 3H), 2.12 (s, 3H), 2.02 (s, 3H), 2.00 (s, 3H), 1.97 (s, 3H).

(2*R*,3*R*,4*S*,5*R*,6*S*)-2-(acetoxymethyl)-6-(4-formyl-2-methoxyphenoxy)tetrahydro-2*H*-pyran-3,4,5-triyltriacetate (10b)
This compound was obtained as yellowish concentrate (0.121 g, 0.250 mmol, 64%). ^{1}H-NMR (400 MHz, $CDCl_3$) δ (ppm): 9.90 (s, 1H), 7.44 (s, 1H), 7.43–7.40 (m, 1H), 7.22 (d, *J* = 7.8 Hz, 1H), 5.34–5.30 (m, 2H), 5.21–5.15 (m, 1H), 5.14–5.10 (m, 1H), 4.28 (dd, *J* = 5.0, 12.4 Hz, 1H), 4.19 (dd, *J* = 2.8, 12.4 Hz, 1H), 3.90 (s, 3H), 3.88–3.84 (m, 1H), 2.08 (s, 3H), 2.08 (s, 3H), 2.06 (s, 3H), 2.05 (s, 3H).

(2*R*,3*R*,4*S*,5*S*,6*S*)-2-(acetoxymethyl)-6-(4-formyl-2-methoxyphenoxy)tetrahydro-2*H*-pyran-3,4,5-triyltriacetate (10c)

This compound was obtained as yellowish concentrate (0.0490 g, 0.102 mmol, 26%). ^{1}H-NMR (400 MHz, $CDCl_3$) δ (ppm): 9.78 (s, 1H), 7.32 (d, *J* = 1.4 Hz, 1H), 7.30–7.28 (m, 1H), 7.25 (d, *J* = 5.0 Hz, 1H), 5.44 (d, *J* = 2.8 Hz, 1H), 5.25–5.20 (m, 1H), 5.18 (d, *J* = 9.6 Hz, 1H), 5.10–5.05 (m, 1H), 4.48 (dd, *J* = 2.8, 4.1 Hz, 1H), 4.15–4.10 (m, 1H), 3.78 (s, 3H), 3.67–3.61 (m, 1H), 2.01 (s, 3H), 1.95 (s, 3H), 1.94 (s, 3H), 1.92 (s, 3H).

(2*R*,3*S*,4*S*,5*R*,6*S*)-2-(acetoxymethyl)-6-(3-formylphenoxy)tetrahydro-2*H*-pyran-3,4,5-triyltriacetate (11a)

This compound was obtained as yellowish concentrate (0.166 g, 0.368 mmol, 94%). ^{1}H-NMR (400 MHz, $CDCl_3$) δ (ppm): 9.96 (s, 1H), 7.56 (dd, *J* = 0.9, 7.3 Hz, 1H), 7.49–7.48 (m, 1H), 7.47–7.43 (m, 1H), 7.24 (tdd, *J* = 1.3, 2.4, 8.2 Hz, 1H), 5.48 (dd, *J* = 8.0, 10.3 Hz, 1H), 5.40 (d, *J* = 3.2 Hz, 1H), 5.07 (dd, *J* = 3.4, 10.3 Hz, 1H), 5.01 (d, *J* = 7.8 Hz, 1H), 4.20–4.07 (m, 2H), 4.05–4.00 (m, 1H), 2.11 (s, 3H), 2.01 (s, 3H), 1.98 (s, 3H), 1.95 (s, 3H).

(2*R*,3*R*,4*S*,5*R*,6*S*)-2-(acetoxymethyl)-6-(3-formylphenoxy)tetrahydro-2*H*-pyran-3,4,5-triyltriacetate (11b)

This compound was obtained as yellowish concentrate (0.133 g, 0.293 mmol, 75%). ^{1}H-NMR (400 MHz, $CDCl_3$) δ (ppm): 9.97 (s, 1H), 7.59 (dd, *J* = 0.9, 7.3 Hz, 1H), 7.52–7.51 (m, 1H), 7.51–7.46 (m, 1H), 7.28–7.24 (m, 1H), 5.36–5.27 (m, 2H), 5.21–5.13 (m, 2H), 4.30–4.23 (m, 1H), 4.23–4.17 (m, 1H), 3.99–3.91 (m, 1H), 2.10 (s, 3H), 2.07 (s, 3H), 2.06 (s, 3H), 2.04 (s, 3H).

(3a*S*,5*R*,6*R*,7*S*,7a*S*)-5-(acetoxymethyl)-2-(3-formylphenoxy)-2-methyltetrahydro-5*H*-[1,3]dioxolo[4,5-b]pyran-6,7-diyl diacetate (11c)

This compound was obtained as yellowish concentrate (0.0566 g, 0.125 mmol, 32%). ^{1}H-NMR (400 MHz, $CDCl_3$) δ (ppm): 9.82 (s, 1H), 7.18 (dd, *J* = 1.8, 3.7 Hz, 1H), 7.16–7.14 (m, 1H), 7.05–7.03 (m, 1H), 7.01 (dd, *J* = 2.1, 8.5 Hz, 1H), 5.23 (t, *J* = 9.6 Hz, 1H), 5.19 (d, *J* = 2.8 Hz, 1H), 4.92 (dd, *J* = 3.9, 9.9 Hz, 1H), 4.21–4.16 (m, 1H), 4.10–4.07 (m, 1H), 4.04 (dd, *J* = 2.5, 3.9 Hz, 1H), 3.51 (ddd, *J* = 3.0, 5.0, 9.4 Hz, 1H), 2.12 (s, 3H), 2.06 (s, 3H), 2.03 (s, 3H), 1.60 (s, 3H).

1.3 Acetylated glycoside derivatives

(2*R*,3*S*,4*S*,5*R*,6*S*)-2-(acetoxymethyl)-6-(4-((*E*)-5-(4-hydroxy-3-methoxyphenyl)-2-((*E*)-3-(4-hydroxy-3-methoxyphenyl)acryloyl)-3-oxopenta-1,4-dien-1-yl)phenoxy)tetrahydro-2*H*-pyran-3,4,5-triyl triacetate (2a-Gal)

This compound was obtained as orange solids (0.346 g, 0.431 mmol, 76%), m.p.: 210–212 °C. ^{1}H-NMR (400 MHz, $CDCl_3$) δ (ppm): 7.77 (s, 1H), 7.72 (d, *J* = 15.6 Hz, 1H), 7.47–7.40 (m, 3H), 7.12 (dd, *J* = 1.6, 8.5 Hz, 1H), 7.02–6.97 (m, 2H), 6.96–6.82 (m, 6H), 6.79 (s, 1H), 6.30–6.17 (m, 2H), 5.29–5.19 (m, 1H), 5.16–5.05 (m, 2H), 4.24 (dd, *J* = 5.3, 12.1 Hz, 2H), 4.13–4.06 (m, 2H), 3.87 (s, 3H), 3.84 (s, 3H), 2.02 (s, 3H), 2.01 (s, 3H), 2.00 (s, 3H), 1.99 (s, 3H). ^{13}C-NMR (100 MHz, $CDCl_3$) δ (ppm): 198.4, 186.8, 170.6, 170.3, 169.5, 169.4, 158.2, 149.1, 148.7, 147.8, 146.9, 146.9, 145.7, 139.8, 139.8, 139.7, 132.3, 128.6, 127.3, 126.6, 125.3, 124.2, 123.8, 119.7, 117.0, 115.0, 114.9, 110.5, 110.0, 98.4, 72.7, 72.2, 71.1, 68.2, 61.9, 56.2, 56.1, 20.7, 20.7, 20.7, 20.7. HRMS (ESI-TOF) *m/z*: [M + H]$^+$ calculated for $C_{42}H_{42}O_{16}$: 803.2546, found 803.2531.

(2*R*,3*R*,4*S*,5*R*,6*S*)-2-(acetoxymethyl)-6-(4-((*E*)-5-(4-hydroxy-3-methoxyphenyl)-2-((*E*)-3-(4-hydroxy-3-methoxyphenyl)acryloyl)-3-oxopenta-1,4-dien-1-yl)phenoxy)tetrahydro-2*H*-pyran-3,4,5-triyl triacetate (2a-Glc)

This compound was obtained as orange solids (0.355 g, 0.442 mmol, 78%), m.p.: 212–213 °C. ^{1}H-NMR (400 MHz, $CDCl_3$) δ (ppm): 7.79 (s, 1H), 7.75 (d, *J* = 15.3 Hz, 1H), 7.49–7.43 (m, 3H), 7.15 (dd, *J* = 2.0, 8.3 Hz, 1H), 7.03 (dt, *J* = 2.0, 4.2 Hz, 2H), 6.97 (d, *J* = 1.8 Hz, 1H), 6.95–6.86 (m, 5H), 6.79 (d, *J* = 16.2 Hz, 1H), 6.00 (s, 2H), 5.49–5.42 (m, 2H), 5.11–5.06 (m, 1H), 5.05 (d, *J* = 7.6 Hz, 1H), 4.22–4.16 (m, 1H), 4.15–4.08 (m, 2H), 3.91 (s, 3H), 3.89 (s, 3H), 2.16 (s, 3H), 2.02 (s, 3H), 2.02 (s, 3H), 2.00 (s, 3H). ^{13}C-NMR (100 MHz, $CDCl_3$) δ (ppm): 198.4, 186.8, 170.4, 170.3, 170.2, 169.4, 158.2, 149.1, 148.7, 147.8, 146.9, 146.8, 145.6, 139.8, 139.7, 132.3, 128.6, 127.3, 126.6, 125.3, 124.2, 123.8, 119.8, 117.0, 115.0, 114.9, 110.5, 110.0, 98.9, 71.2, 70.8, 68.5, 66.8, 61.3, 56.2, 56.1, 20.8, 20.7, 20.7, 20.6. HRMS (ESI-TOF) *m/z*: [M + Na]$^+$ calculated for $C_{42}H_{42}O_{16}$: 825.2365, found 825.2355.

(2*R*,3*R*,4*S*,5*S*,6*S*)-2-(acetoxymethyl)-6-(4-((*E*)-5-(4-hydroxy-3-methoxyphenyl)-2-((*E*)-3-(4-hydroxy-3-methoxyphenyl)acryloyl)-3-oxopenta-1,4-dien-1-yl)phenoxy)tetrahydro-2*H*-pyran-3,4,5-triyl triacetate (2a-Man)

This compound was obtained as orange solids (0.182 g, 0.227 mmol, 40%), m.p.: 211–213 °C. ^{1}H-NMR (400 MHz, $CDCl_3$) δ (ppm): 7.78 (s, 1H), 7.75 (d, *J* = 15.1 Hz, 1H), 7.49–7.43 (m, 3H), 7.15 (dd, *J* = 2.1, 8.5 Hz, 1H), 7.04–7.01 (m, 2H), 6.97 (d, *J* = 1.8 Hz, 1H), 6.94–6.86 (m, 6H), 6.78 (d, *J* = 16.0 Hz, 1H), 6.05 (s, 1H), 6.09 (s, 1H), 5.28–5.24 (m, 2H),

5.17–5.11 (m, 1H), 5.09 (d, *J* = 7.8 Hz, 1H), 4.26 (dd, *J* = 5.5, 12.4 Hz, 1H), 4.16–4.09 (m, 2H), 3.92 (s, 3H), 3.89 (s, 3H), 2.04 (s, 3H), 2.02 (s, 3H), 2.02 (s, 3H). ^{13}C-NMR (100 MHz, $CDCl_3$) δ (ppm): 197.7, 186.7, 170.7, 170.2, 169.4, 169.3, 156.9, 149.0, 148.7, 147.8, 146.9, 146.8, 146.0, 141.6, 139.5, 135.1, 129.9, 127.1, 126.4, 125.3, 125.0, 124.2, 123.8, 119.4, 119.3, 117.1, 114.9, 110.4, 109.9, 98.8, 72.6, 70.9, 67.9, 61.6, 60.4, 56.0, 56.0, 20.7, 20.6, 20.5, 20.5. HRMS (ESI-TOF) *m/z*: [M + Na]$^+$ calculated for $C_{42}H_{42}O_{16}$: 825.2365, found 825.2348.

(2*R*,3*S*,4*S*,5*R*,6*S*)-2-(acetoxymethyl)-6-(4-((*E*)-5-(4-hydroxy-3-methoxyphenyl)-2-((*E*)-3-(4-hydroxy-3-methoxyphenyl)acryloyl)-3-oxopenta-1,4-dien-1-yl)-2-methoxyphenoxy)tetrahydro-2*H*-pyran-3,4,5-triyl triacetate (3a-Gal)

This compound was obtained as orange solids (0.274 g, 0.329 mmol, 58%), m.p.: 203–204 °C. ^{1}H-NMR (400 MHz, $CDCl_3$) δ (ppm): 7.78–7.71 (m, 2H), 7.47 (d, *J* = 16.0 Hz, 1H), 7.15 (dd, *J* = 1.6, 8.0 Hz, 1H), 7.06–7.00 (m, 5H), 6.98–6.86 (m, 4H), 6.78 (d, *J* = 16.0 Hz, 1H), 5.99 (s, 1H), 6.02 (s, 1H), 5.48 (dd, *J* = 8.0, 10.3 Hz, 1H), 5.42 (d, *J* = 3.2 Hz, 1H), 5.07 (dd, *J* = 3.2, 10.5 Hz, 1H), 4.92 (d, *J* = 7.8 Hz, 1H), 4.21–4.15 (m, 2H), 4.15–4.10 (m, 2H), 3.91 (s, 3H), 3.89 (s, 3H), 3.76 (s, 3H), 2.15 (s, 3H), 2.03 (s, 3H), 2.01 (s, 3H), 2.00 (s, 3H). ^{13}C-NMR (100 MHz, $CDCl_3$) δ (ppm): 198.4, 186.9, 170.5, 170.3, 170.2, 169.5, 150.3, 149.2, 148.8, 147.9, 147.8, 147.0, 146.9, 145.7, 140.2, 140.0, 129.8, 127.3, 126.5, 125.1, 124.2, 124.0, 123.8, 119.7, 118.9, 115.1, 115.0, 114.3, 110.6, 110.1, 100.7, 71.1, 70.7, 68.6, 66.9, 61.3, 56.1, 56.1, 56.0, 21.1, 20.8, 20.7, 20.7. HRMS (ESI-TOF) *m/z*: [M + Na]$^+$ calculated for $C_{43}H_{43}O_{17}$: 855.247, found 855.2452.

(2*R*,3*R*,4*S*,5*R*,6*S*)-2-(acetoxymethyl)-6-(4-((*E*)-5-(4-hydroxy-3-methoxyphenyl)-2-((*E*)-3-(4-hydroxy-3-methoxyphenyl)acryloyl)-3-oxopenta-1,4-dien-1-yl)-2-methoxyphenoxy)tetrahydro-2*H*-pyran-3,4,5-triyl triacetate (3a-Glc)

This compound was obtained as orange solids (0.312 g, 0.442 mmol, 66%), m.p.: 204–206 °C. ^{1}H-NMR (400 MHz, $CDCl_3$) δ (ppm): 7.78–7.71 (m, 2H), 7.46 (d, *J* = 16.0 Hz, 1H), 7.15 (dd, *J* = 1.8, 8.7 Hz, 1H), 7.05–7.00 (m, 5H), 6.96–6.86 (m, 5H), 6.77 (d, *J* = 16.0 Hz, 1H), 6.06 (s, 1H), 6.09 (s, 1H), 5.23–5.27 (m, 2H), 5.13 (t, *J* = 9.6 Hz, 1H), 4.99–4.94 (m, 1H), 4.24 (dd, *J* = 5.0, 12.4 Hz, 1H), 4.15–4.08 (m, 2H), 3.90 (s, 3H), 3.88 (s, 3H), 3.74 (s, 3H), 2.03 (s, 3H), 2.02 (s, 6H), 2.01 (s, 3H). ^{13}C-NMR (100 MHz, $CDCl_3$) δ (ppm): 198.3, 186.9, 170.7, 170.3, 169.5, 169.4, 150.4, 149.1, 148.7, 147.8, 147.7, 147.0, 146.9, 145.7, 140.3, 139.9, 129.9, 127.3, 126.5, 125.2, 124.2, 124.0, 123.8, 119.7, 119.2, 115.0, 115.0, 114.3, 110.5, 110.0, 100.1, 72.5, 72.2, 71.1, 68.3, 61.9, 56.1, 56.1, 56.1, 20.7, 20.7, 20.7, 20.7. HRMS (ESI-TOF) *m/z*: [M + Na]$^+$ calculated for $C_{43}H_{43}O_{17}$: 855.247, found 855.2486.

(2*R*,3*R*,4*S*,5*S*,6*S*)-2-(acetoxymethyl)-6-(4-((*E*)-5-(4-hydroxy-3-methoxyphenyl)-2-((*E*)-3-(4-hydroxy-3-methoxyphenyl)acryloyl)-3-oxopenta-1,4-dien-1-yl)-2-methoxyphenoxy)tetrahydro-*2H*-pyran-3,4,5-triyl triacetate (3a-Man)

This compound was obtained as orange solids (0.109 g, 0.130 mmol, 23%), m.p.: 203–204 °C. ^{1}H-NMR (400 MHz, $CDCl_3$) δ (ppm): 7.78–7.70 (m, 2H), 7.45 (d, *J* = 16.0 Hz, 1H), 7.14 (dd, *J* = 1.8, 8.2 Hz, 1H), 7.07–6.83 (m, 10H), 6.76 (d, *J* = 16.0 Hz, 1H), 6.11 (br. s., 2H), 5.69–5.66 (m, 1H), 5.35–5.23 (m, 1H), 5.16 (d, *J* = 1.4 Hz, 1H), 5.10 (dd, *J* = 3.2, 9.6 Hz, 1H), 4.28 (dd, *J* = 6.0, 12.4 Hz, 1H), 4.14–4.08 (m, 2H), 3.89 (s, 3H), 3.86 (s, 3H), 3.74 (s, 3H), 2.19 (s, 3H), 2.04 (s, 3H), 2.00 (s, 3H), 2.00 (s, 3H). ^{13}C-NMR (100 MHz, $CDCl_3$) δ (ppm): 198.4, 186.9, 170.7, 170.4, 170.1, 169.7, 150.4, 149.1, 148.7, 147.7, 147.3, 147.0, 146.9, 145.7, 140.2, 139.9, 129.8, 127.3, 126.5, 125.1, 124.2, 123.9, 123.8, 119.7, 119.0, 115.0, 115.0, 114.3, 110.5, 110.0, 97.3, 72.7, 70.8, 68.6, 66.0, 62.5, 56.1, 56.1, 56.0, 20.9, 20.8, 20.8, 20.7. HRMS (ESI-TOF) *m/z*: [M + H]$^+$ calculated for $C_{43}H_{43}O_{17}$: 833.2651, found 833.2651.

(2*R*,3*S*,4*S*,5*R*,6*S*)-2-(acetoxymethyl)-6-(3-((*E*)-5-(4-hydroxy-3-methoxyphenyl)-2-((*E*)-3-(4-hydroxy-3-methoxyphenyl)acryloyl)-3-oxopenta-1,4-dien-1-yl)phenoxy)tetrahydro-2*H*-pyran-3,4,5-triyl triacetate (4a-Gal)

This compound was obtained as orange solids (0.332 g, 0.414 mmol, 73%), m.p.: 200–201 °C. ^{1}H-NMR (400 MHz, $CDCl_3$) δ (ppm): 7.77 (s, 1H), 7.74 (d, *J* = 15.6 Hz, 1H), 7.43 (d, *J* = 16.0 Hz, 1H), 7.23 (d, *J* = 8.2 Hz, 1H), 7.20–7.11 (m, 3H), 7.02–6.84 (m, 8H), 6.76 (d, *J* = 16.0 Hz, 1H), 6.23 (d, *J* = 6.9 Hz, 2H), 5.47–5.40 (m, 2H), 5.06 (dd, *J* = 3.2, 10.5 Hz, 1H), 4.94 (d, *J* = 8.2 Hz, 1H), 4.20 (dq, *J* = 6.9, 11.1 Hz, 1H), 4.10 (q, *J* = 7.3 Hz, 3H), 3.89 (s, 3H), 3.87 (s, 3H), 2.03 (s, 3H), 2.02 (s, 3H), 2.02 (s, 3H), 1.99 (s, 3H). ^{13}C-NMR (100 MHz, $CDCl_3$) δ (ppm): 197.8, 186.9, 170.5, 170.3, 170.2, 169.5, 157.1, 149.2, 148.8, 147.8, 147.0, 146.0, 141.7, 139.6, 135.3, 130.1, 127.2, 126.5, 125.3, 125.1, 124.3, 123.9, 119.6, 119.3, 117.8, 115.0, 110.5, 110.1, 99.7, 71.2, 70.8, 68.6, 66.9, 61.2, 60.5, 56.1, 56.0, 21.1, 20.8, 20.7, 20.7. HRMS (ESI-TOF) *m/z*: [M + Na]$^+$ calculated for $C_{42}H_{42}O_{16}$: 825.2365, found 825.2362.

(2*R*,3*R*,4*S*,5*R*,6*S*)-2-(acetoxymethyl)-6-(3-((*E*)-5-(4-hydroxy-3-methoxyphenyl)-2-((*E*)-3-(4-hydroxy-3-methoxyphenyl)acryloyl)-3-oxopenta-1,4-dien-1-yl)phenoxy)tetrahydro-2*H*-pyran-3,4,5-triyl triacetate (4a-Glc)

This compound was obtained as orange solids (0.360 g, 0.448 mmol, 79%), m.p.: 201–203 °C. ^{1}H-NMR (400 MHz, $CDCl_3$) δ (ppm): 7.79 (s, 1H), 7.76 (d, *J* = 15.1 Hz, 1H), 7.44 (d, *J* = 16.0 Hz, 1H), 7.25–7.12 (m, 3H), 7.07–6.84 (m, 5H),

6.77 (d, *J* = 16.0 Hz, 1H), 6.01 (br. s., 2H), 5.26–5.22 (m, 2H), 5.21–5.14 (m, 1H), 5.00–4.96 (m, 1H), 4.26 (t, *J* = 3.2 Hz, 2H), 4.12 (q, *J* = 6.9 Hz, 2H), 3.93 (s, 3H), 3.91 (s, 3H), 2.06 (s, 3H), 2.05 (s, 3H), 2.04 (s, 3H), 2.03 (s, 3H). ^{13}C-NMR (100 MHz, $CDCl_3$) δ (ppm): 197.9, 186.8, 170.8, 170.4, 169.5, 169.4, 157.0, 149.2, 148.8, 147.9, 147.0, 146.9, 146.1, 141.7, 139.7, 135.3, 130.1, 127.2, 126.5, 125.4, 125.2, 124.3, 123.9, 119.6, 119.4, 117.2, 115.0, 110.5, 110.0, 99.0, 72.8, 72.2, 71.0, 68.1, 61.7, 56.2, 56.1, 20.8, 20.7, 20.7, 20.7. HRMS (ESI-TOF) *m/z*: $[M + Na]^+$ calculated for $C_{42}H_{42}O_{16}$: 825.2365, found 825.2373.

(3a*S*,5*R*,6*R*,7*S*,7a*S*)-5-(acetoxymethyl)-2-(3-((*E*)-5-(4-hydroxy-3-methoxyphenyl)-2-((*E*)-3-(4-hydroxy-3-methoxyphenyl)acryloyl)-3-oxopenta-1,4-dien-1-yl)phenoxy)-2-methyltetrahydro-5*H*-[1,3]dioxolo[4,5-b]pyran-6,7-diyl diacetate (4a-Man)
This compound was obtained as orange solids (0.118 g, 0.147 mmol, 26%), m.p.: 192–193 °C. ^{1}H-NMR (400 MHz, $CDCl_3$) δ (ppm): 7.76 (t, *J* = 7.8 Hz, 2H), 7.42 (d, *J* = 16.5 Hz, 1H), 7.25 (s, 1H), 7.24–7.20 (m, 1H), 7.16 (dd, *J* = 1.8, 8.2 Hz, 1H), 7.09–6.85 (m, 9H), 6.78 (d, *J* = 16.0 Hz, 1H), 5.96 (d, *J* = 6.0 Hz, 2H), 5.24 (t, *J* = 9.6 Hz, 1H), 5.21 (d, *J* = 2.3 Hz, 1H), 4.93 (dd, *J* = 3.9, 9.9 Hz, 1H), 4.23–4.16 (m, 1H), 4.16–4.08 (m, 3H), 4.05 (dd, *J* = 2.5, 3.9 Hz, 1H), 3.93 (s, 3H), 3.90 (s, 3H), 2.13 (s, 3H), 2.07 (s, 3H), 2.04 (s, 3H), 1.61 (s, 3H). ^{13}C-NMR (100 MHz, $CDCl_3$) δ (ppm): 197.8, 186.9, 170.8, 170.2, 169.6, 152.7, 149.0, 148.7, 147.7, 146.9, 146.8, 145.9, 141.4, 139.6, 134.8, 129.6, 127.3, 126.9, 126.7, 125.5, 125.0, 124.6, 124.5, 124.2, 123.9, 119.7, 114.9, 110.4, 110.0, 97.3, 76.0, 71.5, 65.5, 62.5, 60.5, 56.2, 56.1, 24.6, 20.8, 20.8, 20.8. HRMS (ESI) *m/z*: $[M + Na]^+$ calculated for $C_{42}H_{42}O_{16}$: 825.2365, found 825.2375.

1.4 Deacetylated glycoside derivatives

1,7-bis(4-hydroxy-3-methoxyphenyl)-4-(4-(((2*S*,3*R*,4*S*,5*R*,6*R*)-3,4,5-trihydroxy-6-(hydroxymethyl)tetrahydro-2*H*-pyran-2-yl)oxy)benzylidene)hepta-1,6-diene-3,5-dione (2b-Gal)
This compound was obtained as dark brown solids (0.0892 g, 0.141 mmol, 89%), m.p.: 221–223 °C. ^{1}H-NMR (400 MHz, CD_3OD) δ (ppm): 7.85 (s, 1H), 7.68 (d, *J* = 15.1 Hz, 1H), 7.50 (d, *J* = 8.7 Hz, 2H), 7.46 (d, *J* = 16.0 Hz, 1H), 7.19–7.07 (m, 3H), 7.04 (d, *J* = 8.7 Hz, 2H), 7.03–7.00 (m, 1H), 6.95 (dd, *J* = 1.8, 8.2 Hz, 1H), 6.71 (s, 1H), 6.68 (d, *J* = 6.9 Hz, 1H), 6.60 (d, *J* = 8.2 Hz, 1H), 3.85 (d, *J* = 2.3 Hz, 1H), 3.83 (s, 3H), 3.77 (s, 3H), 3.70 (d, *J* = 5.5 Hz, 1H), 3.65 (d, *J* = 5.0 Hz, 2H), 3.62 (d, *J* = 5.5 Hz, 1H), 3.41 (d, *J* = 2.8 Hz, 2H). ^{13}C-NMR (100 MHz, CD_3OD) δ (ppm): 199.4, 188.3, 159.4, 149.8, 149.1, 147.7, 146.7, 146.6, 146.5, 145.9, 139.2, 139.1, 132.0, 128.3, 127.9, 126.9, 126.3, 125.1, 124.3, 120.2, 116.7, 114.4, 114.3, 110.4, 110.0, 100.3, 76.9, 76.5, 73.5, 69.9, 61.0, 54.9, 54.7. HRMS (ESI-TOF) *m/z*: $[M + Na]^+$ calculated for $C_{34}H_{34}O_{12}$: 657.1942, found 657.1951.

1,7-bis(4-hydroxy-3-methoxyphenyl)-4-(4-(((2*S*,3*R*,4*S*,5*S*,6*R*)-3,4,5-trihydroxy-6-(hydroxymethyl)tetrahydro-2*H*-pyran-2-yl)oxy)benzylidene)hepta-1,6-diene-3,5-dione (2b-Glc)
This compound was obtained as dark brown solids (0.0862 g, 0.136 mmol, 86%), m.p.: 221–223 °C. ^{1}H-NMR (400 MHz, CD_3OD) δ (ppm): 7.88 (s, 1H), 7.68 (d, *J* = 15.6 Hz, 1H), 7.53–7.43 (m, 4H), 7.17–7.23 (m, 2H), 7.13–7.09 (m, 1H), 7.07–7.03 (m, 3H), 6.96 (dd, *J* = 1.6, 8.5 Hz, 1H), 6.74–6.69 (m, 2H), 6.64 (d, *J* = 8.2 Hz, 1H), 3.86 (d, *J* = 3.2 Hz, 1H), 3.84 (s, 3H), 3.78 (s, 3H), 3.77–3.71 (m, 2H), 3.70 (d, *J* = 3.2 Hz, 2H), 3.67–3.64 (m, 1H), 3.54 (dd, *J* = 3.2, 9.6 Hz, 1H). ^{13}C-NMR (100 MHz, CD_3OD) δ (ppm): 199.4, 188.4, 159.5, 149.5, 148.7, 147.8, 146.2, 146.1, 145.1, 139.7, 139.5, 132.0, 127.7, 127.2, 126.1, 125.0, 124.1, 123.7, 119.5, 116.5, 114.1, 114.1, 110.6, 110.2, 101.0, 75.7, 73.4, 70.8, 68.9, 61.1, 55.0, 54.8. HRMS (ESI-TOF) *m/z*: $[M + Na]^+$ calculated for $C_{34}H_{34}O_{12}$: 657.1942, found 657.1946.

1,7-bis(4-hydroxy-3-methoxyphenyl)-4-(4-(((2*S*,3*S*,4*S*,5*S*,6*R*)-3,4,5-trihydroxy-6-(hydroxymethyl)tetrahydro-2*H*-pyran-2-yl)oxy)benzylidene)hepta-1,6-diene-3,5-dione (2b-Man)
This compound was obtained as dark brown solids (0.0762 g, 0.120 mmol, 76%), m.p.: 222–223 °C. ^{1}H-NMR (400 MHz, CD_3OD) δ (ppm): 7.90 (s, 1H), 7.68 (s, 1H), 7.72 (s, 1H), 7.54–7.46 (m, 5H), 7.25–7.20 (m, 3H), 7.16–7.12 (m, 2H), 7.10–7.05 (m, 5H), 6.98 (dd, *J* = 1.6, 8.5 Hz, 2H), 6.77 (s, 2H), 6.74 (d, *J* = 8.7 Hz, 2H), 6.67 (d, *J* = 8.2 Hz, 2H), 4.03–4.00 (dd, *J* = 1.0, 8.2 Hz, 1H), 3.84 (d, *J* = 6.9 Hz, 1H), 3.80 (s, 3H), 3.78 (s, 4H), 3.76–3.70 (m, 2H), 3.70–3.65 (m, 1H), 3.62 (t, *J* = 1.0 Hz, 1H), 3.52 (dd, *J* = 2.3, 9.2 Hz, 1H). ^{13}C-NMR (100 MHz, CD_3OD) δ (ppm): 196.4, 188.8, 160.3, 150.2, 149.8, 148.1, 146.8, 146.5, 145.1, 140.6, 137.8, 132.7, 129.6, 126.9, 126.2, 125.2, 124.9, 124.3, 123.9, 120.5, 120.1, 118.3, 115.3, 110.9, 110.8, 99.9, 72.7, 70.9, 69.2, 61.4, 60.4, 55.2, 55.1. HRMS (ESI-TOF) *m/z*: $[M + Na]^+$ calculated for $C_{34}H_{34}O_{12}$: 657.1942, found 657.1928.

1,7-bis(4-hydroxy-3-methoxyphenyl)-4-(3-methoxy-4-(((2*S*,3*R*,4*S*,5*R*,6*R*)-3,4,5-trihydroxy-6-(hydroxymethyl)tetrahydro-2*H*-pyran-2-yl)oxy)benzylidene)hepta-1,6-diene-3,5-dione (3b-Gal)

This compound was obtained as dark brown solids (0.104 g, 0.156 mmol, 99%), m.p.: 219–221 °C. ^{1}H-NMR (400 MHz, CD_3OD) δ (ppm): 7.94 (s, 1H), 7.66 (d, *J* = 15.6 Hz, 1H), 7.49 (d, *J* = 16.0 Hz, 1H), 7.34 (d, *J* = 15.1 Hz, 1H), 7.29–7.27 (m, 1H), 7.18–7.12 (m, 5H), 7.02 (dd, *J* = 1.8, 8.2 Hz, 1H), 6.85–6.79 (m, 2H), 6.75 (d, *J* = 8.2 Hz, 1H), 3.89 (s, 3H), 3.85 (dd, *J* = 4.1, 7.8 Hz, 2H), 3.82 (s, 4H), 3.79 (d, *J* = 1.8 Hz, 1H), 3.76 (s, 3H), 3.71–3.68 (m, 2H), 3.66–3.61 (m, 1H), 3.55–3.51 (m, 1H). ^{13}C-NMR (100 MHz, CD_3OD) δ (ppm): 188.7, 168.7, 150.4, 149.9, 149.3, 148.9, 148.5, 148.1, 148.1, 145.5, 140.5, 139.7, 128.0, 126.8, 126.1, 124.5, 124.1, 123.9, 123.7, 118.1, 115.8, 115.3, 115.3, 113.8, 111.0, 110.8, 101.3, 75.8, 73.4, 70.7, 68.8, 61.0, 55.3, 55.2, 55.1. HRMS (ESI-TOF) *m/z*: $[M + Na]^+$ calculated for $C_{35}H_{36}O_{13}$: 687.2048, found 687.2046.

1,7-bis(4-hydroxy-3-methoxyphenyl)-4-(3-methoxy-4-(((2*S*,3*R*,4*S*,5*S*,6*R*)-3,4,5-trihydroxy-6-(hydroxymethyl)tetrahydro-2*H*-pyran-2-yl)oxy)benzylidene)hepta-1,6-diene-3,5-dione (3b-Glc)

This compound was obtained as dark brown solids (0.0798 g, 0.120 mmol, 76%), m.p.: 219–220 °C. ^{1}H-NMR (400 MHz, CD_3OD) δ (ppm): 7.94 (s., 1H), 7.66 (d, *J* = 13.7 Hz, 1H), 7.49 (d, *J* = 15.6 Hz, 1H), 7.40–7.22 (m, 2H), 7.22–7.07 (m, 5H), 7.02 (d, *J* = 7.3 Hz, 1H), 6.85–6.73 (m, 4H), 3.89 (s, 3H), 3.87–3.84 (m, 2H), 3.82 (s, 3H), 3.80–3.78 (m, 1H), 3.76 (s, 3H), 3.71–3.67 (m, 2H), 3.66–3.61 (m, 1H), 3.53 (ddd, *J* = 0.9, 3.4, 9.9 Hz, 1H). ^{13}C-NMR (100 MHz, CD_3OD) δ (ppm): δ 199.6, 186.5, 150.5, 149.9, 149.3, 148.9, 148.5, 148.2, 148.1, 145.5, 140.2, 139.7, 130.2, 128.0, 126.8, 126.0, 124.1, 123.9, 123.7, 118.9, 118.1, 115.3, 115.3, 115.3, 113.8, 111.0, 110.8, 101.3, 75.8, 73.4, 70.7, 68.8, 61.0, 55.3, 55.2, 55.1. HRMS (ESI-TOF) *m/z*: $[M + Na]^+$ calculated for $C_{35}H_{36}O_{13}$: 687.2048, found 687.2041.

1,7-bis(4-hydroxy-3-methoxyphenyl)-4-(3-methoxy-4-(((2S,3S,4S,5S,6R)-3,4,5-trihydroxy-6-(hydroxymethyl)tetrahydro-2H-pyran-2-yl)oxy)benzylidene)hepta-1,6-diene-3,5-dione (3b-Man)

This compound was obtained as dark brown solids (0.0977 g, 0.147 mmol, 93%), m.p.: 220–221 °C. ^{1}H-NMR (400 MHz, CD_3OD) δ (ppm): 7.95 (s, 1H), 7.68 (d, *J* = 15.6 Hz, 1H), 7.51 (d, *J* = 16.0 Hz, 1H), 7.36 (d, *J* = 15.1 Hz, 1H), 7.31–7.28 (m, 1H), 7.20–7.13 (m, 6H), 7.04 (dd, *J* = 1.8, 8.2 Hz, 1H), 6.85 (d, *J* = 11.5 Hz, 1H), 6.82 (d, *J* = 3.7 Hz, 1H), 6.77 (d, *J* = 8.2 Hz, 1H), 4.02 (dd, *J* = 1.0, 8.2 Hz, 1H), 3.85 (d, *J* = 6.9 Hz, 1H), 3.80 (s, 3H), 3.79 (s, 4H), 3.73 (s, 3H), 3.72–3.68 (m, 2H), 3.66 (t, *J* = 1.0 Hz, 1H), 3.65–3.59 (m, 1H), 3.53 (dd, *J* = 2.3, 9.2 Hz, 1H). ^{13}C-NMR (100 MHz, CD_3OD) δ (ppm): 199.5, 188.6, 150.3, 149.8, 149.1, 148.5, 148.3, 148.1, 148.0, 145.5, 140.4, 139.8, 128.3, 126.8, 126.0, 124.4, 124.1, 123.9, 123.7, 118.1, 116.1, 115.3, 115.3, 113.7, 111.0, 110.8, 98.5, 77.2, 73.4, 70.9, 66.8, 61.1, 55.2, 55.1, 53.5. HRMS (ESI-TOF) *m/z*: $[M + Na]^+$ calculated for $C_{35}H_{36}O_{13}$: 687.2048, found 687.2032.

1,7-bis(4-hydroxy-3-methoxyphenyl)-4-(3-(((2*S*,3*R*,4*S*,5*R*,6*R*)-3,4,5-trihydroxy-6-(hydroxymethyl)tetrahydro-2*H*-pyran-2-yl)oxy)benzylidene)hepta-1,6-diene-3,5-dione (4b-Gal)

Dark brown solids (0.0732 g, 0.115 mmol, 73%), m.p.: 212–213 °C. ^{1}H-NMR (400 MHz, CD_3OD) δ (ppm): 7.95 (s., 1H), 7.67 (d, *J* = 14.2 Hz, 1H), 7.46 (d, *J* = 15.6 Hz, 1H), 7.18–7.29 (m, 2H), 7.17–7.04 (m, 4H), 7.03–6.91 (m, 3H), 6.82–6.72 (m, 3H), 3.88 (d, *J* = 2.3 Hz, 2H), 3.86 (s, 3H), 3.85–3.82 (m, 2H), 3.80 (s, 3H), 3.76–3.71 (m, 1H), 3.63 (t, *J* = 6.0 Hz, 1H), 3.52–3.48 (m, 1H). ^{13}C-NMR (100 MHz, CD_3OD) δ (ppm): 199.0, 188.8, 157.9, 150.4, 150.0, 148.1, 146.8, 145.9, 141.4, 140.3, 135.1, 129.6, 126.7, 126.0, 124.1, 123.9, 118.8, 118.1, 117.2, 115.3, 110.9, 110.8, 101.4, 75.5, 73.5, 70.8, 68.8, 60.9, 55.2, 55.1. HRMS (ESI-TOF) *m/z*: $[M + Na]^+$ calculated for $C_{34}H_{34}O_{12}$: 657.1942, found 657.1948.

1,7-bis(4-hydroxy-3-methoxyphenyl)-4-(3-(((2*S*,3*R*,4*S*,5*S*,6*R*)-3,4,5-trihydroxy-6-(hydroxymethyl)tetrahydro-2*H*-pyran-2-yl)oxy)benzylidene)hepta-1,6-diene-3,5-dione (4b-Glc)

Dark brown solids (0.0953 g, 0.150 mmol, 95%), m.p.: 212–214 °C. ^{1}H-NMR (400 MHz, CD_3OD) δ (ppm): 7.95 (s, 1H), 7.68 (d, *J* = 15.6 Hz, 1H), 7.45 (d, *J* = 16.0 Hz, 1H), 7.35–7.24 (m, 4H), 7.20–7.11 (m, 4H), 7.08–6.99 (m, 2H), 6.81 (d, *J* = 7.3 Hz, 2H), 6.76–6.73 (m, 2H), 3.88 (s, 3H), 3.87–3.85 (m, 2H), 3.85 (d, *J* = 2.8 Hz, 1H), 3.82 (s, 3H), 3.82–3.80 (m, 1H), 3.76–3.72 (m, 1H), 3.71 (d, *J* = 2.8 Hz, 1H), 3.68 (m, 1H). ^{13}C-NMR (100 MHz, CD_3OD) δ (ppm): 199.0, 188.8, 157.9, 150.4, 150.0, 148.8, 148.1, 147.7, 145.9, 141.4, 140.2, 135.1, 129.6, 126.8, 126.0, 125.4, 125.1, 124.0, 123.9, 119.5, 119.3, 118.1, 115.3, 110.9, 110.8, 100.9, 73.4, 72.9, 71.8, 69.7, 61.4, 55.2, 55.1. HRMS (ESI-TOF) *m/z*: $[M + Na]^+$ calculated for $C_{34}H_{34}O_{12}$: 657.1942, found 657.1949.

4-(3-(((3a*S*,5*R*,6*S*,7*S*,7a*S*)-6,7-dihydroxy-5-(hydroxymethyl)-2-methyltetrahydro-5*H*-[1,3]dioxolo[4,5-b]pyran-2-yl)oxy)benzylidene)-1,7-bis(4-hydroxy-3-methoxyphenyl)hepta-1,6-diene-3,5-dione (4b-Man)

Dark brown solids (0.0823 g, 0.122 mmol, 77%), m.p.: 207–208 °C. ^{1}H-NMR (400 MHz, CD_3OD) δ (ppm): 7.89 (s, 1H), 7.67 (d, *J* = 15.1 Hz, 1H), 7.44 (d, *J* = 16.0 Hz, 1H), 7.33–7.27 (m, 2H), 7.17–7.09 (m, 3H), 7.02–6.93 (m, 2H), 6.83–6.77 (m, 2H), 6.77–6.72 (m, 2H), 4.33 (dd, *J* = 2.3, 6.0 Hz, 1H), 3.91 (d, *J* = 7.3 Hz, 1H), 3.88 (s, 3H), 3.81 (s, 3H), 3.80–3.75 (m, 1H), 3.70–3.62 (m, 2H), 3.62–3.55 (m, 1H), 3.55–3.52 (m, 1H), 1.55 (s, 3H). ^{13}C-NMR (100 MHz, CD_3OD) δ (ppm): 199.0, 188.8, 157.5, 150.3, 148.6, 148.1, 147.4, 146.6, 146.6, 145.8, 140.8, 139.7, 135.0, 129.5, 126.8, 126.2,

126.1, 124.9, 124.2, 123.9, 123.8, 123.0, 122.7, 121.6, 115.3, 110.9, 110.7, 100.0, 76.6, 70.9, 65.3, 63.8, 61.5, 55.2, 55.1, 22.8. HRMS (ESI-TOF) *m/z*: [M + Na]+ calculated for $C_{34}H_{34}O_{12}$: 657.1942, found 657.1929.

2. NMR spectra of the synthesised sugar-conjugated derivatives

2.1 Sugar-conjugated benzaldehyde intermediates

(2*R*,3*S*,4*S*,5*R*,6*S*)-2-(acetoxymethyl)-6-(4-formylphenoxy)tetrahydro-2*H*-pyran-3,4,5-triyl triacetate (**9a**)

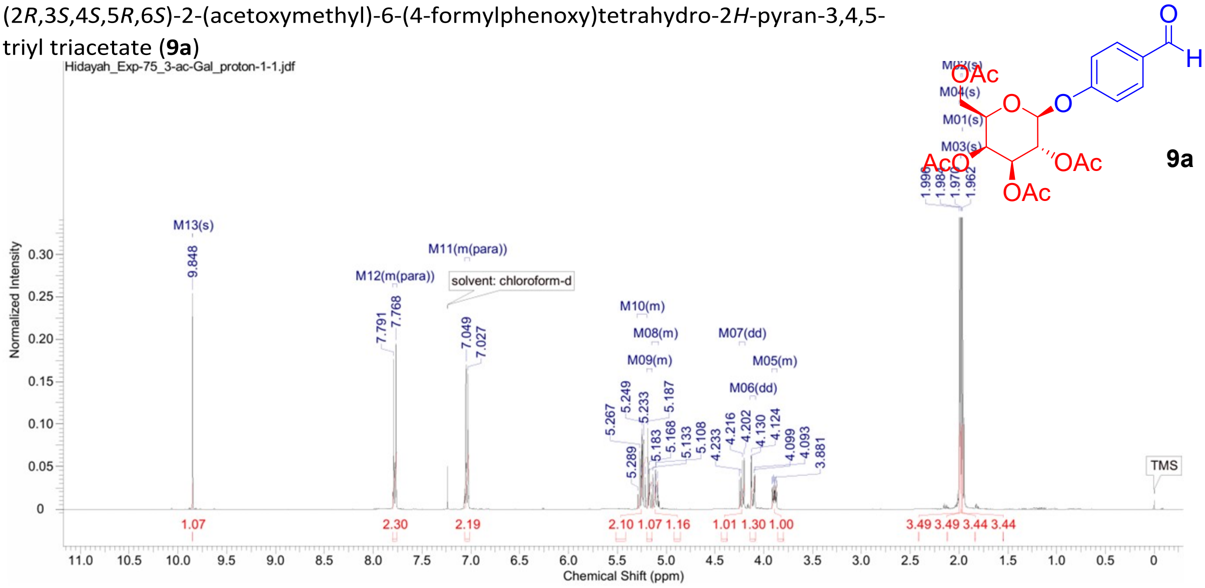

(2*R*,3*R*,4*S*,5*R*,6*S*)-2-(acetoxymethyl)-6-(4-formylphenoxy)tetrahydro-2*H*-pyran-3,4,5-triyl triacetate (**9b**)

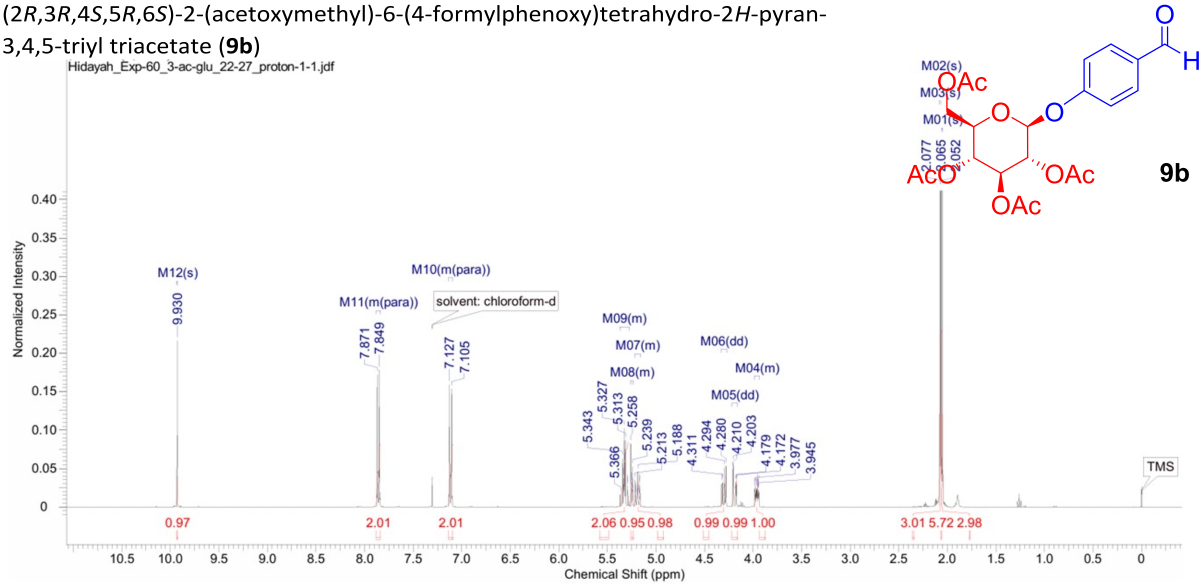

(2*R*,3*R*,4*S*,5*S*,6*S*)-2-(acetoxymethyl)-6-(4-formylphenoxy)tetrahydro-2*H*-pyran-3,4,5-triyl triacetate (**9c**)

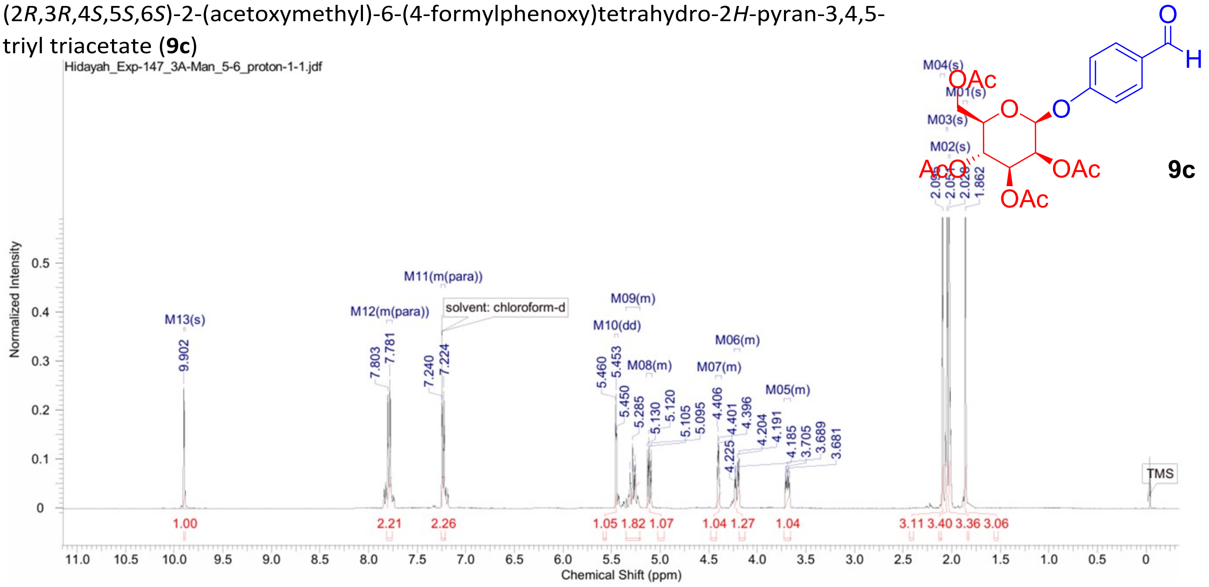

(2*R*,3*S*,4*S*,5*R*,6*S*)-2-(acetoxymethyl)-6-(4-formyl-2-methoxyphenoxy)tetrahydro-2*H*-pyran-3,4,5-triyl triacetate (**10a**)

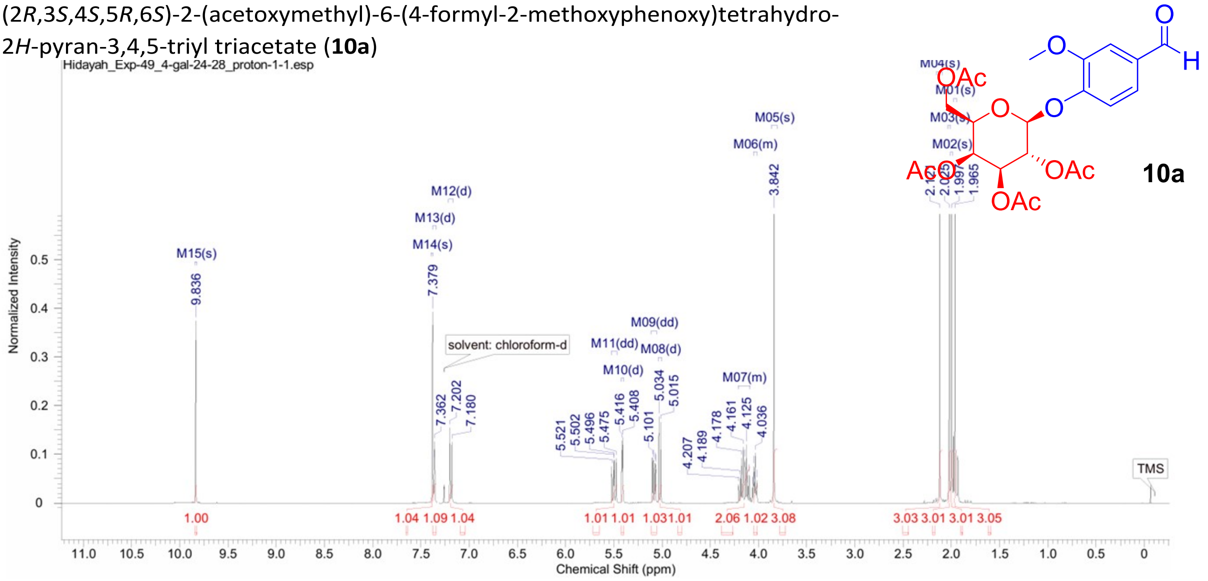

(2*R*,3*R*,4*S*,5*R*,6*S*)-2-(acetoxymethyl)-6-(4-formyl-2-methoxyphenoxy)tetrahydro-2*H*-pyran-3,4,5-triyl triacetate (**10b**)

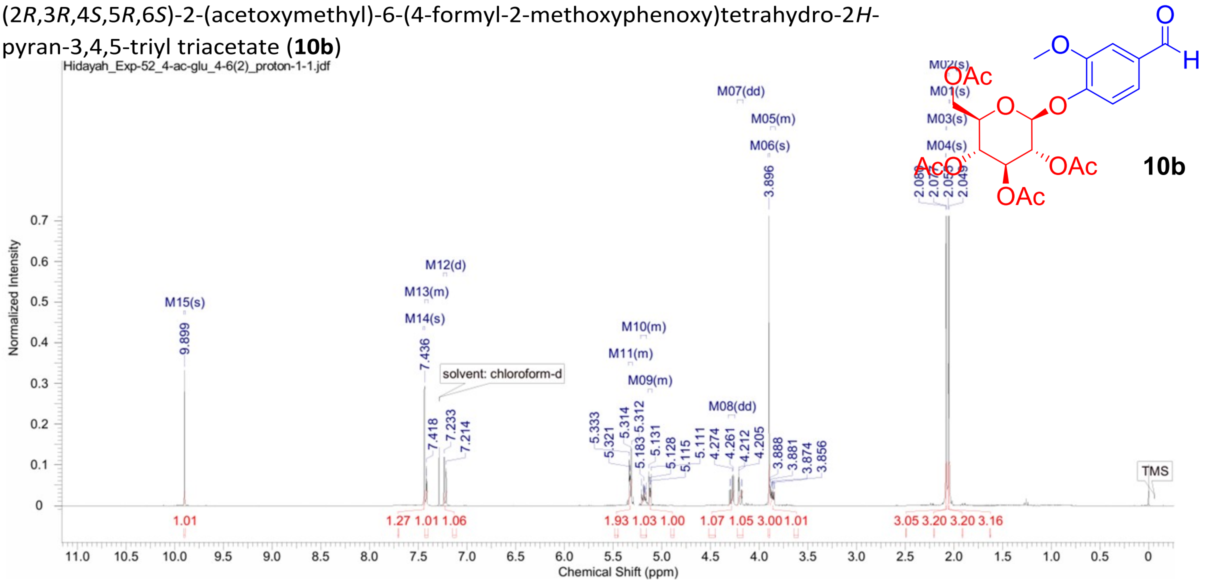

(2*R*,3*R*,4*S*,5*S*,6*S*)-2-(acetoxymethyl)-6-(4-formyl-2-methoxyphenoxy)tetrahydro-2*H*-pyran-3,4,5-triyl triacetate (**10c**)

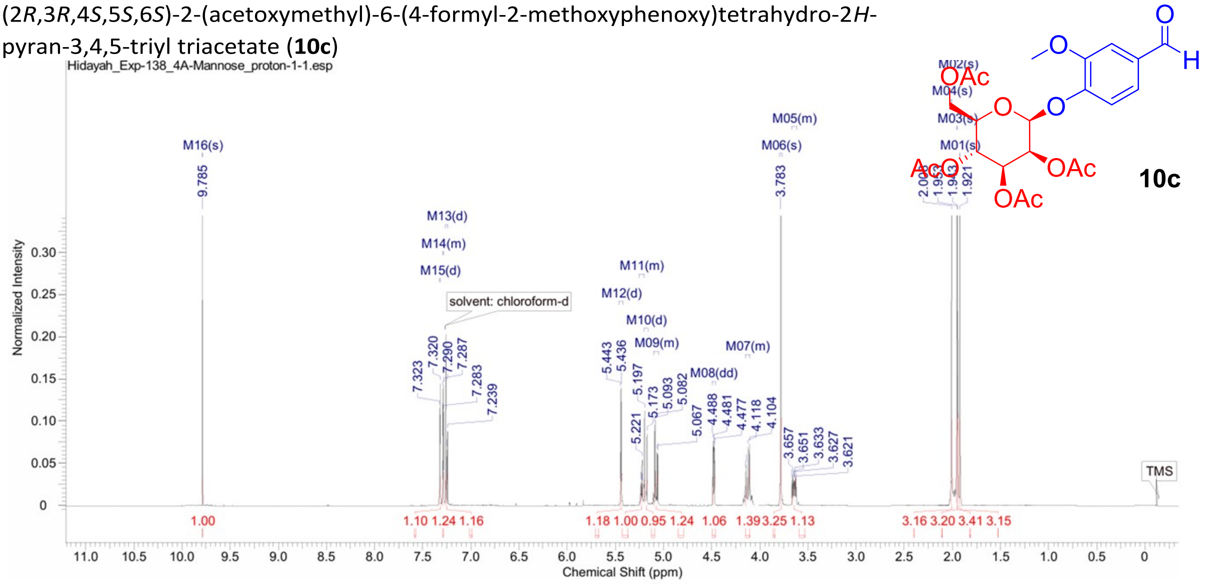

(2*R*,3*S*,4*S*,5*R*,6*S*)-2-(acetoxymethyl)-6-(3-formylphenoxy)tetrahydro-2*H*-pyran-3,4,5-triyl triacetate (**11a**)

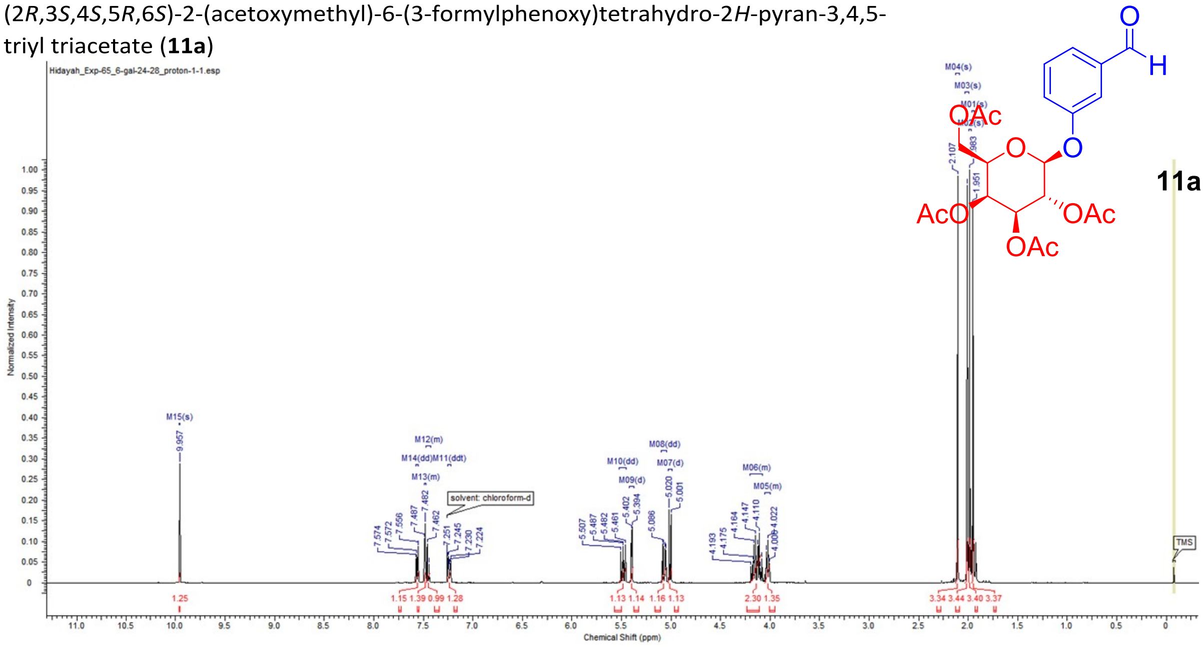

11a

(2*R*,3*R*,4*S*,5*R*,6*S*)-2-(acetoxymethyl)-6-(3-formylphenoxy)tetrahydro-2*H*-pyran-3,4,5-triyl triacetate (**11b**)

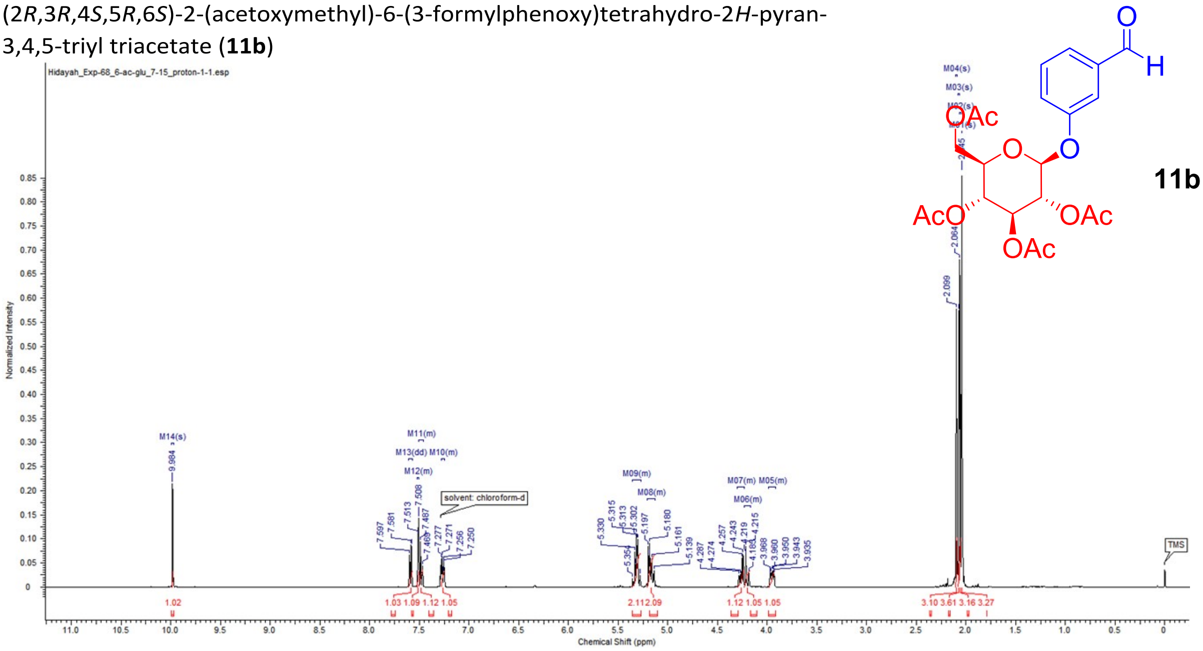

11b

(3a*S*,5*R*,6*R*,7*S*,7a*S*)-5-(acetoxymethyl)-2-(3-formylphenoxy)-2-methyltetrahydro-5*H*-[1,3]dioxolo[4,5-b]pyran-6,7-diyl diacetate (**11c**)

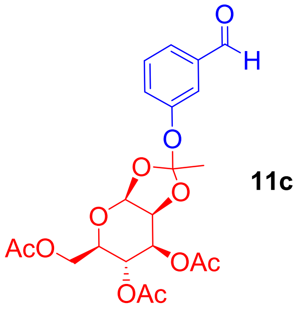

11c

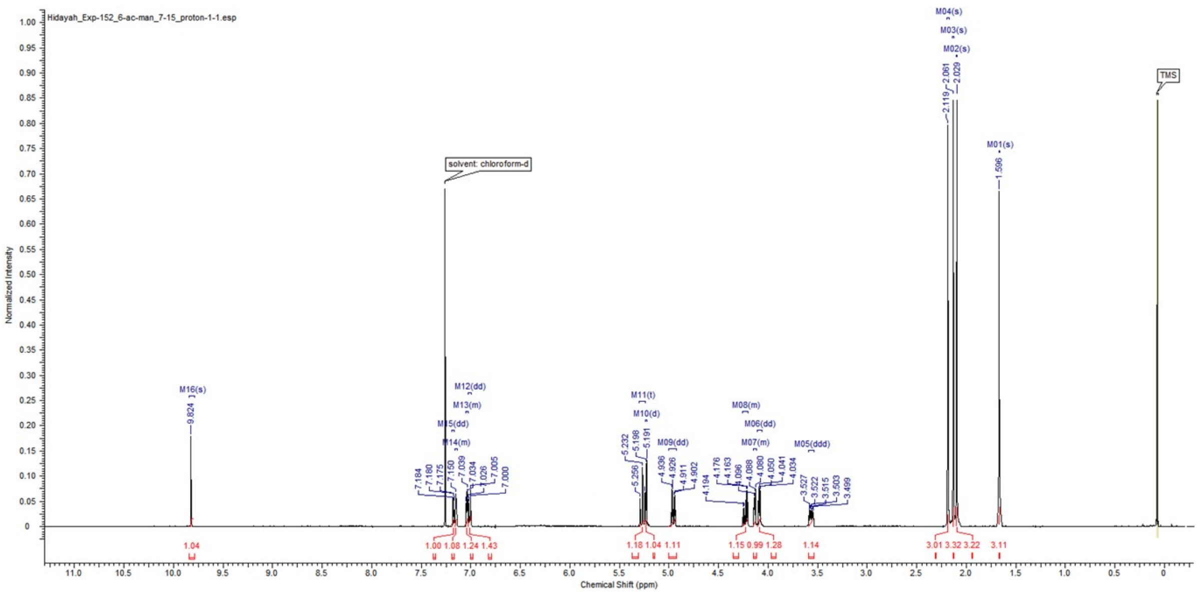

Hidayah_Exp-152_6-ac-man_7-15_proton-1-1.esp
solvent: chloroform-d
TMS
M16(s)
M15(dd)
M14(m)
M13(m)
M12(dd)
M11(t)
M10(d)
M09(dd)
M08(m)
M07(m)
M06(dd)
M05(ddd)
M04(s)
M03(s)
M02(s)
M01(s)
Normalized Intensity
Chemical Shift (ppm)

2.2 Acetylated glycoside derivatives

(2*R*,3*S*,4*S*,5*R*,6*S*)-2-(acetoxymethyl)-6-(4-((*E*)-5-(4-hydroxy-3-methoxyphenyl)-2-((*E*)-3-(4-hydroxy-3-methoxyphenyl)acryloyl)-3-oxopenta-1,4-dien-1-yl)phenoxy)tetrahydro-2*H*-pyran-3,4,5-triyl triacetate (**2a-Gal**)

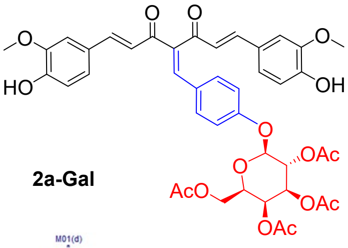

2a-Gal

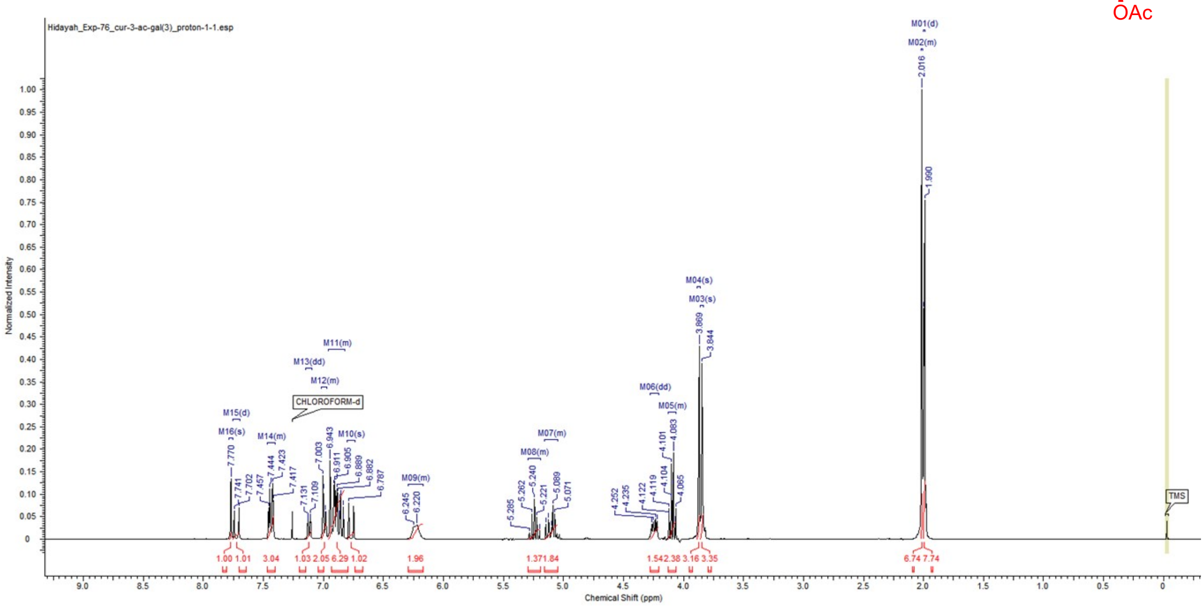

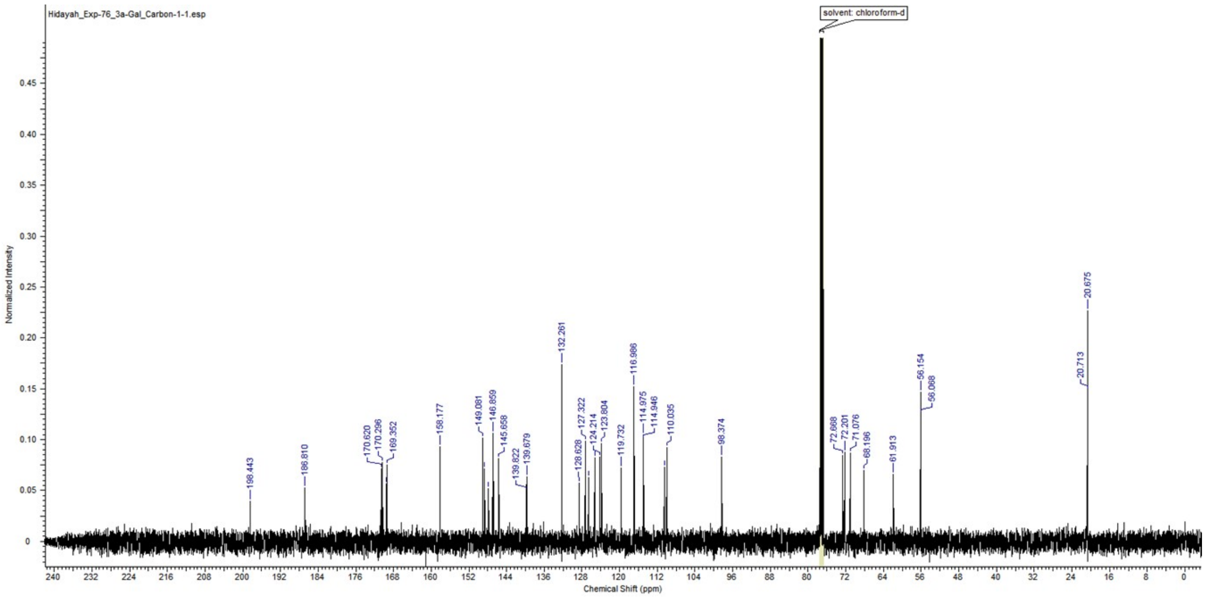

(2*R*,3*R*,4*S*,5*R*,6*S*)-2-(acetoxymethyl)-6-(4-((*E*)-5-(4-hydroxy-3-methoxyphenyl)-2-((*E*)-3-(4-hydroxy-3-methoxyphenyl)acryloyl)-3-oxopenta-1,4-dien-1-yl)phenoxy)tetrahydro-2*H*-pyran-3,4,5-triyl triacetate (**2a-Glc**)

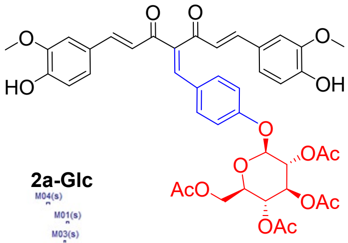

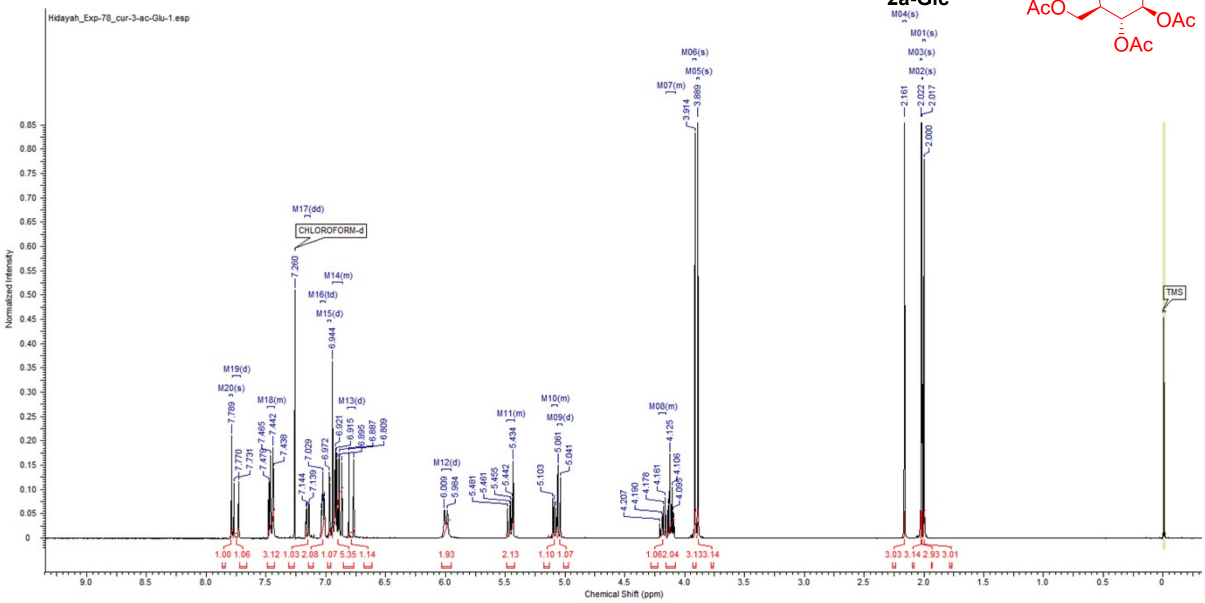

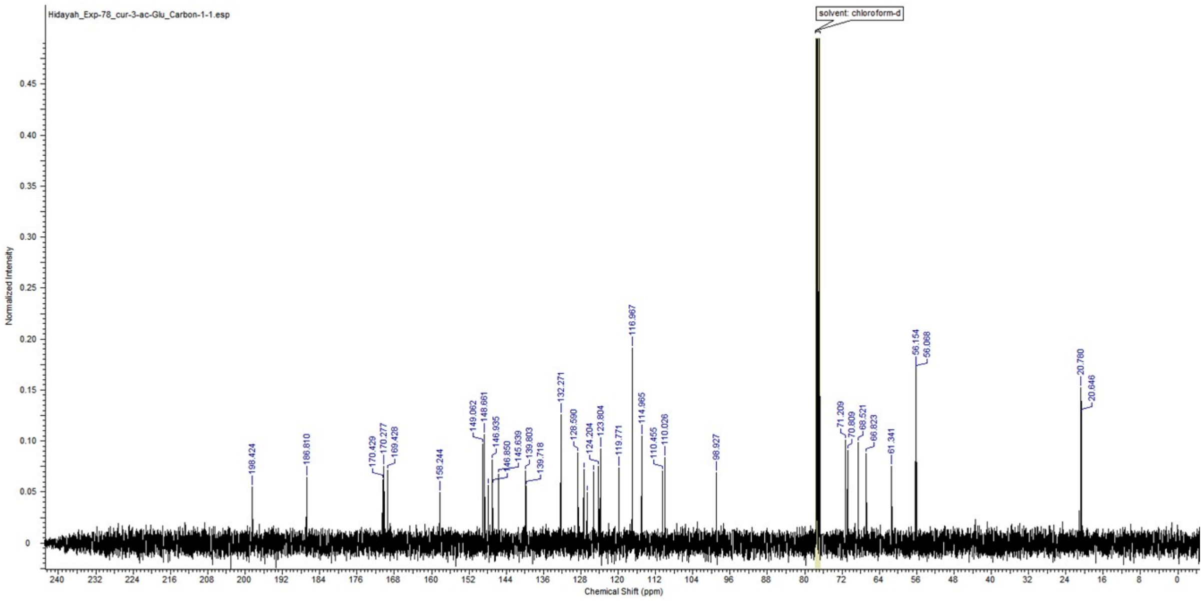

(2*R*,3*R*,4*S*,5*S*,6*S*)-2-(acetoxymethyl)-6-(4-((*E*)-5-(4-hydroxy-3-methoxyphenyl)-2-((*E*)-3-(4-hydroxy-3-methoxyphenyl)acryloyl)-3-oxopenta-1,4-dien-1-yl)phenoxy)tetrahydro-2*H*-pyran-3,4,5-triyl triacetate (**2a-Man**)

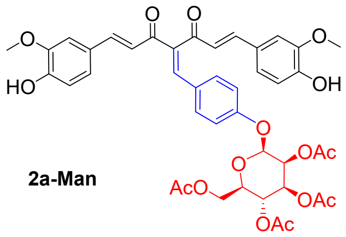

2a-Man

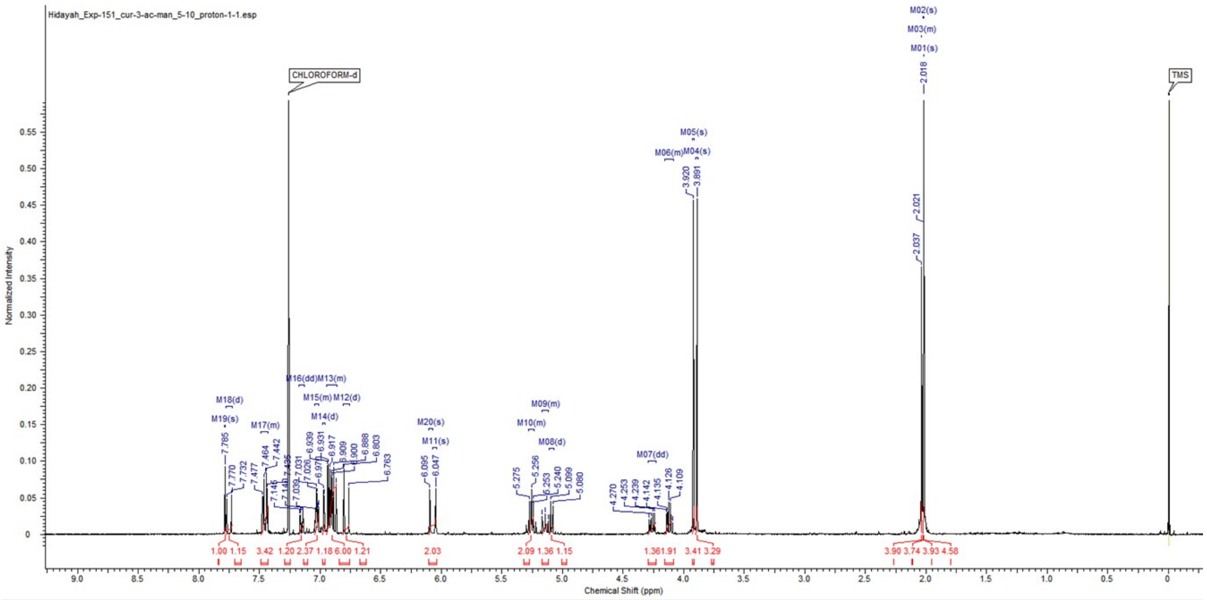

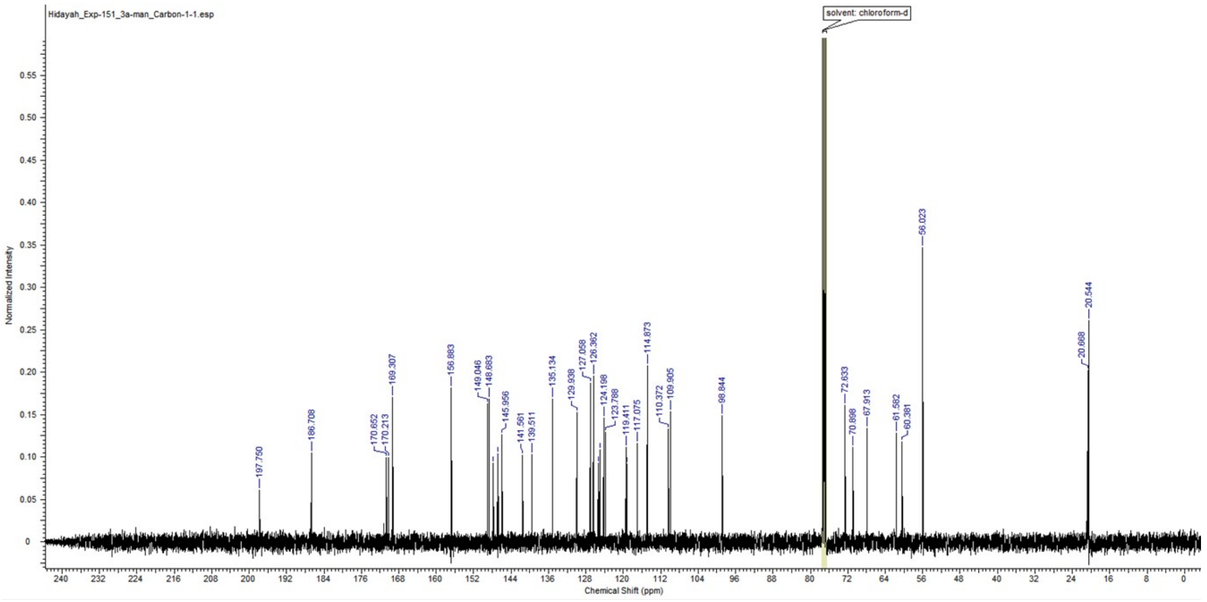

(2*R*,3*S*,4*S*,5*R*,6*S*)-2-(acetoxymethyl)-6-(4-((*E*)-5-(4-hydroxy-3-methoxyphenyl)-2-((*E*)-3-(4-hydroxy-3-methoxyphenyl)acryloyl)-3-oxopenta-1,4-dien-1-yl)-2-methoxyphenoxy)tetrahydro-2*H*-pyran-3,4,5-triyl triacetate (**3a-Gal**)

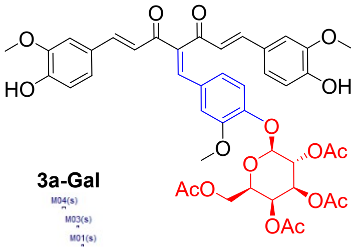

3a-Gal

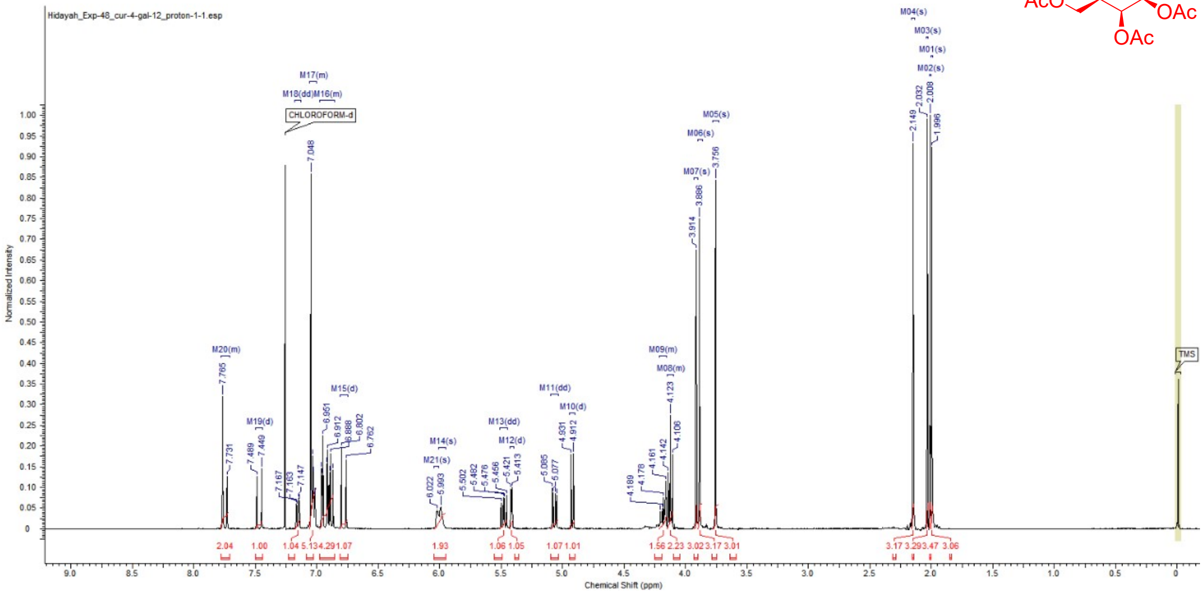

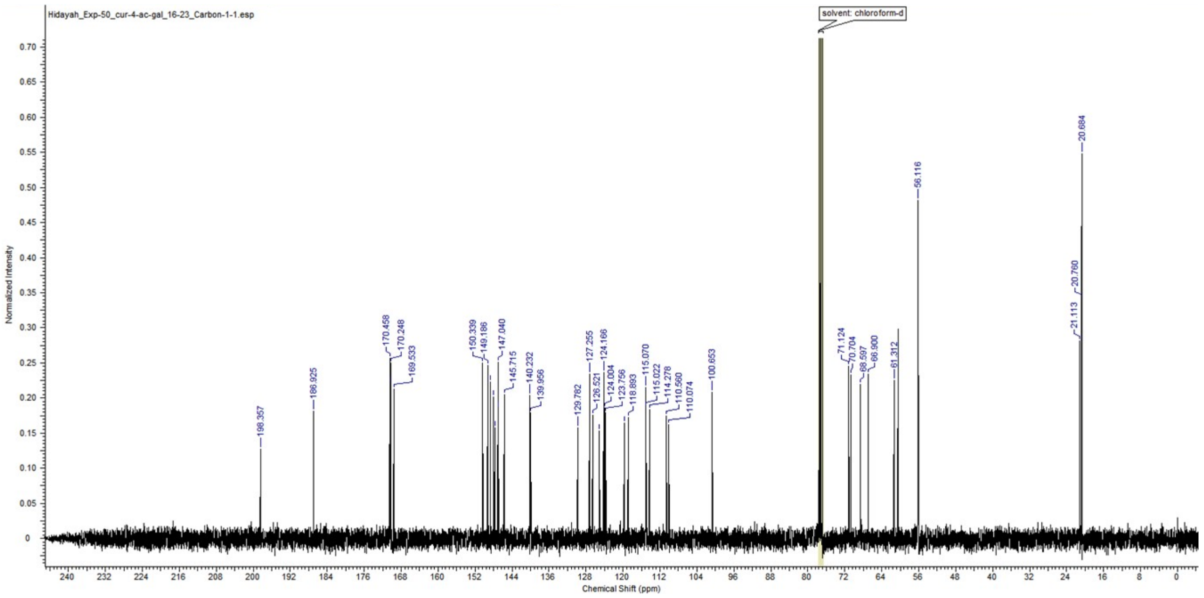

(2*R*,3*R*,4*S*,5*R*,6*S*)-2-(acetoxymethyl)-6-(4-((*E*)-5-(4-hydroxy-3-methoxyphenyl)-2-((*E*)-3-(4-hydroxy-3-methoxyphenyl)acryloyl)-3-oxopenta-1,4-dien-1-yl)-2-methoxyphenoxy)tetrahydro-2*H*-pyran-3,4,5-triyl triacetate (**3a-Glc**)

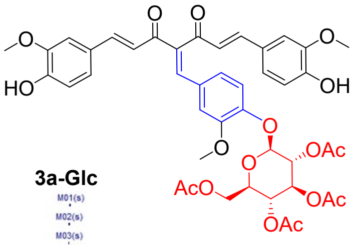

3a-Glc

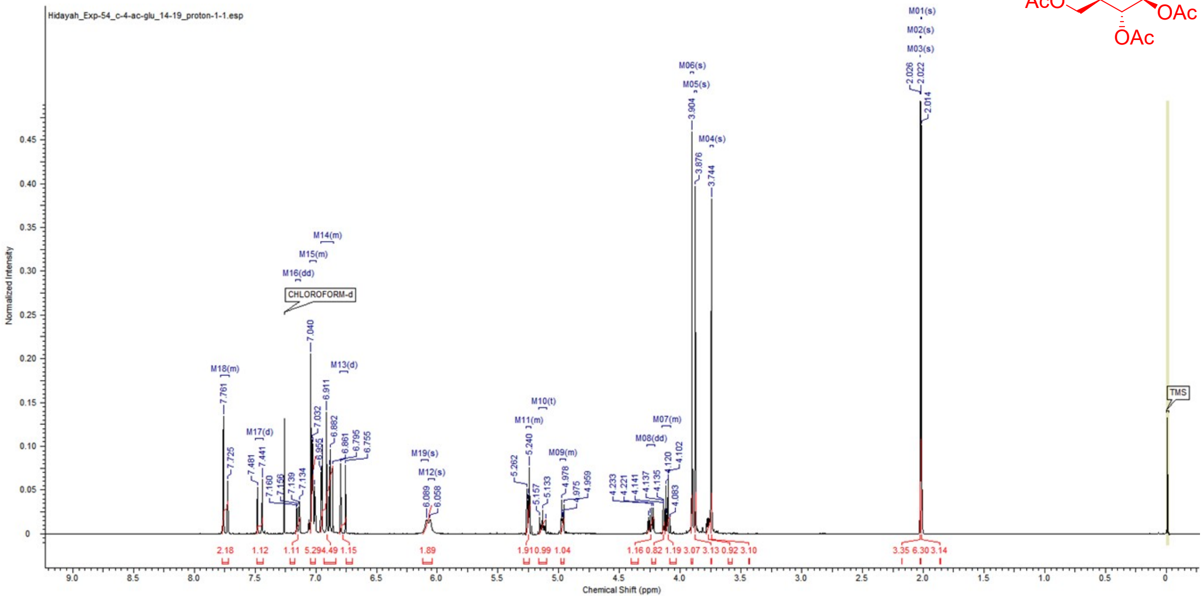

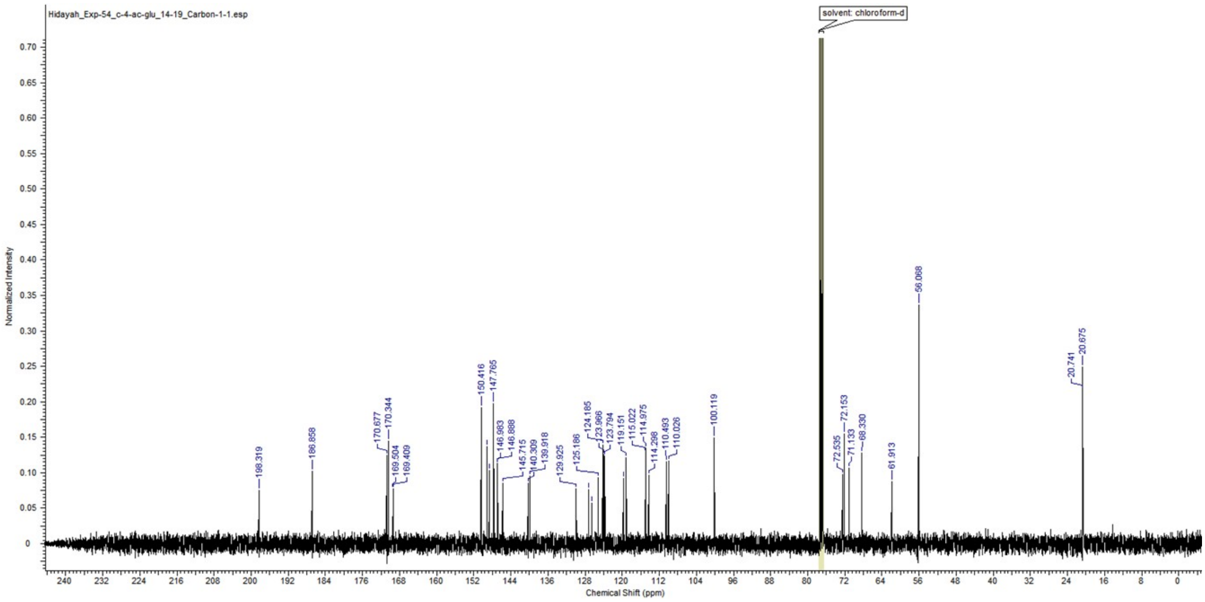

(2*R*,3*R*,4*S*,5*S*,6*S*)-2-(acetoxymethyl)-6-(4-((*E*)-5-(4-hydroxy-3-methoxyphenyl)-2-((*E*)-3-(4-hydroxy-3-methoxyphenyl)acryloyl)-3-oxopenta-1,4-dien-1-yl)-2-methoxyphenoxy)tetrahydro-2*H*-pyran-3,4,5-triyl triacetate (**3a-Man**)

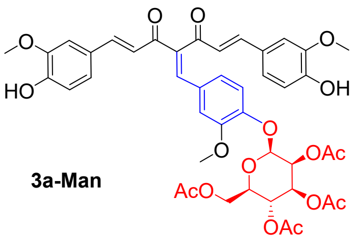

3a-Man

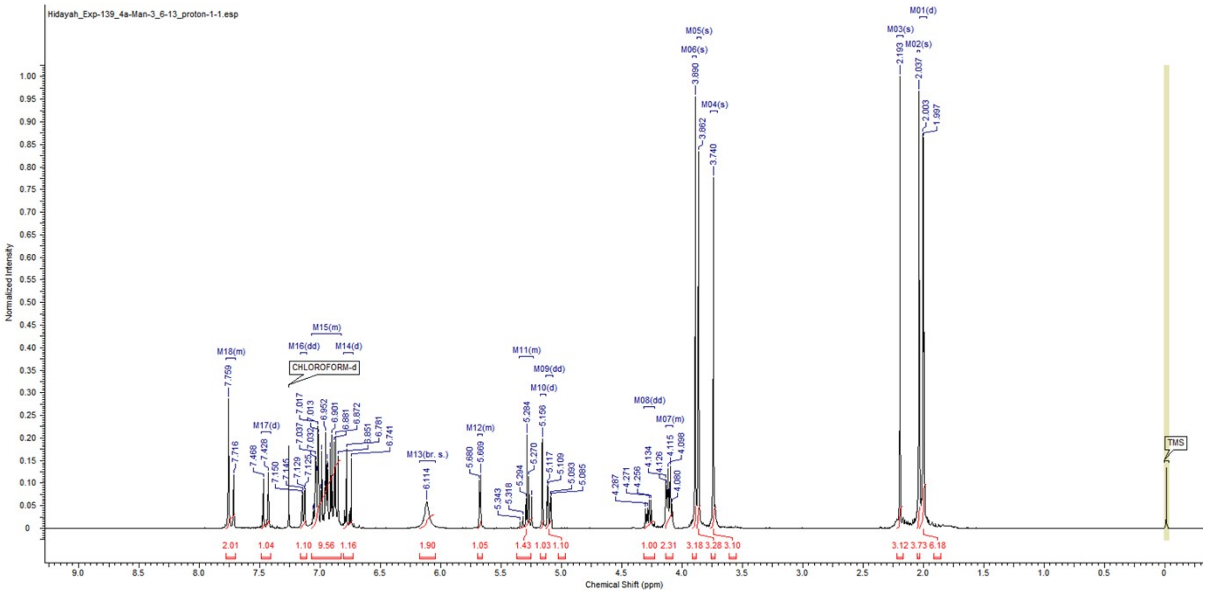

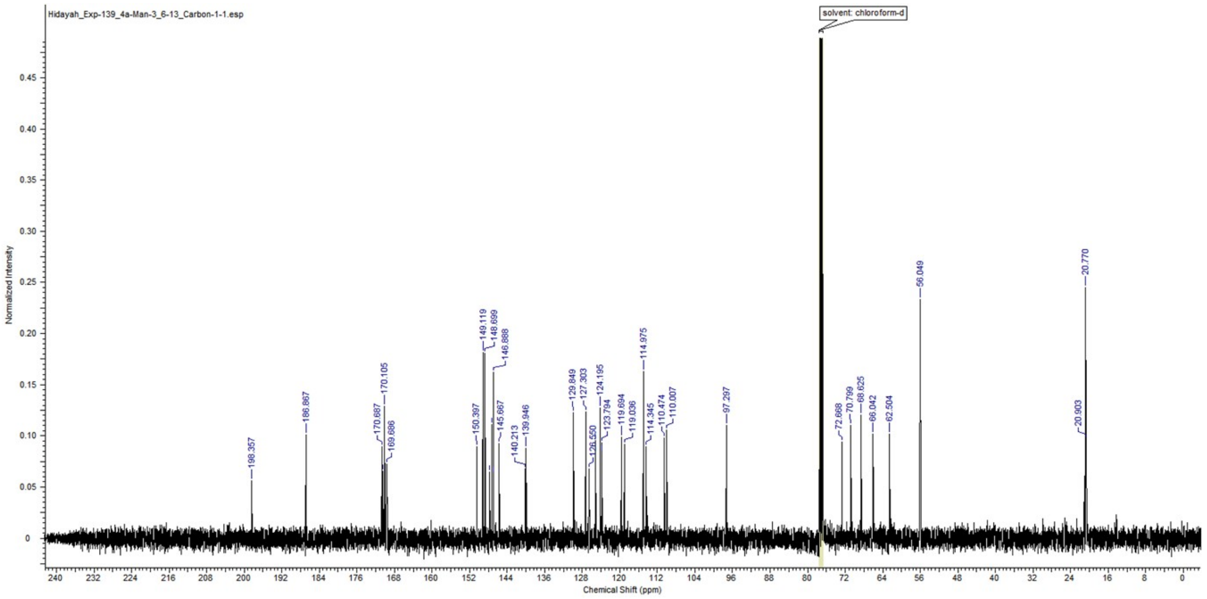

(2*R*,3*S*,4*S*,5*R*,6*S*)-2-(acetoxymethyl)-6-(3-((*E*)-5-(4-hydroxy-3-methoxyphenyl)-2-((*E*)-3-(4-hydroxy-3-methoxyphenyl)acryloyl)-3-oxopenta-1,4-dien-1-yl)phenoxy)tetrahydro-2*H*-pyran-3,4,5-triyl triacetate (**4a-Gal**)

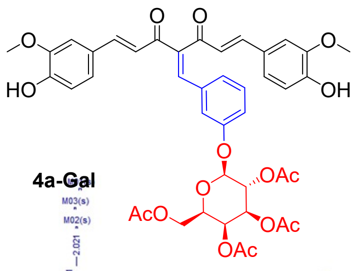

4a-Gal

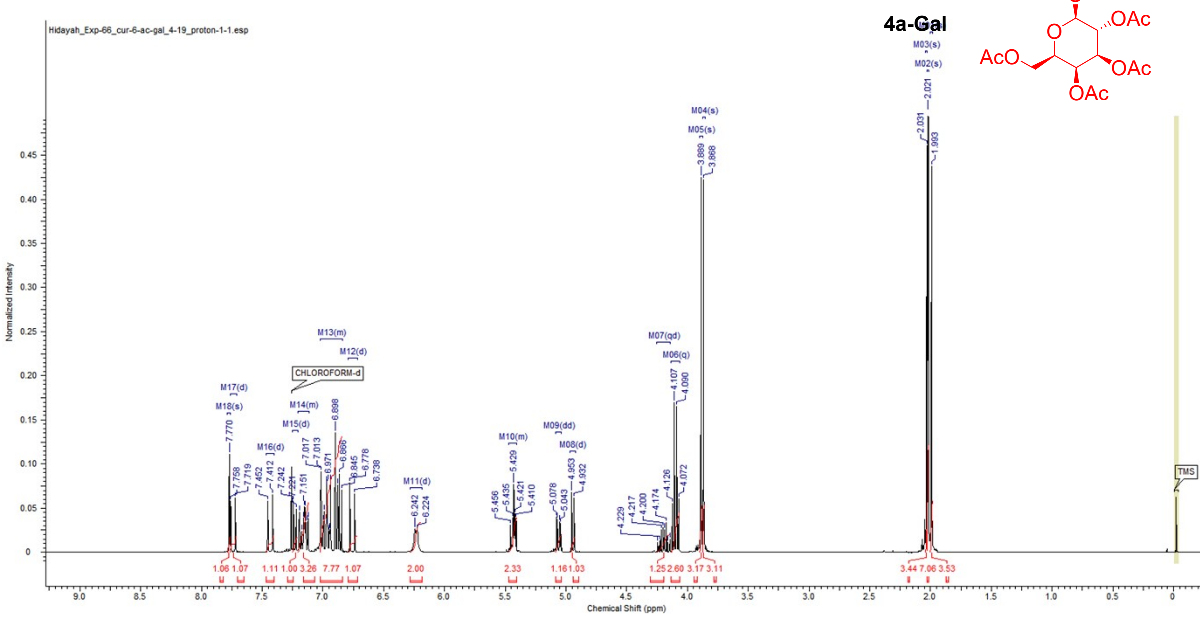

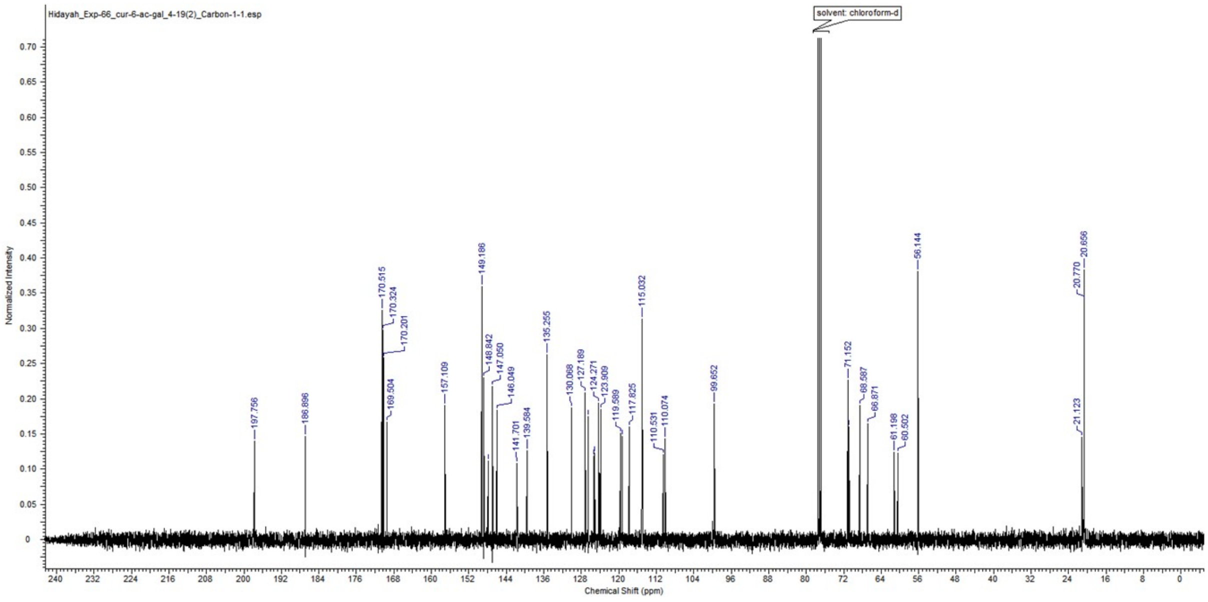

(2*R*,3*R*,4*S*,5*R*,6*S*)-2-(acetoxymethyl)-6-(3-((*E*)-5-(4-hydroxy-3-methoxyphenyl)-2-((*E*)-3-(4-hydroxy-3-methoxyphenyl)acryloyl)-3-oxopenta-1,4-dien-1-yl)phenoxy)tetrahydro-2*H*-pyran-3,4,5-triyl triacetate (**4a-Glc**)

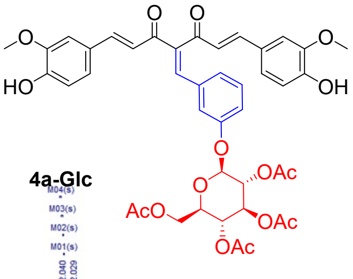

4a-Glc

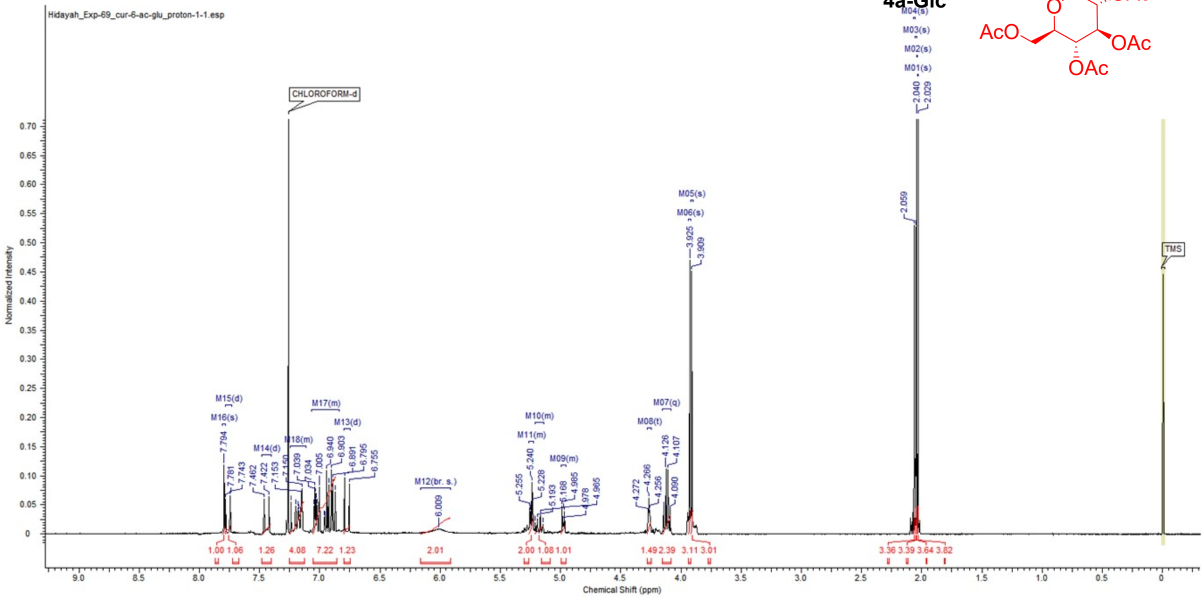

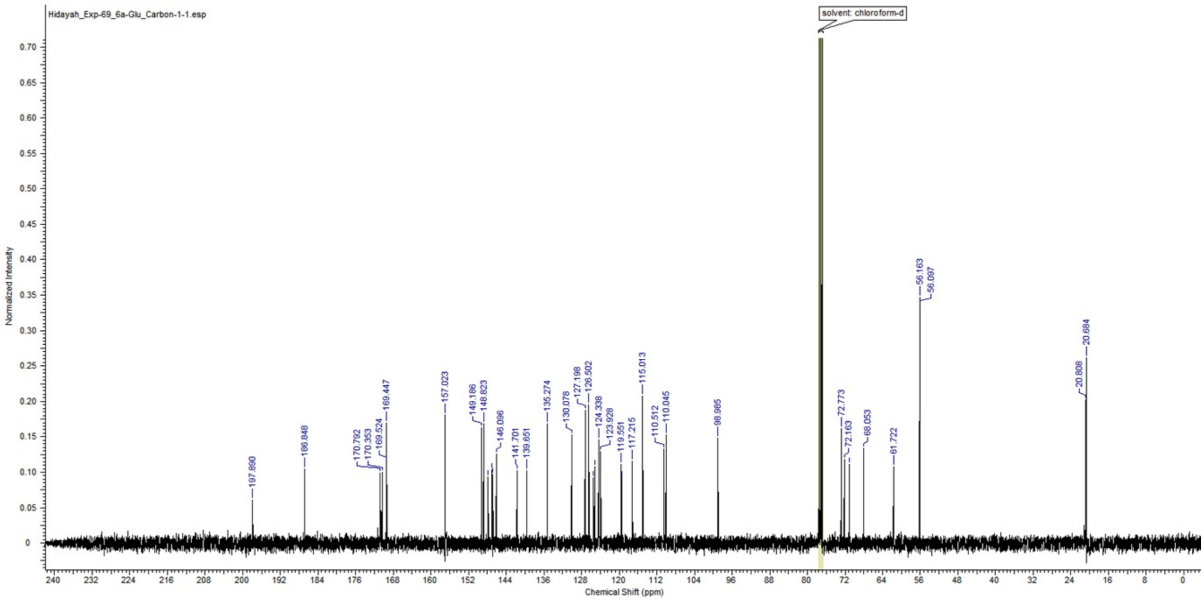

(3a*S*,5*R*,6*R*,7*S*,7a*S*)-5-(acetoxymethyl)-2-(3-((*E*)-5-(4-hydroxy-3-methoxyphenyl)-2-((*E*)-3-(4-hydroxy-3-methoxyphenyl)acryloyl)-3-oxopenta-1,4-dien-1-yl)phenoxy)-2-methyltetrahydro-5*H*-[1,3]dioxolo[4,5-b]pyran-6,7-diyl diacetate (**4a-Man**)

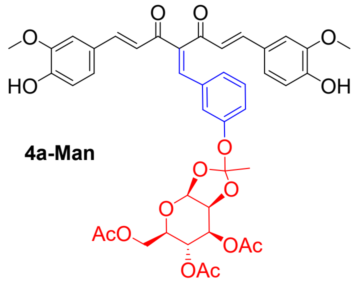

4a-Man

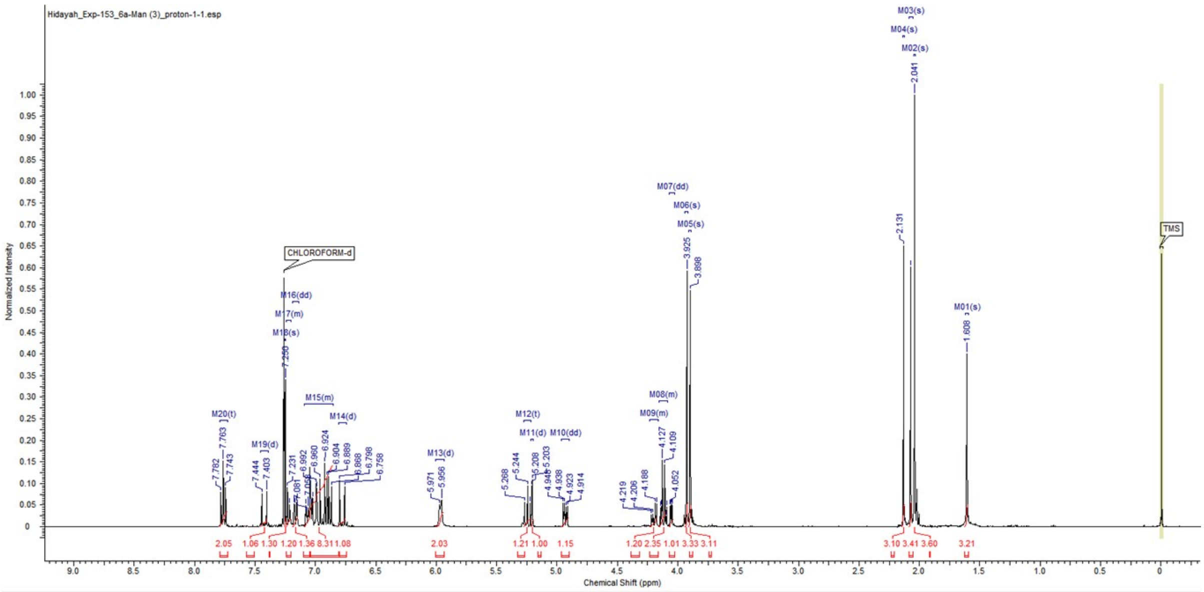

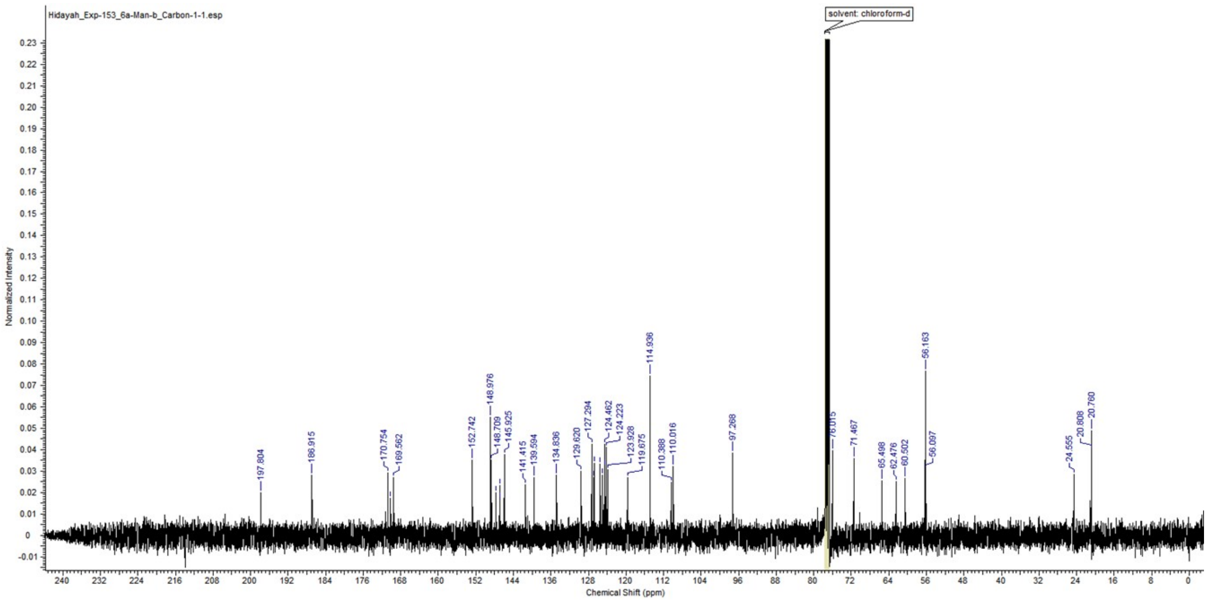

2.3 Deacetylated glycoside derivatives

1,7-bis(4-hydroxy-3-methoxyphenyl)-4-(4-(((2*S*,3*R*,4*S*,5*R*,6*R*)-3,4,5-trihydroxy-6-(hydroxymethyl)tetrahydro-2*H*-pyran-2-yl)oxy)benzylidene)hepta-1,6-diene-3,5-dione (**2b-Gal**)

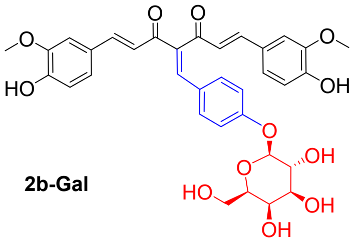

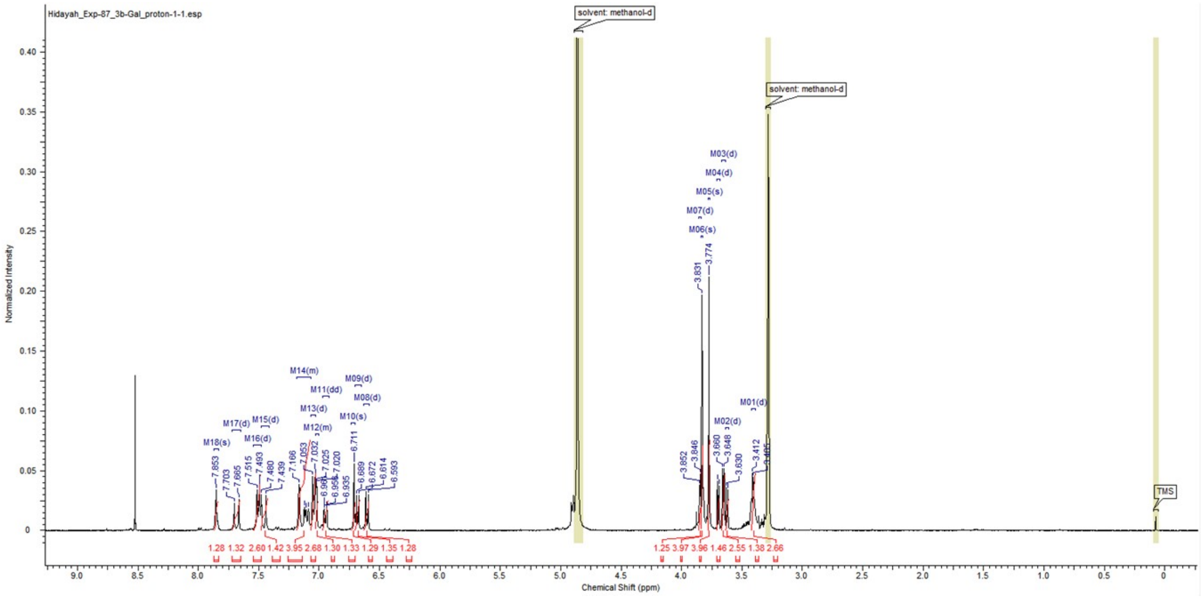

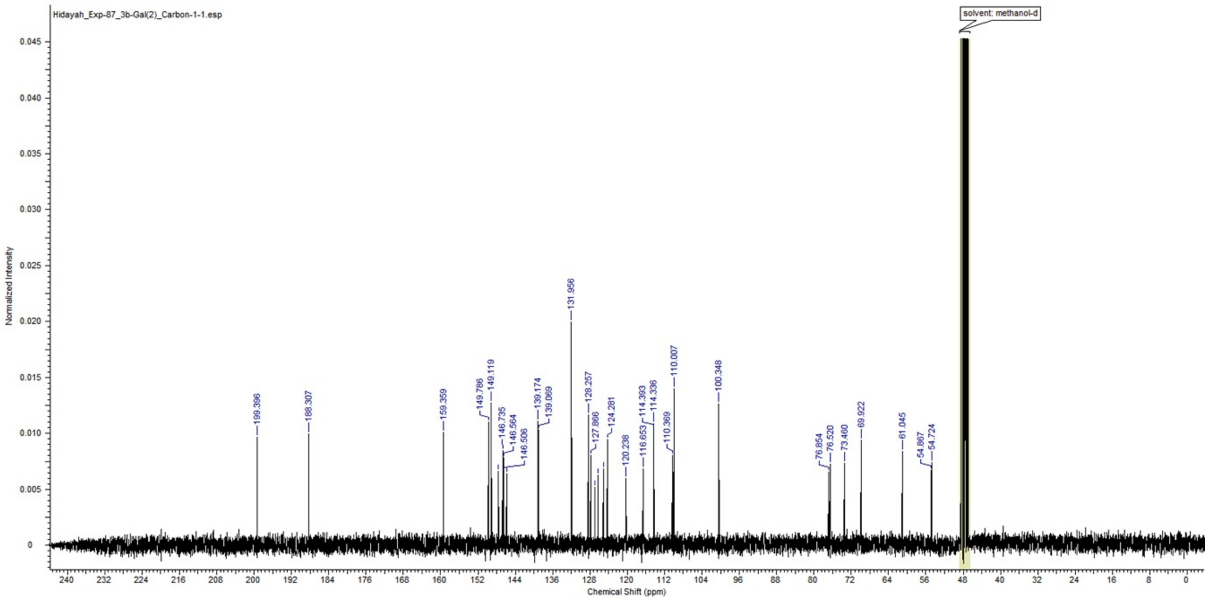

1,7-bis(4-hydroxy-3-methoxyphenyl)-4-(4-(((2*S*,3*R*,4*S*,5*S*,6*R*)-3,4,5-trihydroxy-6-(hydroxymethyl)tetrahydro-2*H*-pyran-2-yl)oxy)benzylidene)hepta-1,6-diene-3,5-dione (**2b-Glc**)

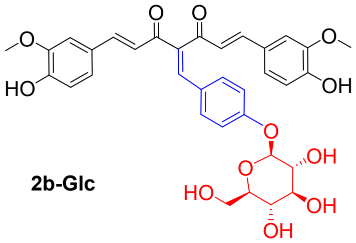

2b-Glc

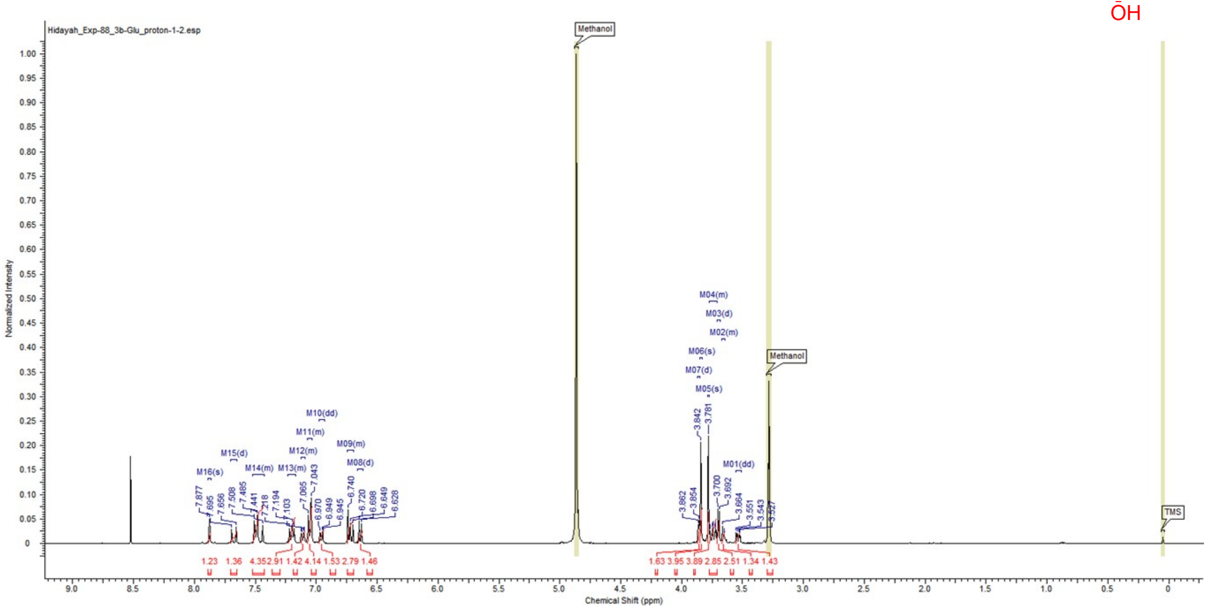

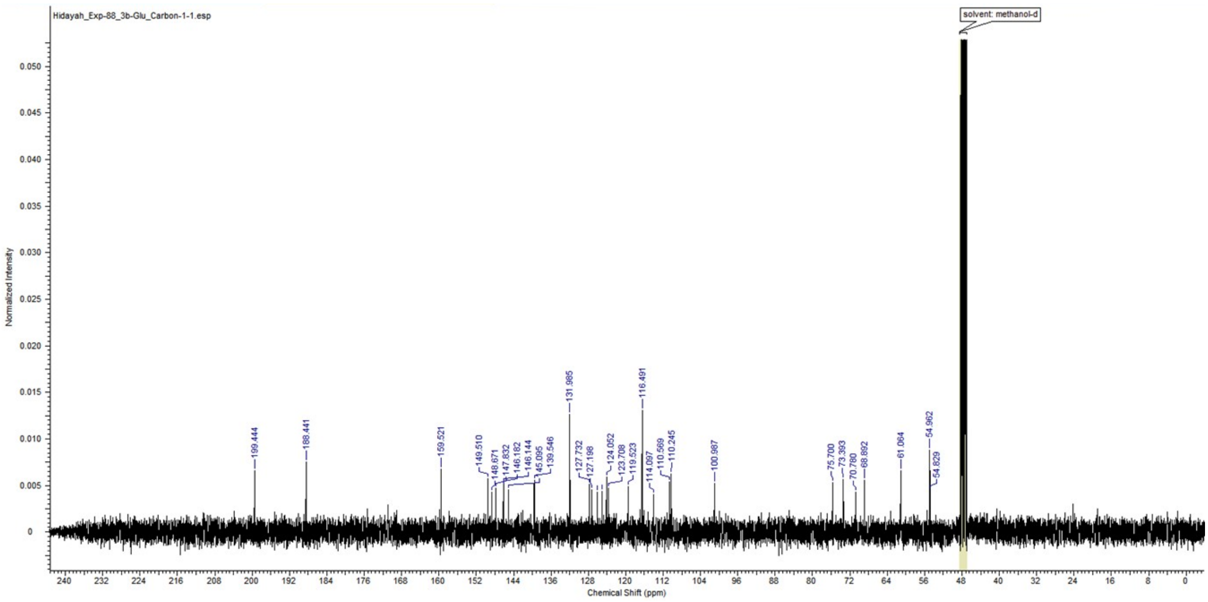

1,7-bis(4-hydroxy-3-methoxyphenyl)-4-(4-(((2*S*,3*S*,4*S*,5*S*,6*R*)-3,4,5-trihydroxy-6-(hydroxymethyl)tetrahydro-2*H*-pyran-2-yl)oxy)benzylidene)hepta-1,6-diene-3,5-dione (**2b-Man**)

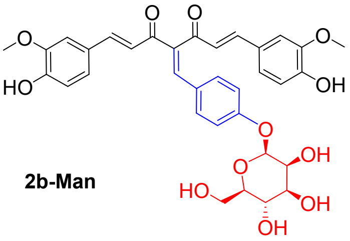

2b-Man

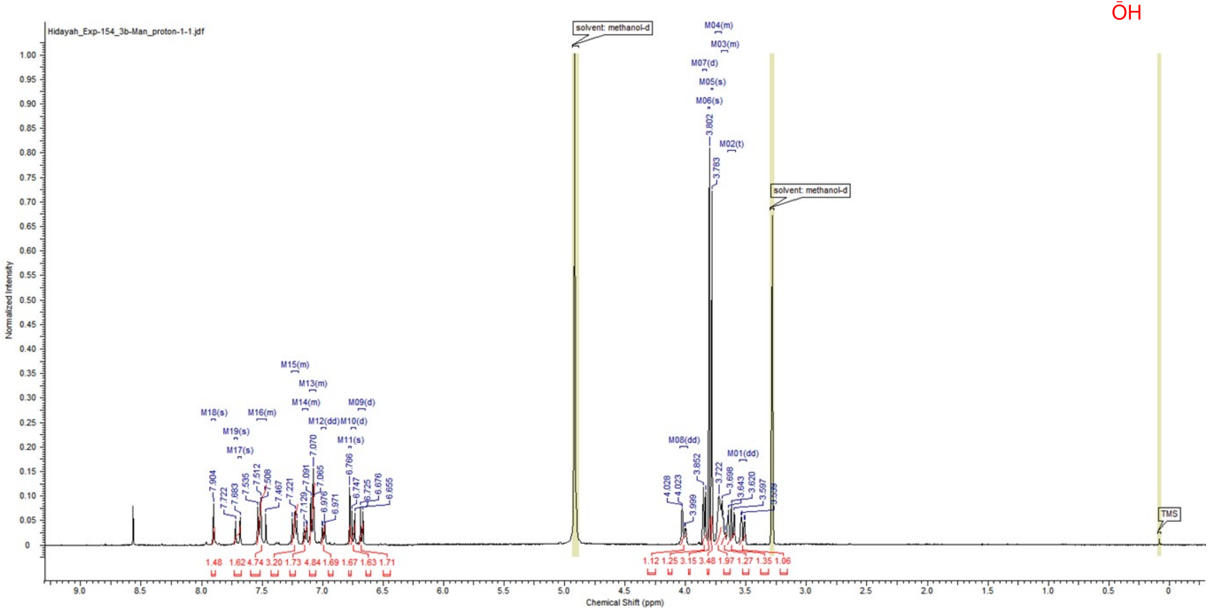

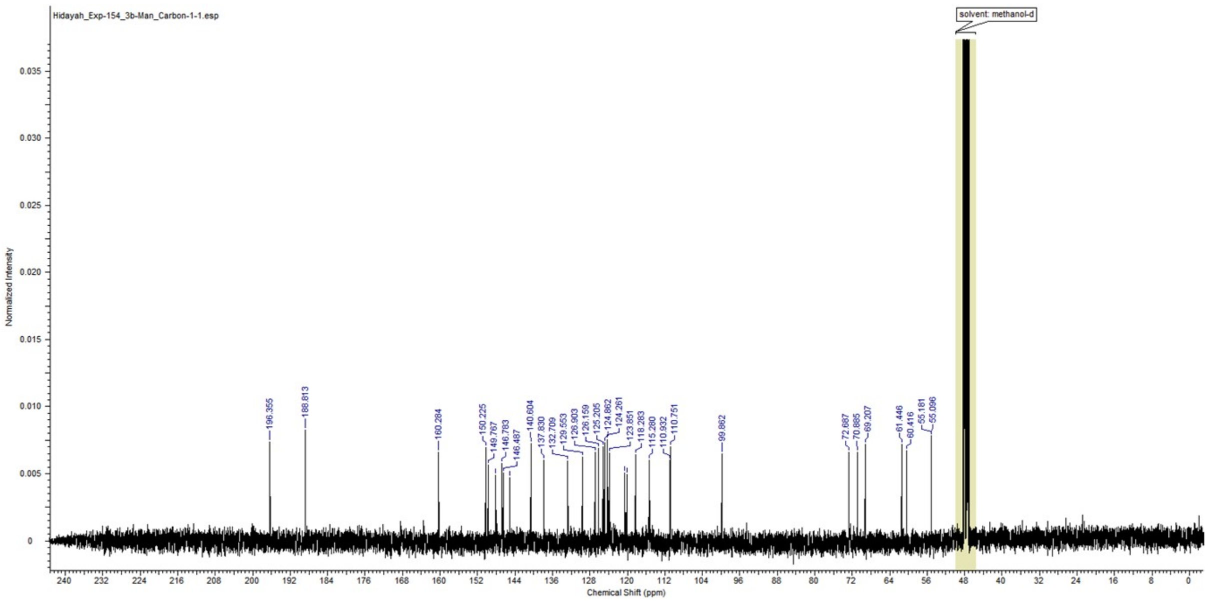

1,7-bis(4-hydroxy-3-methoxyphenyl)-4-(3-methoxy-4-(((2*S*,3*R*,4*S*,5*R*,6*R*)-3,4,5-trihydroxy-6-(hydroxymethyl)tetrahydro-2*H*-pyran-2-yl)oxy)benzylidene)hepta-1,6-diene-3,5-dione (**3b-Gal**)

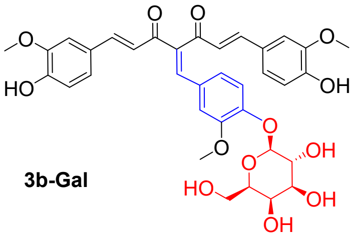

3b-Gal

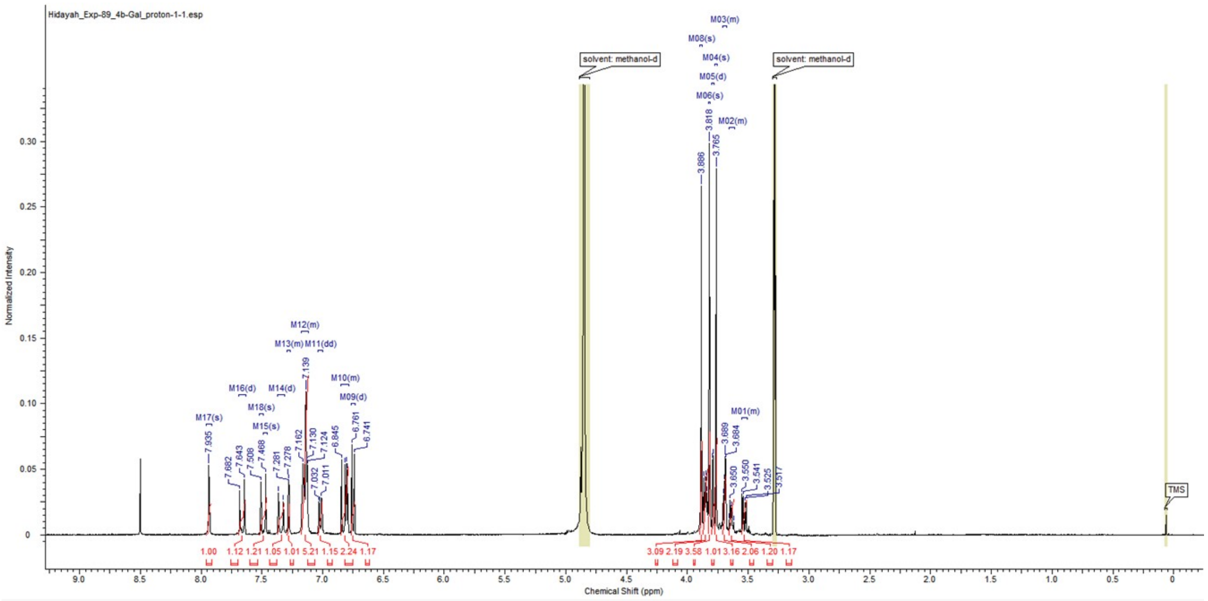

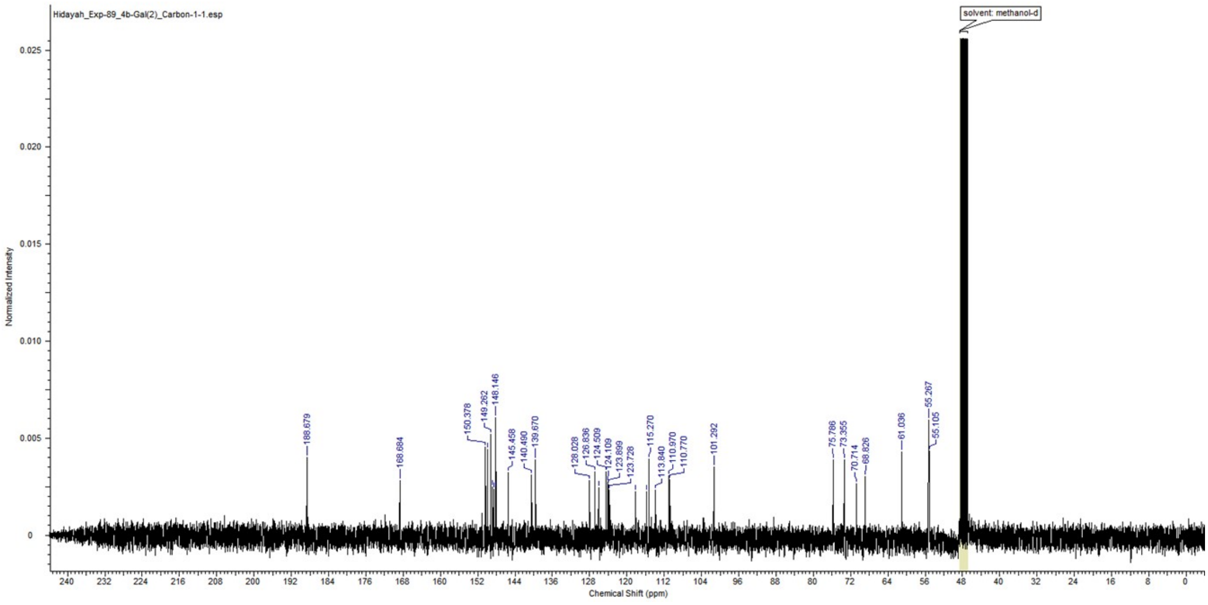

1,7-bis(4-hydroxy-3-methoxyphenyl)-4-(3-methoxy-4-(((2*S*,3*R*,4*S*,5*S*,6*R*)-3,4,5-trihydroxy-6-(hydroxymethyl)tetrahydro-2*H*-pyran-2-yl)oxy)benzylidene)hepta-1,6-diene-3,5-dione (**3b-Glc**)

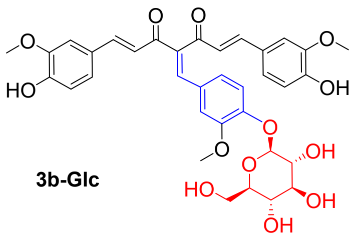

3b-Glc

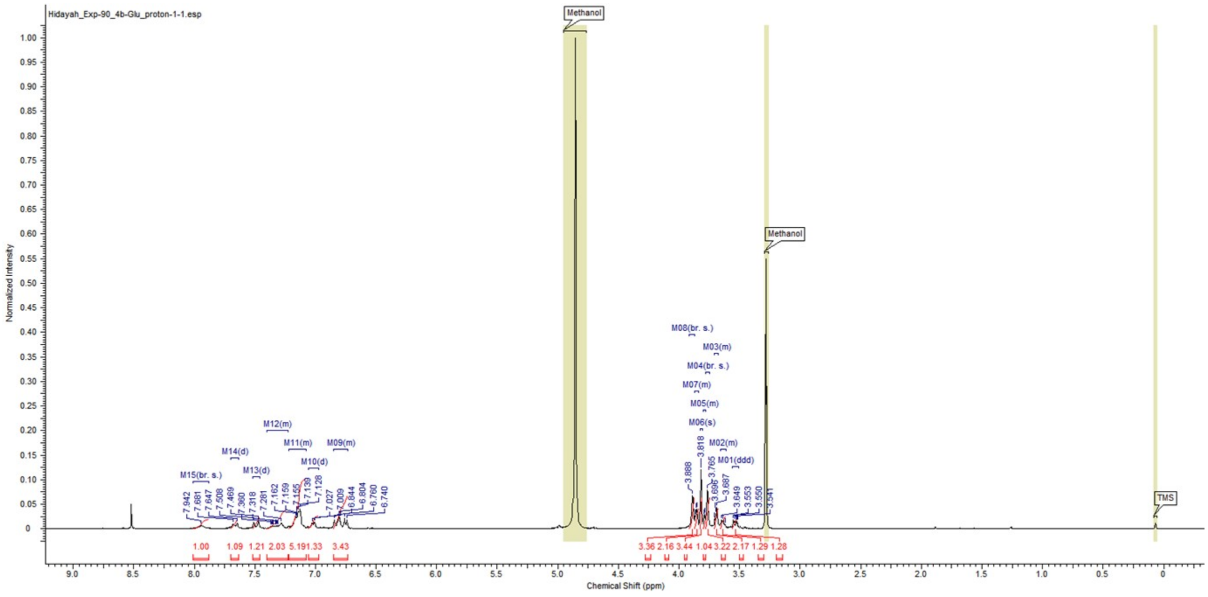

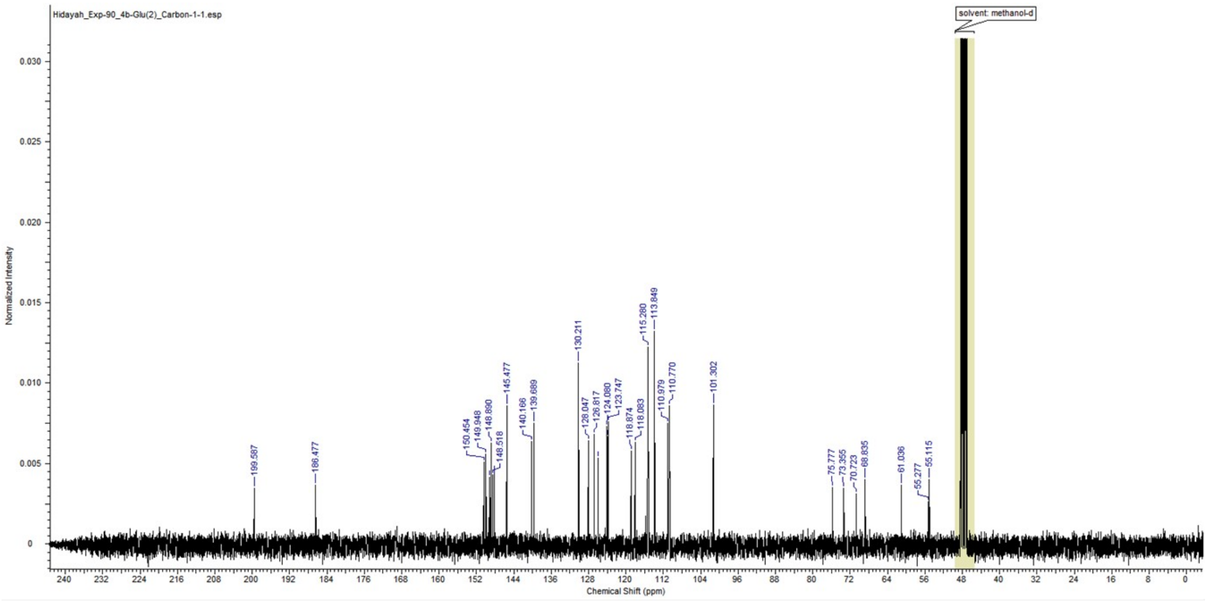

1,7-bis(4-hydroxy-3-methoxyphenyl)-4-(3-methoxy-4-(((2*S*,3*S*,4*S*,5*S*,6*R*)-3,4,5-trihydroxy-6-(hydroxymethyl)tetrahydro-2*H*-pyran-2-yl)oxy)benzylidene)hepta-1,6-diene-3,5-dione (**3b-Man**)

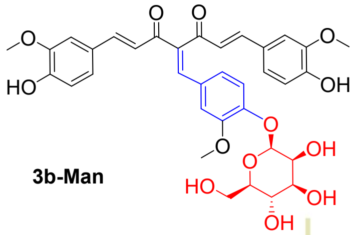

3b-Man

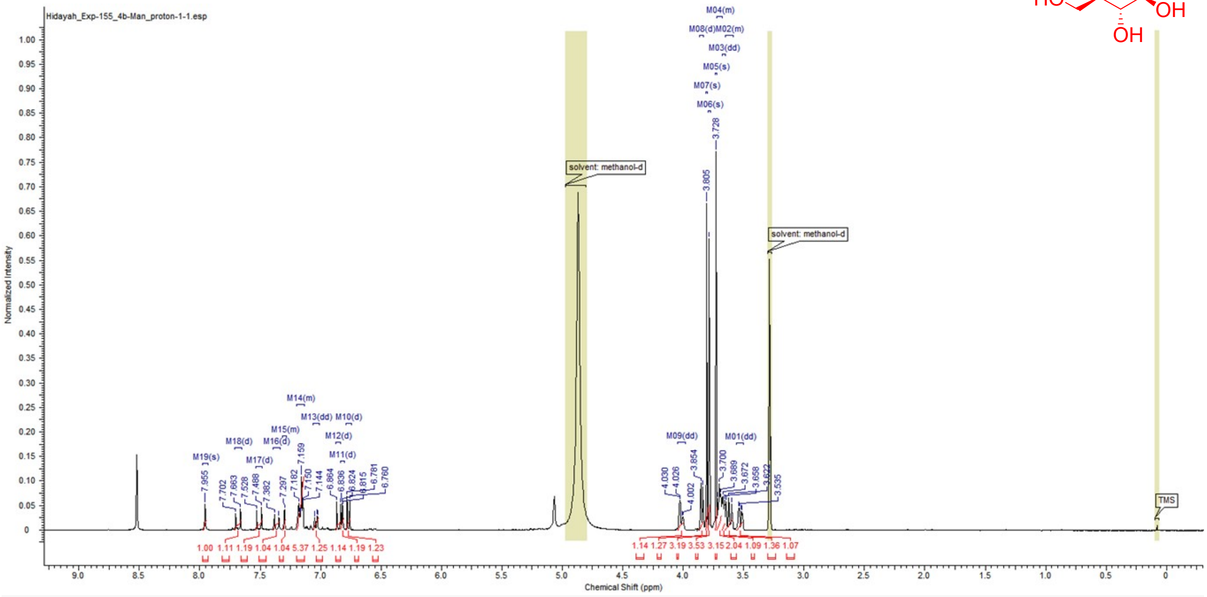

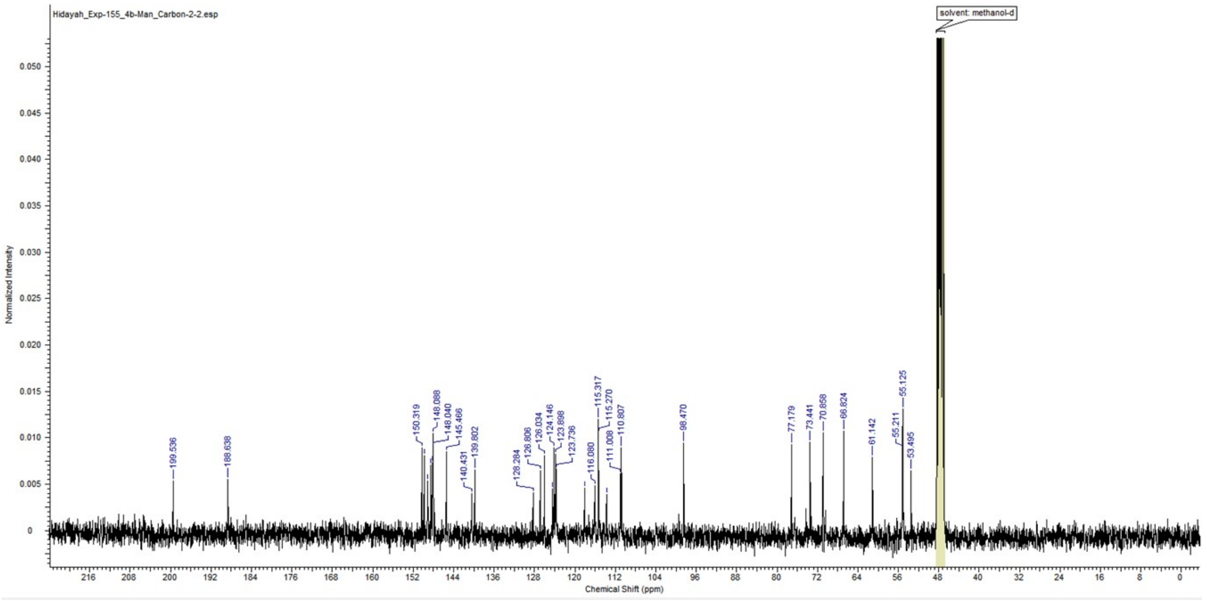

1,7-bis(4-hydroxy-3-methoxyphenyl)-4-(3-(((2*S*,3*R*,4*S*,5*R*,6*R*)-3,4,5-trihydroxy-6-(hydroxymethyl)tetrahydro-2*H*-pyran-2-yl)oxy)benzylidene)hepta-1,6-diene-3,5-dione (**4b-Gal**)

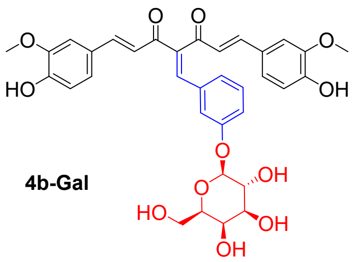

4b-Gal

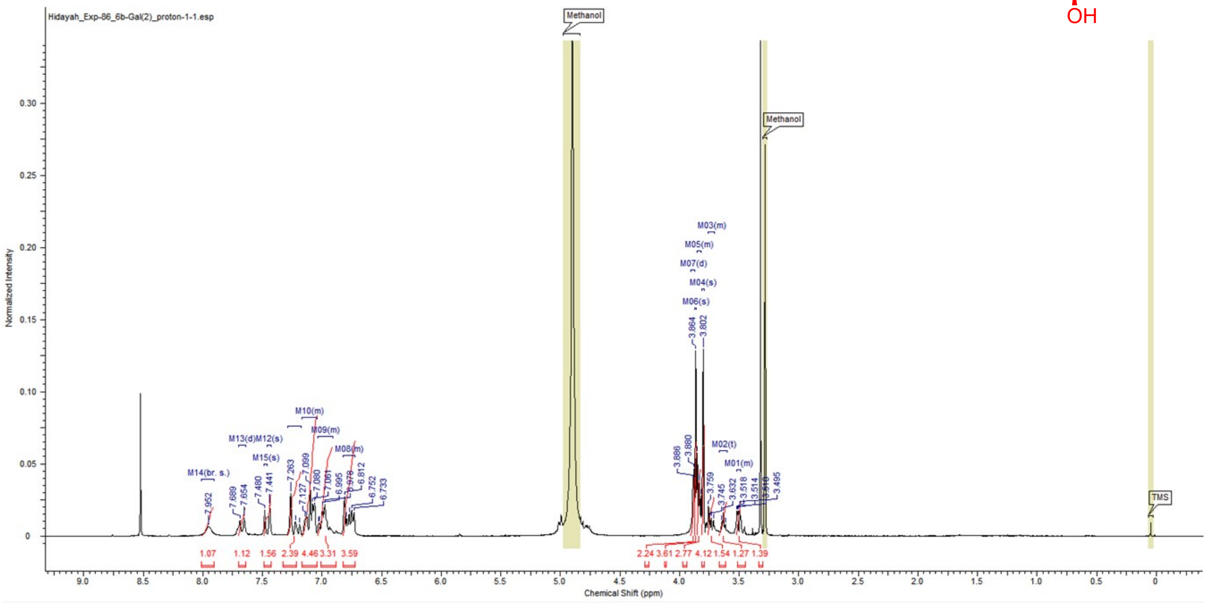

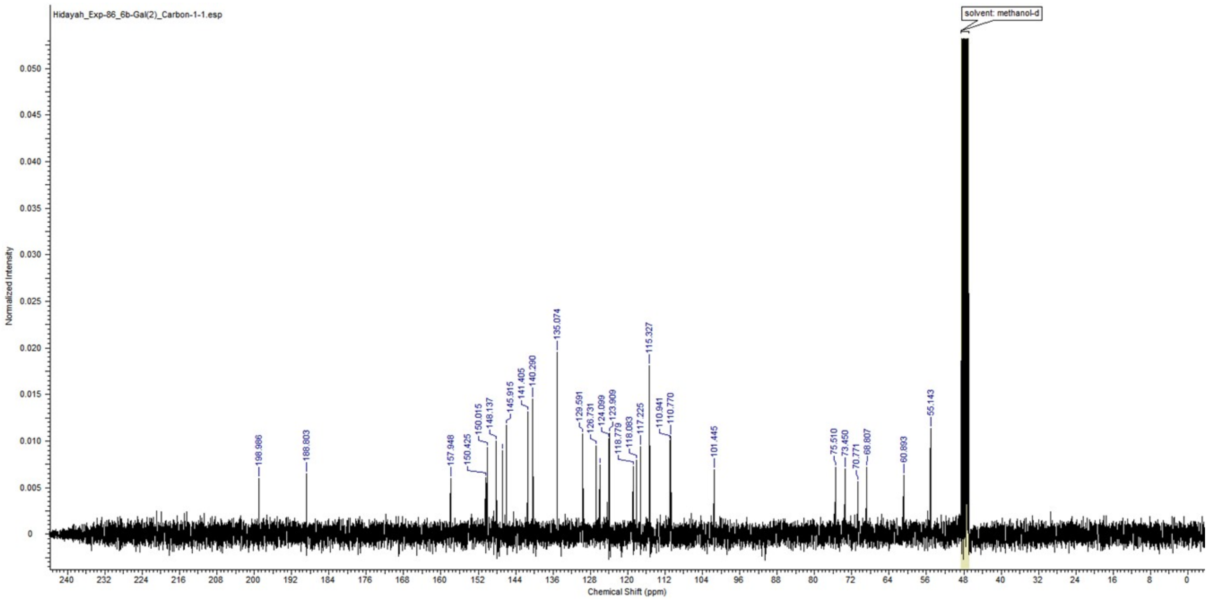

1,7-bis(4-hydroxy-3-methoxyphenyl)-4-(3-(((2*S*,3*R*,4*S*,5*S*,6*R*)-3,4,5-trihydroxy-6-(hydroxymethyl)tetrahydro-2*H*-pyran-2-yl)oxy)benzylidene)hepta-1,6-diene-3,5-dione (**4b-Glc**)

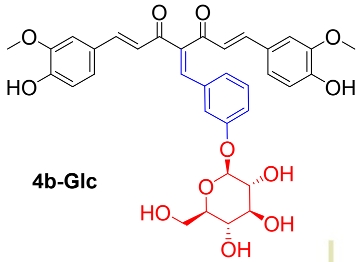

4b-Glc

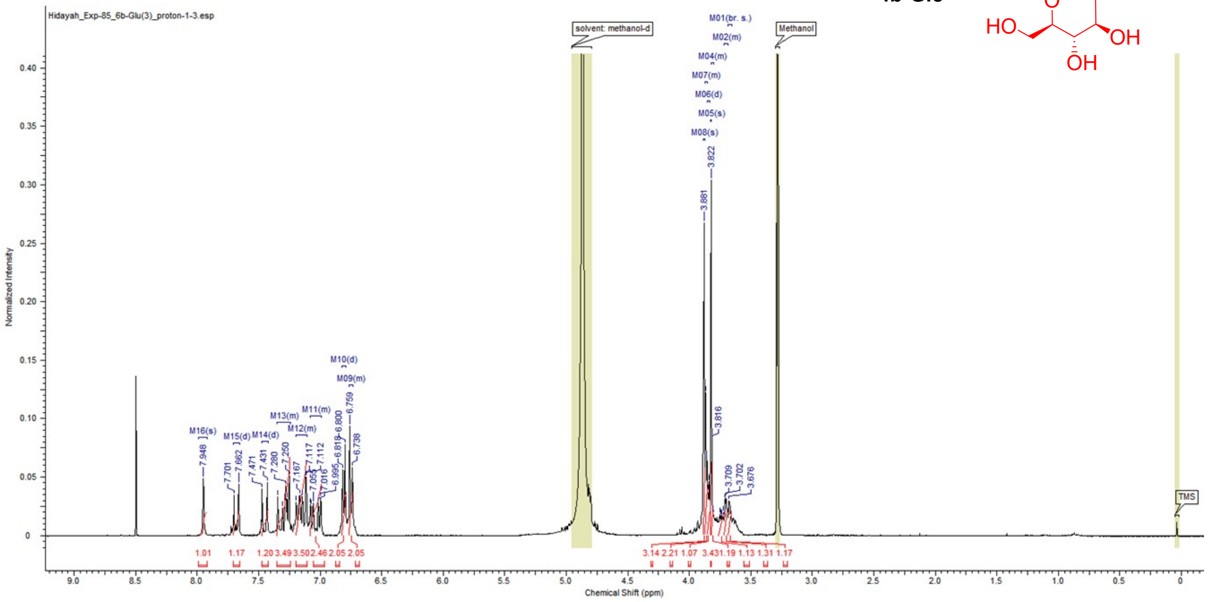

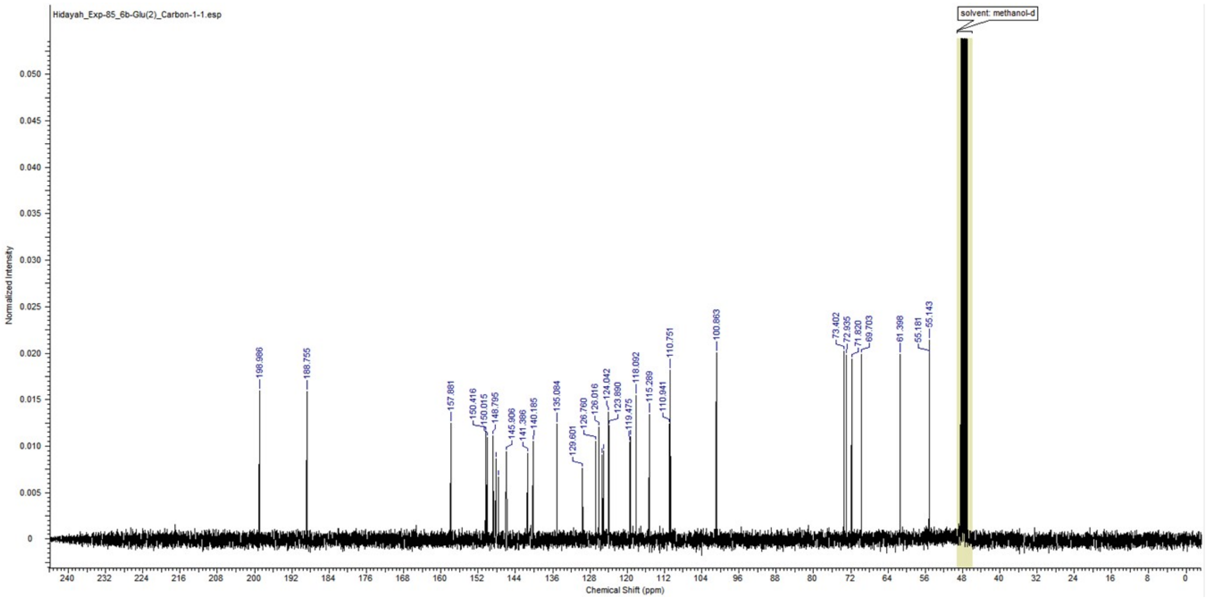

4-(3-(((3a*S*,5*R*,6*S*,7*S*,7a*S*)-6,7-dihydroxy-5-(hydroxymethyl)-2-methyltetrahydro-5*H*-[1,3]dioxolo[4,5-b]pyran-2-yl)oxy)benzylidene)-1,7-bis(4-hydroxy-3-methoxyphenyl)hepta-1,6-diene-3,5-dione (**4b-Man**)

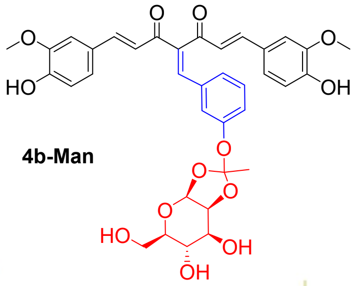

4b-Man

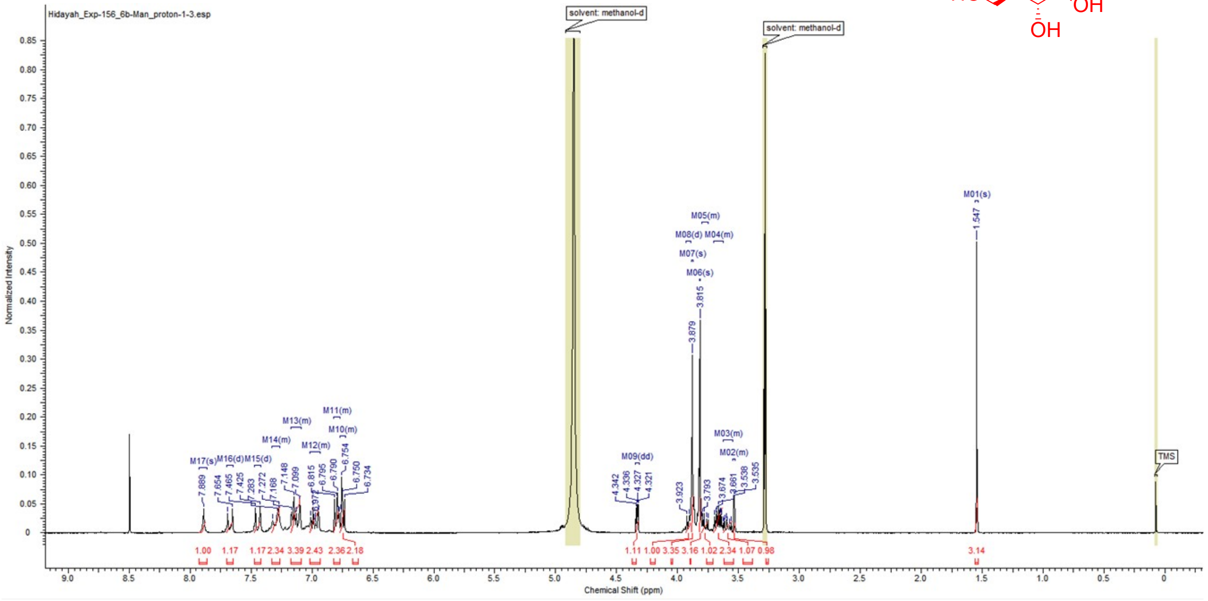

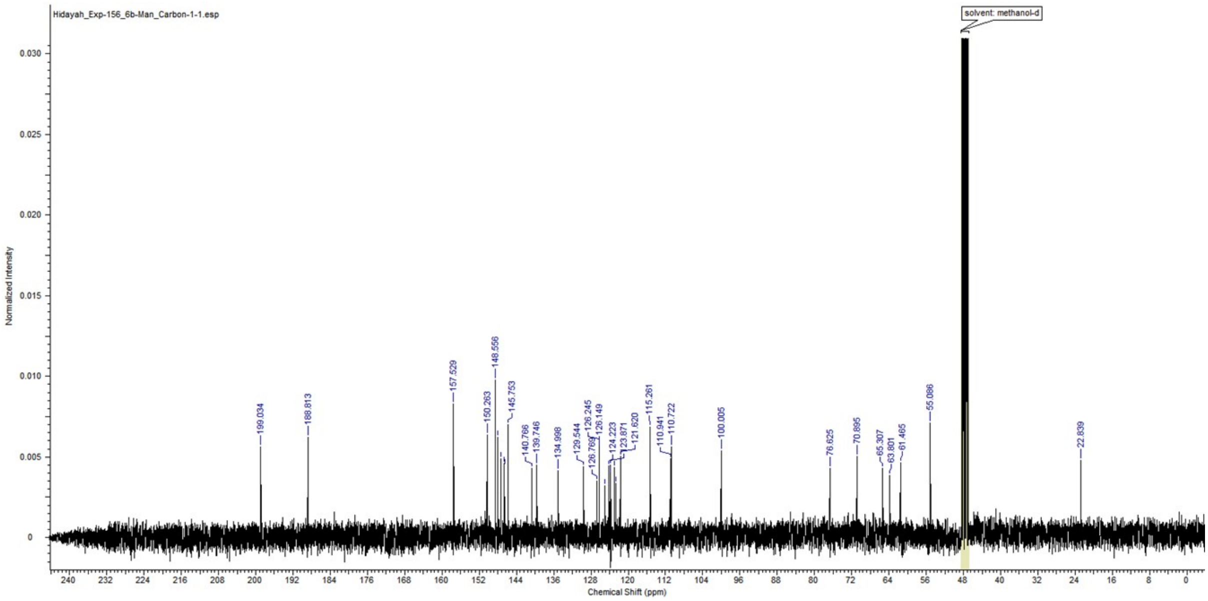

3. *In vitro* pLDH antiplasmodial activity

Graphs of parasite growth against the concentration of glycoside compounds from pLDH assays

2a-Gal against 3D7

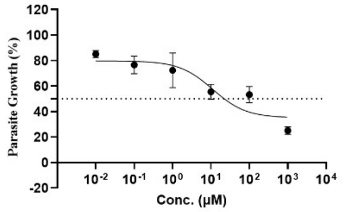

3a-Gal against 3D7

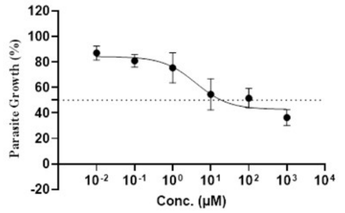

4a-Gal against 3D7

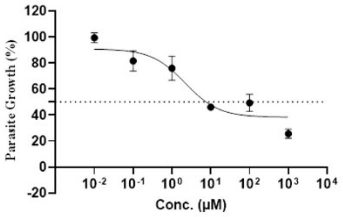

2a-Gal against K1

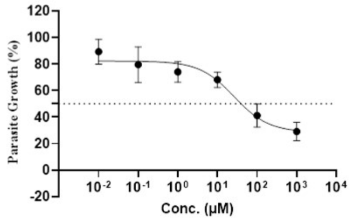

3a-Gal against K1

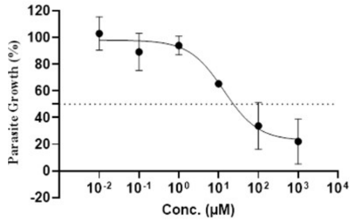

4a-Gal against K1

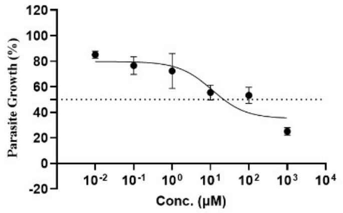

2b-Gal against 3D7

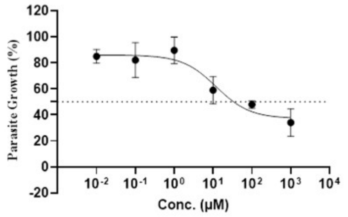

3b-Gal against 3D7

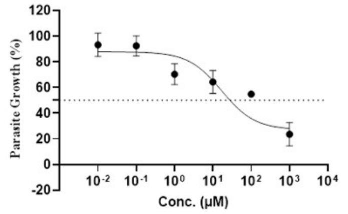

4b-Gal against 3D7

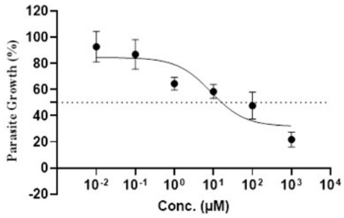

2b-Gal against K1

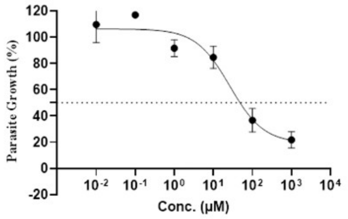

3b-Gal against K1

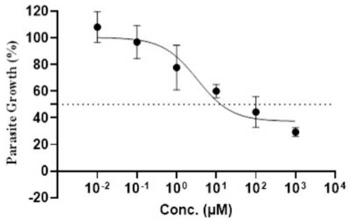

4b-Gal against K1

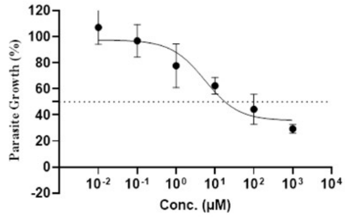

2a-Glc against 3D7

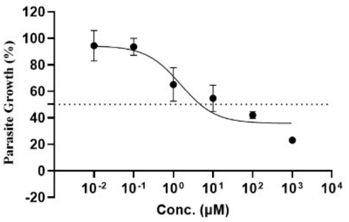

3a-Glc against 3D7

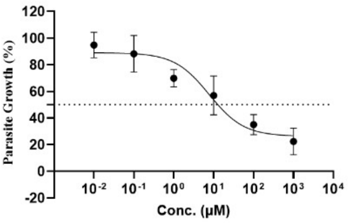

4a-Glc against 3D7

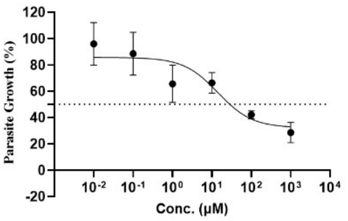

2a-Glc against K1

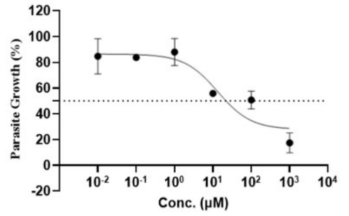

3a-Glc against K1

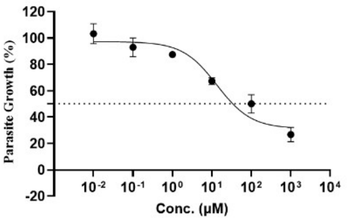

4a-Glc against K1

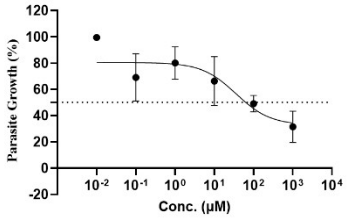

2b-Glc against 3D7

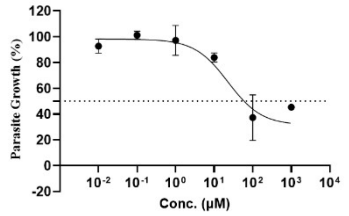

3b-Glc against 3D7

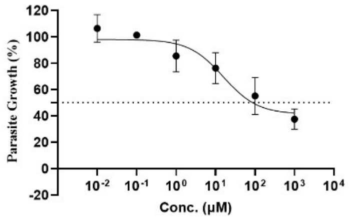

4b-Glc against 3D7

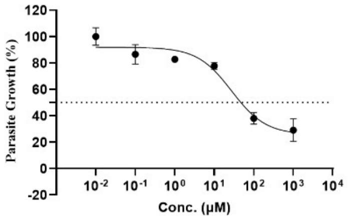

2b-Glc against K1

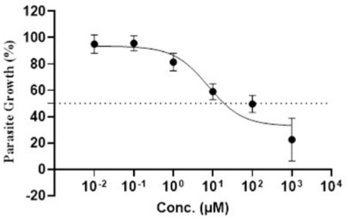

3b-Glc against K1

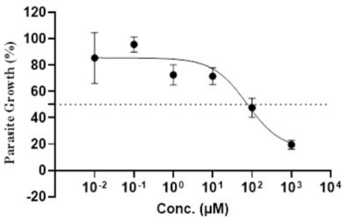

4b-Glc against K1

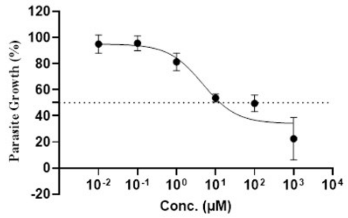

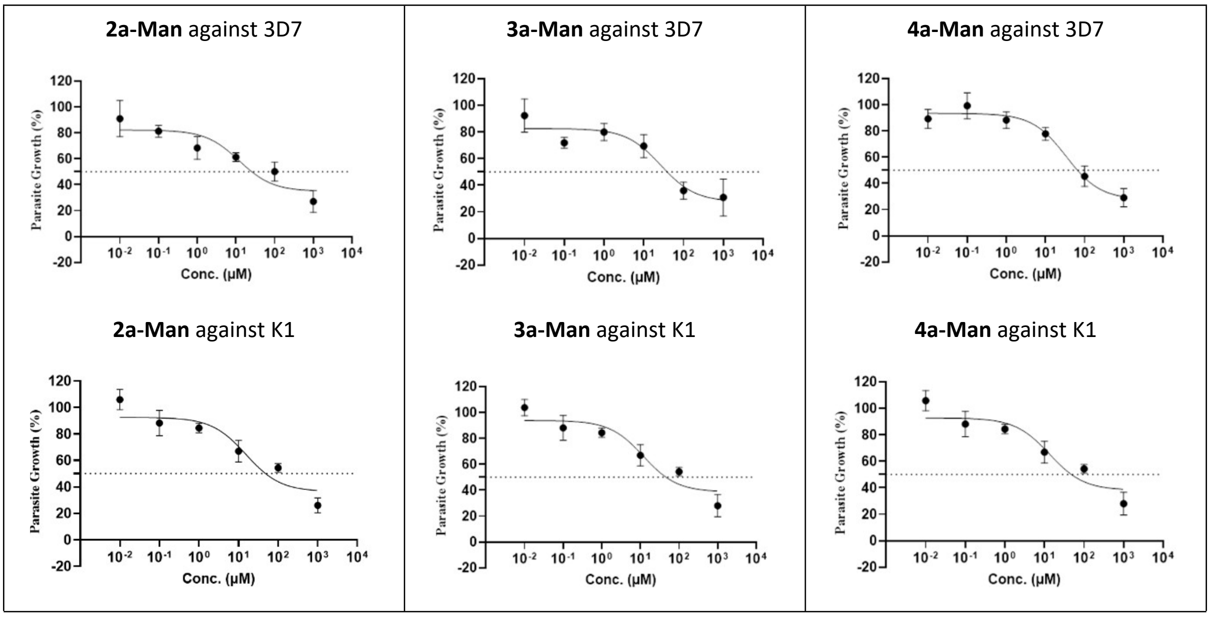
2a-Man against 3D7
3a-Man against 3D7
4a-Man against 3D7
2a-Man against K1
3a-Man against K1
4a-Man against K1
Parasite Growth (%)
Conc. (μM)

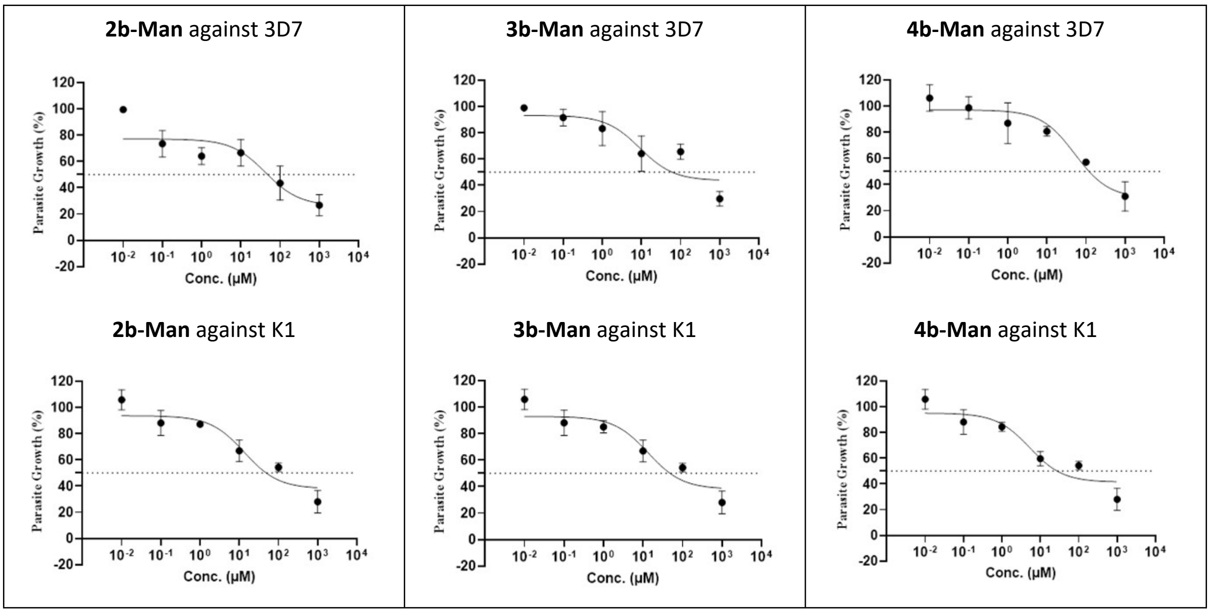
2b-Man against 3D7
3b-Man against 3D7
4b-Man against 3D7
2b-Man against K1
3b-Man against K1
4b-Man against K1
Parasite Growth (%)
Conc. (μM)

4. Cytotoxicity from MTT WRL-68 assay

Graphs of cell viability against the log of concentration of glycoside derivative compounds from the MTT WRL-68 assay

2a-Gal

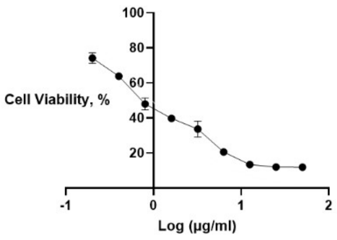

3a-Gal

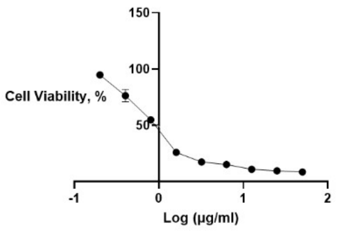

4a-Gal

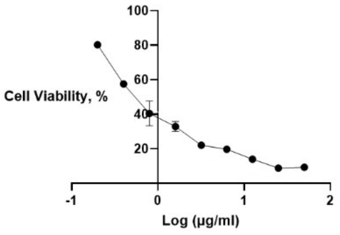

2b-Gal

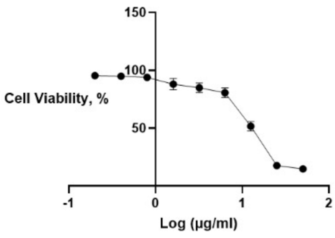

3b-Gal

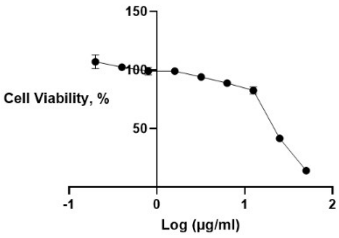

4b-Gal

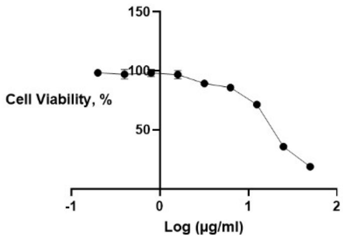

2a-Glc

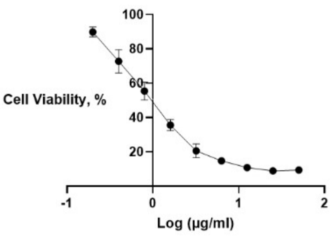

3a-Glc

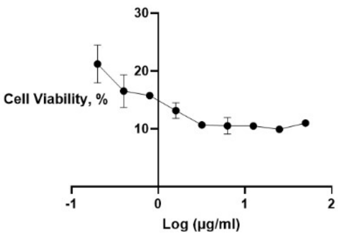

4a-Glc

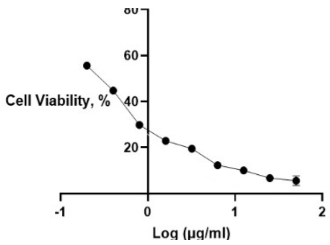

2b-Glc

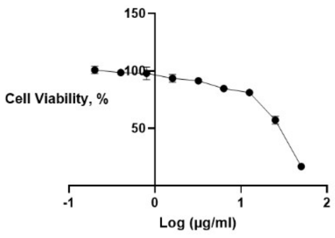

3b-Glc

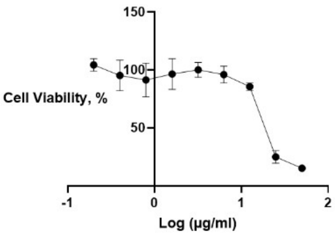

4b-Glc

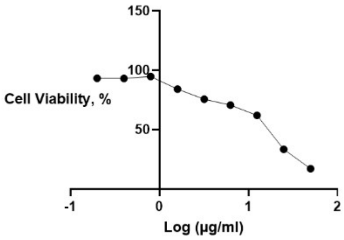

2a-Man

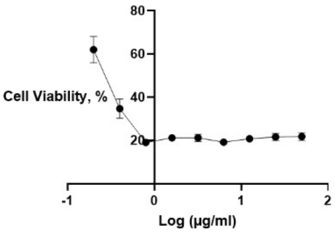

3a-Man

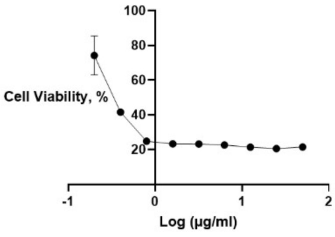

4a-Man

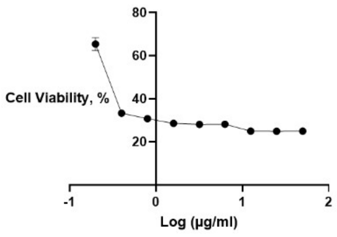

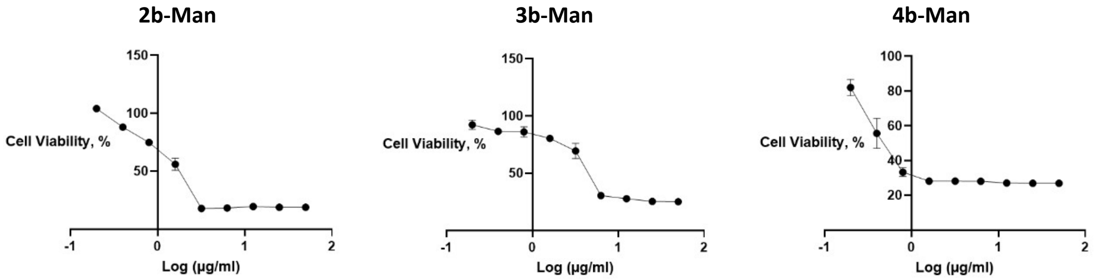
2b-Man
Cell Viability, %
Log (µg/ml)
3b-Man
Cell Viability, %
Log (µg/ml)
4b-Man
Cell Viability, %
Log (µg/ml)

5. Binding interactions to GSK-3β from molecular docking

Curcumin glycoside derivatives categorised according to the type of conjugated sugar and the affinity towards GSK-3β

Compound	Binding energy, kcal/mol	Inhibition constant (K_i), μM	Type of interaction	Amino acid residues involved in binding
**1**	−7.44	1.02	Hydrogen bonds	Asp133, Tyr134, Arg141, Asn186, Cys199
			Hydrophobic interactions	Ile62, Val70, Ala83, Leu188, Cys199
**3**	−9.42	1.04	Hydrogen bonds	Ile62, Asp133, Tyr134, Pro136, Arg144, Cys199
			Hydrophobic interactions	Ile62, Val70, Ala83, Val110, Leu132, Arg141, Leu188, Cys199
**4**	−9.07	1.01	Hydrogen bonds	Val61, Arg141, Asp133
			Hydrophobic interactions	Ile62, Gly63, Val70, Ala83, Leu132, Leu188, Cys199
**6**	−9.08	0.28	Hydrogen bonds	Ile62, Lys85, Val135, Arg141, Cys199, Asp200
			Hydrophobic interactions	Ile62, Val70, Ala83, Lys85, Val110, Leu132, Tyr134, Cys199
Galactoside derivatives				
**3a-Gal**	−8.23	0.92	Hydrogen bonds	Lys60, Tyr134, Val135, Arg141
			Hydrophobic interactions	Ile62, Val70, Cys122
**4a-Gal**	−8.33	0.78	Hydrogen bonds	Ile62, Gln172, Tyr134, Val135, Pro136, Arg141, Cys199, Asp200
			Hydrophobic interactions	Ile62, Val70, Ala83, Val110, Leu132, Tyr140, Cys199
**6a-Gal**	−8.12	1.13	Hydrogen bonds	Val61, Gly63, Gly65, Asp133, Tyr134, Arg141, Asn186
			Hydrophobic interactions	Lys60, Ile62, Ala83, Tyr134, Arg141, Leu188
**3b-Gal**	−9.54	0.10	Hydrogen bonds	Val61, Ile62, Tyr134, Val135, Arg141, Arg144, Asp200
			Hydrophobic interactions	Lys60, Ile62, Val70, Ala83, Lys85, Val110, Leu132, Thr138, Leu188, Cys199
**4b-Gal**	−10.21	0.03	Hydrogen bonds	Ile62, Lys85, Glu97, Val135, Arg141, Arg144, Asn186, Asp200
			Hydrophobic interactions	Ile62, Val70, Lys85, Val110, Leu132, Cys199
**6b-Gal**	−10.31	0.03	Hydrogen bonds	Val61, Ile62, Asn64, Lys85, Glu97, Tyr134, Val135, Aps200
			Hydrophobic interactions	Lys60, Ile62, Val70, Lys85, Val110, Leu132, Cys199
Glucoside derivatives				
**3a-Glc**	−10.48	0.02	Hydrogen bonds	Lys85, Glu97, Val135, Arg141, Arg144, Aps200

			Hydrophobic interactions	Ile62, Val70, Lys85, Val110, Leu132, Cys199
**4a-Glc**	–10.14	0.04	Hydrogen bonds	Asn64, Tyr134, Val135, Arg141, Arg144
			Hydrophobic interactions	Lys60, Ile62, Ala83, Val110, Leu132, Leu188, Cys199
**6a-Glc**	–9.69	0.08	Hydrogen bonds	Lys60, Gln72, Tyr134, Val135, Cys199
			Hydrophobic interactions	Ile62, Gly63, Val70, Lys85
**3b-Glc**	–9.75	0.07	Hydrogen bonds	Ile62, Lys85, Glu97, Tyr134, Val135, Pro136, Aps200
			Hydrophobic interactions	Ile62, Val70, Lys85, Met101, Val110, Leu132, Cys199
**4b-Glc**	–9.59	0.09	Hydrogen bonds	Ile62, Lys85, Glu97, Tyr134, Val135, Pro136, Arg141, Asp200
			Hydrophobic interactions	Ile62, Val70, Lys85, Met101, Val110, Leu132, Thr138, Arg141, Cys199
**6b-Glc**	–10.53	0.02	Hydrogen bonds	Gln72, Lys85, Glu97, Tyr134, Val135, Pro136, Aps200, Phe201
			Hydrophobic interactions	Ile62, Val70, Lys85, Met101, Val110, Leu132, Cys199
Mannoside derivatives				
**3a-Man**	–8.12	1.12	Hydrogen bonds	Gly63, Asn64, Gly65, Lys85, Tyr134, Val135, Cys199, Asp200
			Hydrophobic interactions	Ile62, Val70, Lys85, Met101, Val110, Leu132, Cys199
**4a-Man**	–9.38	0.13	Hydrogen bonds	Ile62, Glu97, Tyr134, Val135, Arg141, Asn186, Cys199, Asp200
			Hydrophobic interactions	Ile62, Val70, Lys85, Met101, Val110, Leu132, Cys199
**6a-Man**	–10.14	0.04	Hydrogen bonds	Val61, Gly63, Val135, Asn186, Cys199, Asp200
			Hydrophobic interactions	Ile62, Val70, Lys85, Met101, Val110, Leu132, Tyr134,Cys199
**3b-Man**	–10.35	0.03	Hydrogen bonds	Ile62, Gly63, Lys85, Glu97, Val135, Asp200, Phe201
			Hydrophobic interactions	Ile62, Val70, Lys85, Met101, Val110, Leu132, Cys199
**4b-Man**	–8.02	1.33	Hydrogen bonds	Lys60, Val61, Ile62, Gln72, Asp133, Tyr134, Arg141
			Hydrophobic interactions	Val61, Ile62, Val70, Ala83, Leu188, Cys199
**6b-Man**	–9.53	0.10	Hydrogen bonds	Lys60, Gln72, Tyr143, Val135, Asn186, Cys199
			Hydrophobic interactions	Lys60, Ile62, Val70, Val110, Leu132, Leu188, Cys199

Visualisation of the docking of the glycoside derivatives to GSK-3β, showing the intermolecular interactions between the ligand and the amino acid residues of the protein

**2a-Gal**: –8.23 kcal/mol 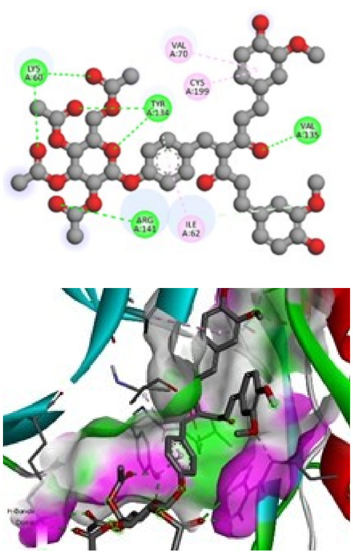	**3a-Gal**: –8.33 kcal/mol 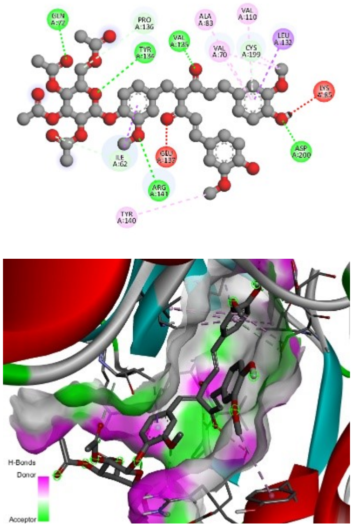	**4a-Gal**: –8.12 kcal/mol 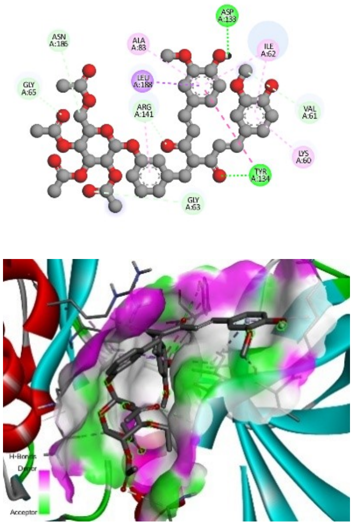

**2b-Gal**: –9.54 kcal/mol 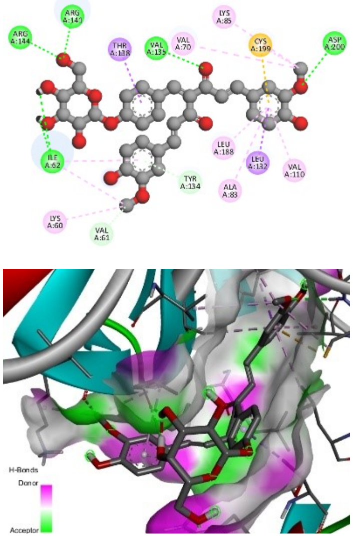	**3b-Gal**: –10.21 kcal/mol 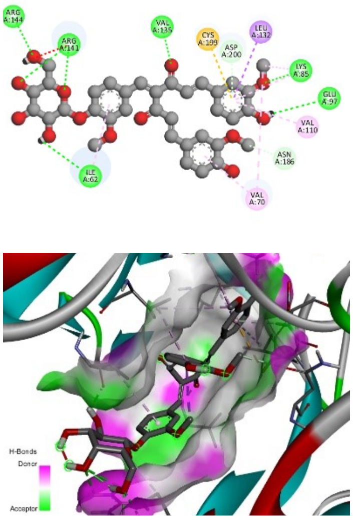	**4b-Gal**: –10.31 kcal/mol 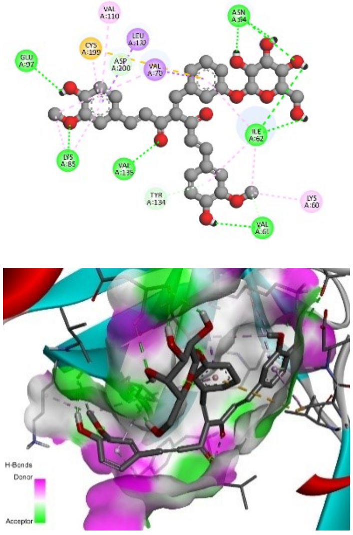

2a-Glc: –10.48 kcal/mol

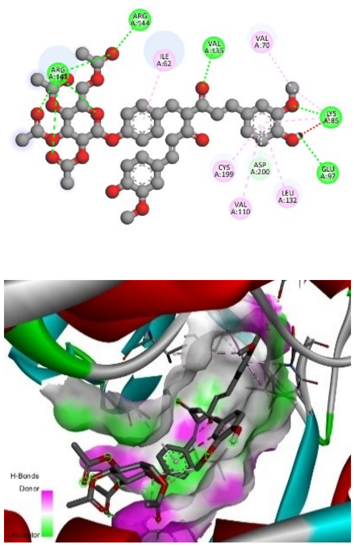

3a-Glc: –10.14 kcal/mol

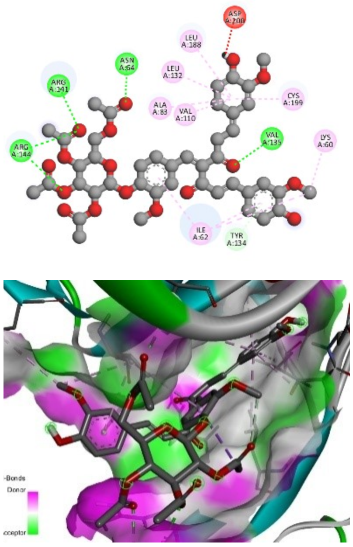

4a-Glc: –7.96 kcal/mol

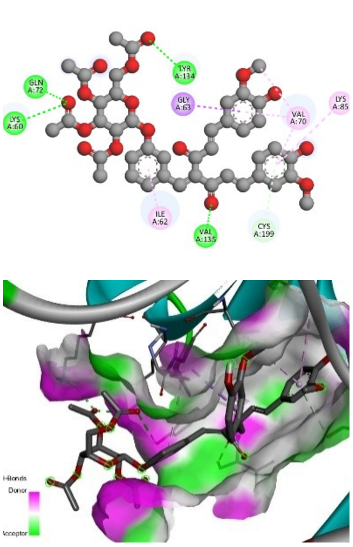

2b-Glc: –9.75 kcal/mol

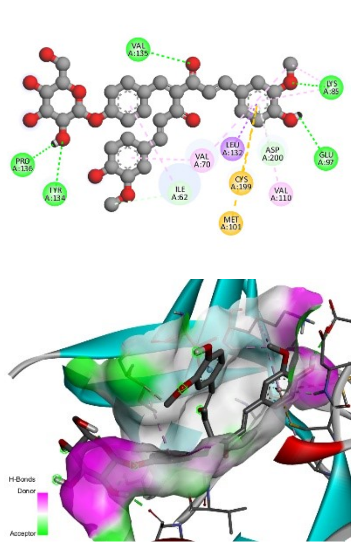

3b-Glc: –9.59 kcal/mol

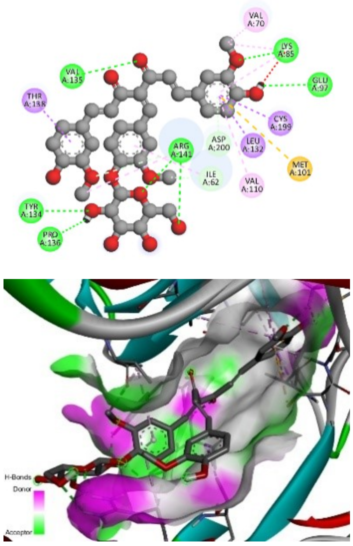

4b-Glc: –10.53 kcal/mol

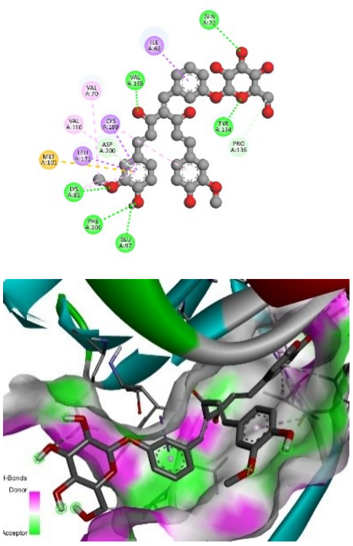

**2a-Man**: –8.12 kcal/mol 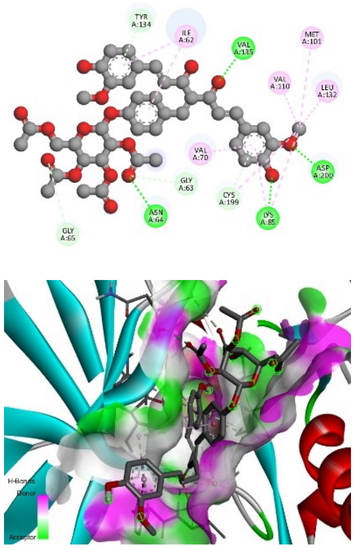	**3a-Man**: –9.38 kcal/mol 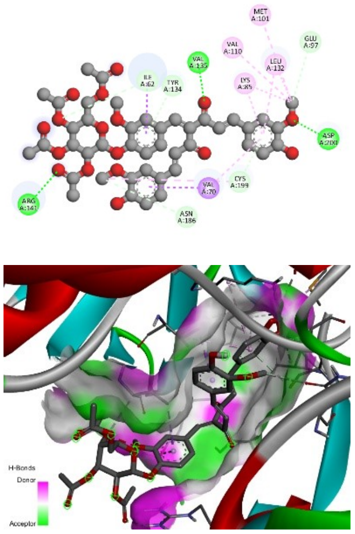	**4a-Man**: –10.14 kcal/mol 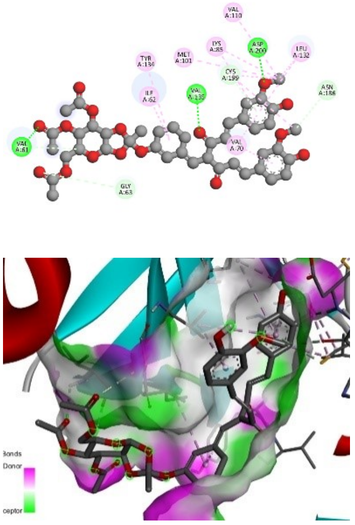
**2b-Man**: –10.35 kcal/mol 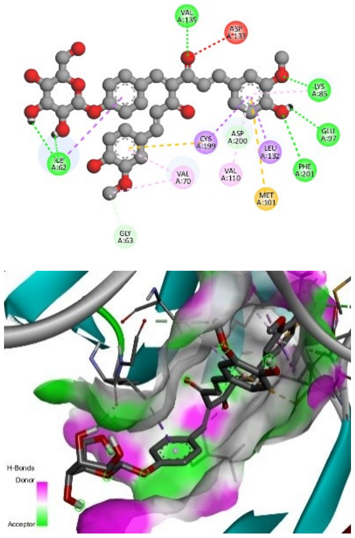	**3b-Man**: –8.02 kcal/mol 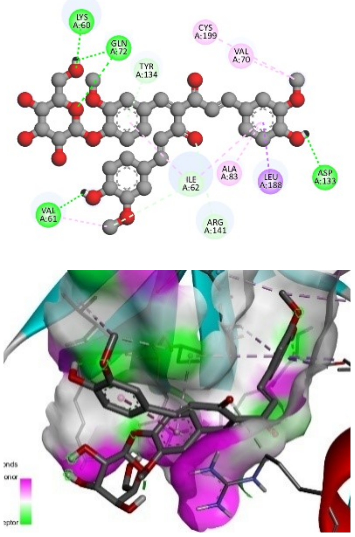	**4b-Man**: –9.53 kcal/mol 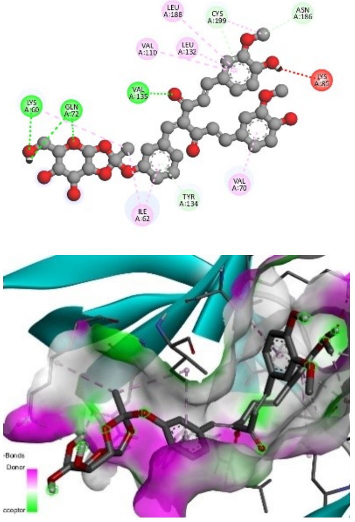

6. DFT geometry optimisation analysis

6.1 Calculation details for global reactivity analysis

Calculation details of the electronic and global reactivity parameters from DFT calculations [1, 2]

1. Ionisation potential (IP) is defined as $-E_{HOMO}$

2. Electron affinity (EA) is defined as $-E_{LUMO}$

3. Chemical potential (μ) is defined as $-\frac{1}{2}(IP+EA)$. This represents the energy change of the system which can be defined as the ability of the system to gain electrons. It is correlated with the HOMO and LUMO energy gap, in which the smaller gaps indicate higher chemical reactivity. According to Koopmans' theorem, $IP=-E_{HOMO}$ and $EA=-E_{LUMO}$. Therefore, $\mu=\frac{1}{2}(E_{HOMO}+E_{LUMO})$

4. Chemical hardness (η), proposed by Parr and Pearson [3] is defined as $\eta=\frac{1}{2}(IP-EA)$ and $\eta=-\frac{1}{2}(E_{HOMO}-E_{LUMO})$

5. Electronegativity (χ) is defined as $\chi=\frac{1}{2}(IP+EA)$ and $\chi=-\frac{1}{2}(E_{HOMO}+E_{LUMO})$

6. Electrophilicity index (ω) is defined as $\frac{\mu^2}{2\eta}$. This determines the electrophilicity (ability to accept electrons) and nucleophilicity (ability to donate electrons) of a chemical system, which is defined by Parr et al. [3]

6.2 Visualisation of HOMO-LUMO band gap

Frontier molecular orbitals (FMOs) which illustrate the HOMO-LUMO energy gap of the glycoside derivatives compounds

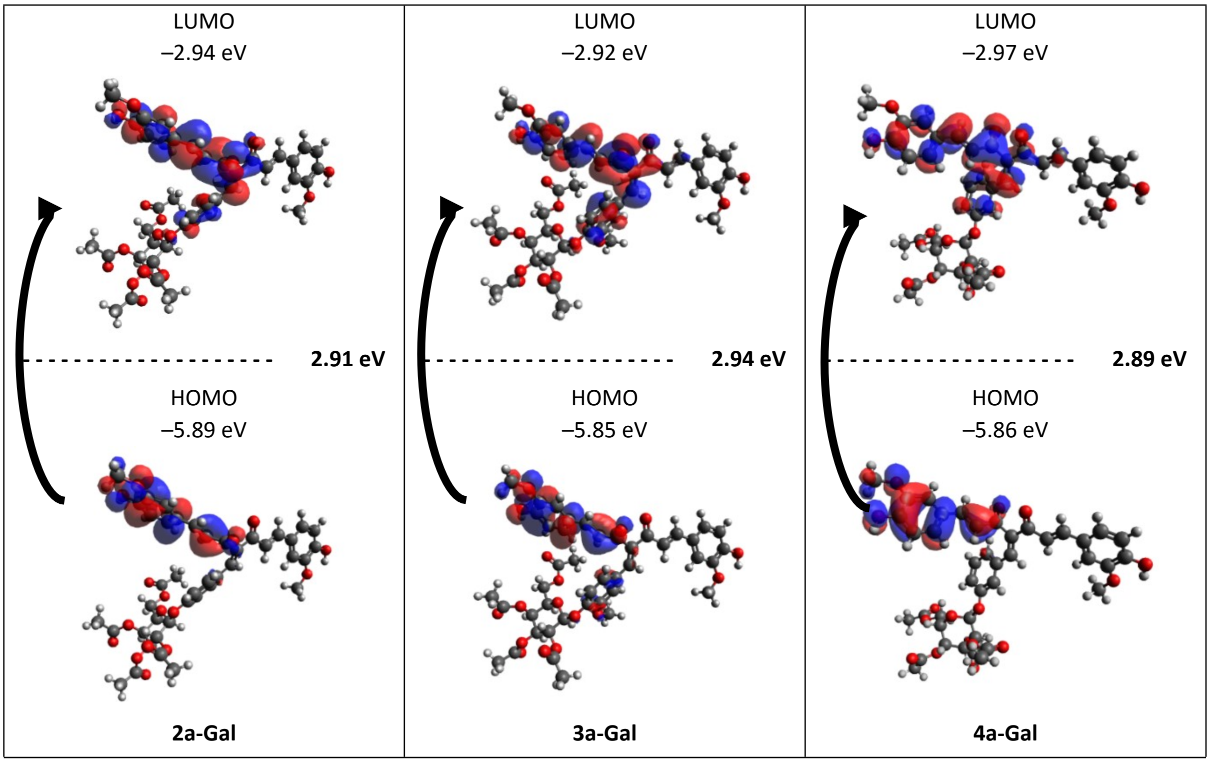

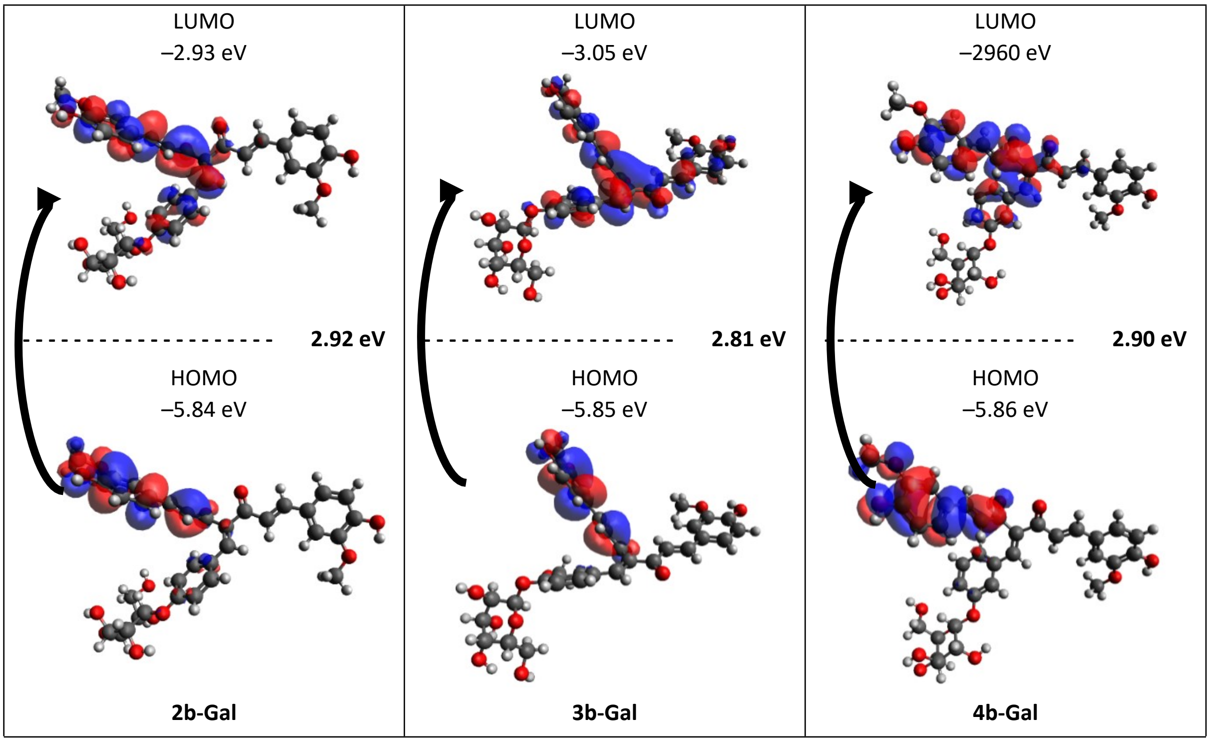

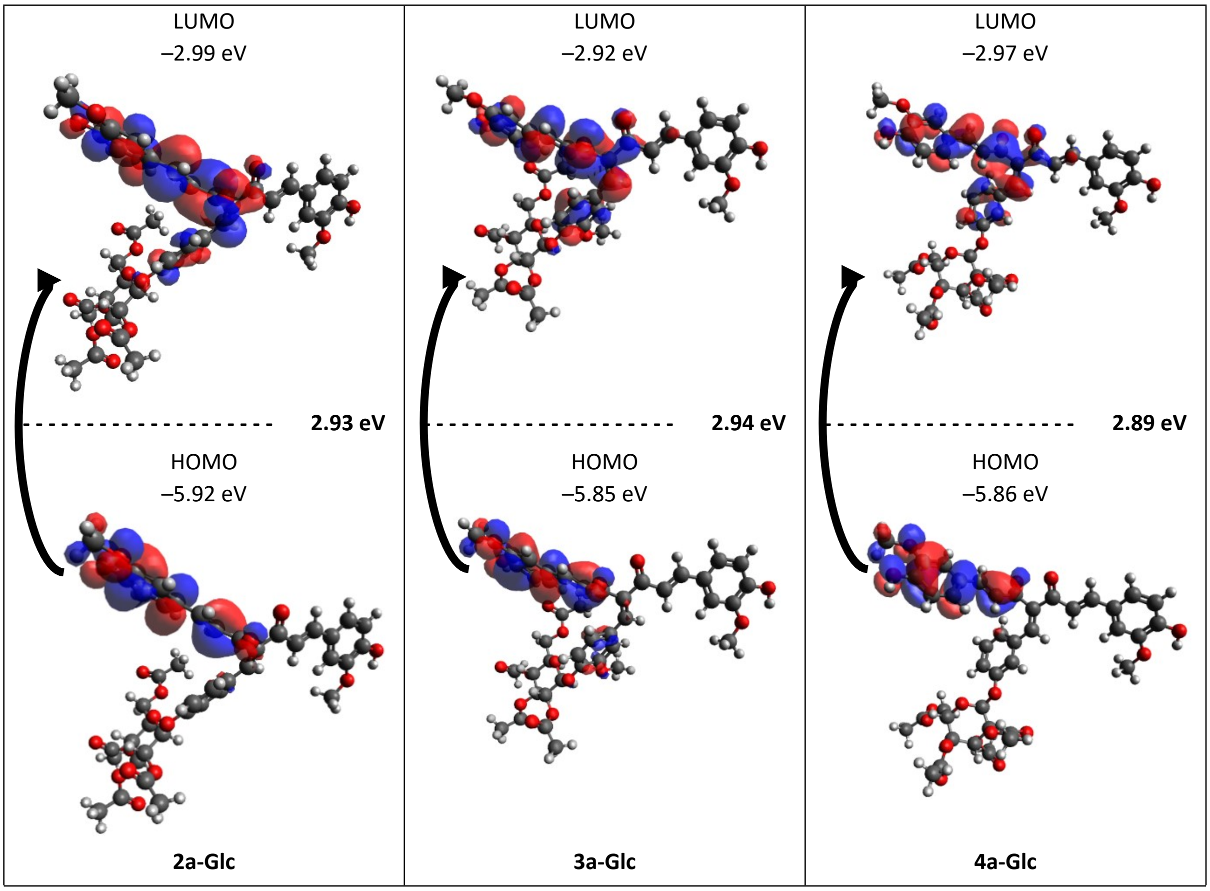
LUMO
−2.99 eV
2.93 eV
HOMO
−5.92 eV
2a-Glc
LUMO
−2.92 eV
2.94 eV
HOMO
−5.85 eV
3a-Glc
LUMO
−2.97 eV
2.89 eV
HOMO
−5.86 eV
4a-Glc

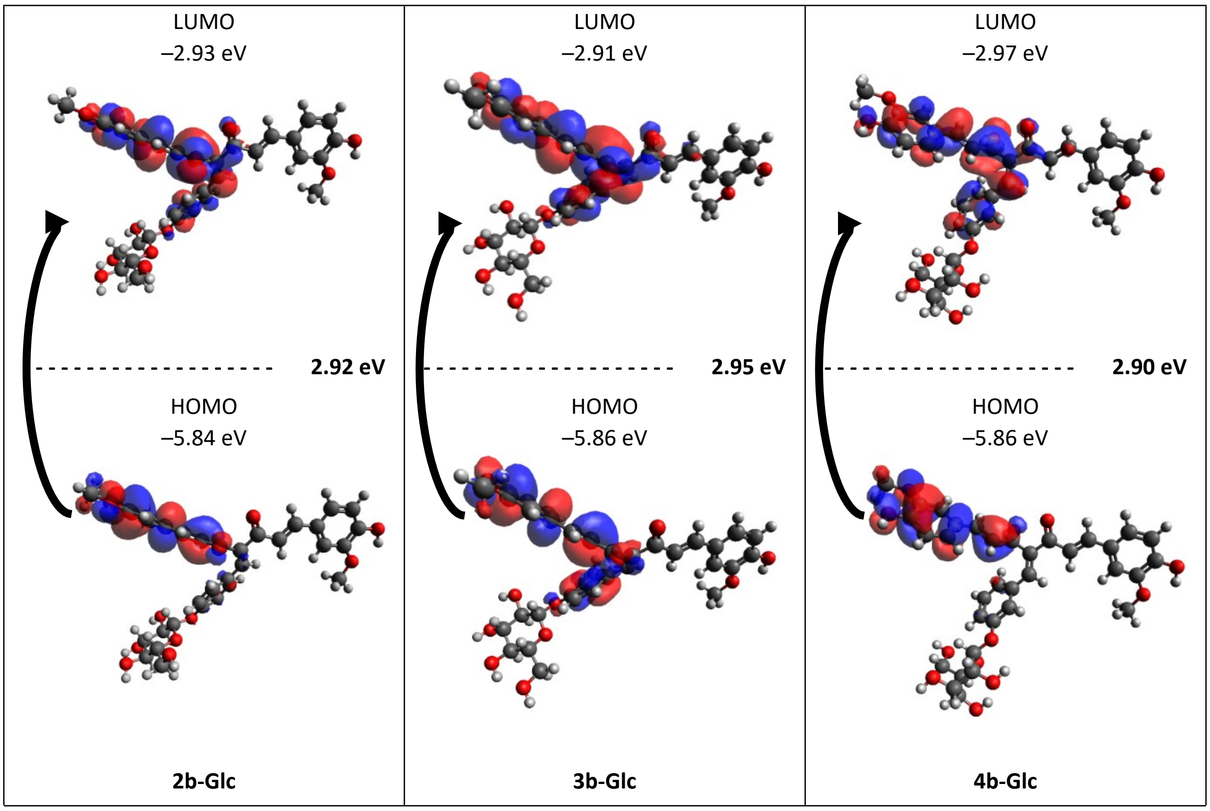
LUMO
−2.93 eV
2.92 eV
HOMO
−5.84 eV
2b-Glc
LUMO
−2.91 eV
2.95 eV
HOMO
−5.86 eV
3b-Glc
LUMO
−2.97 eV
2.90 eV
HOMO
−5.86 eV
4b-Glc

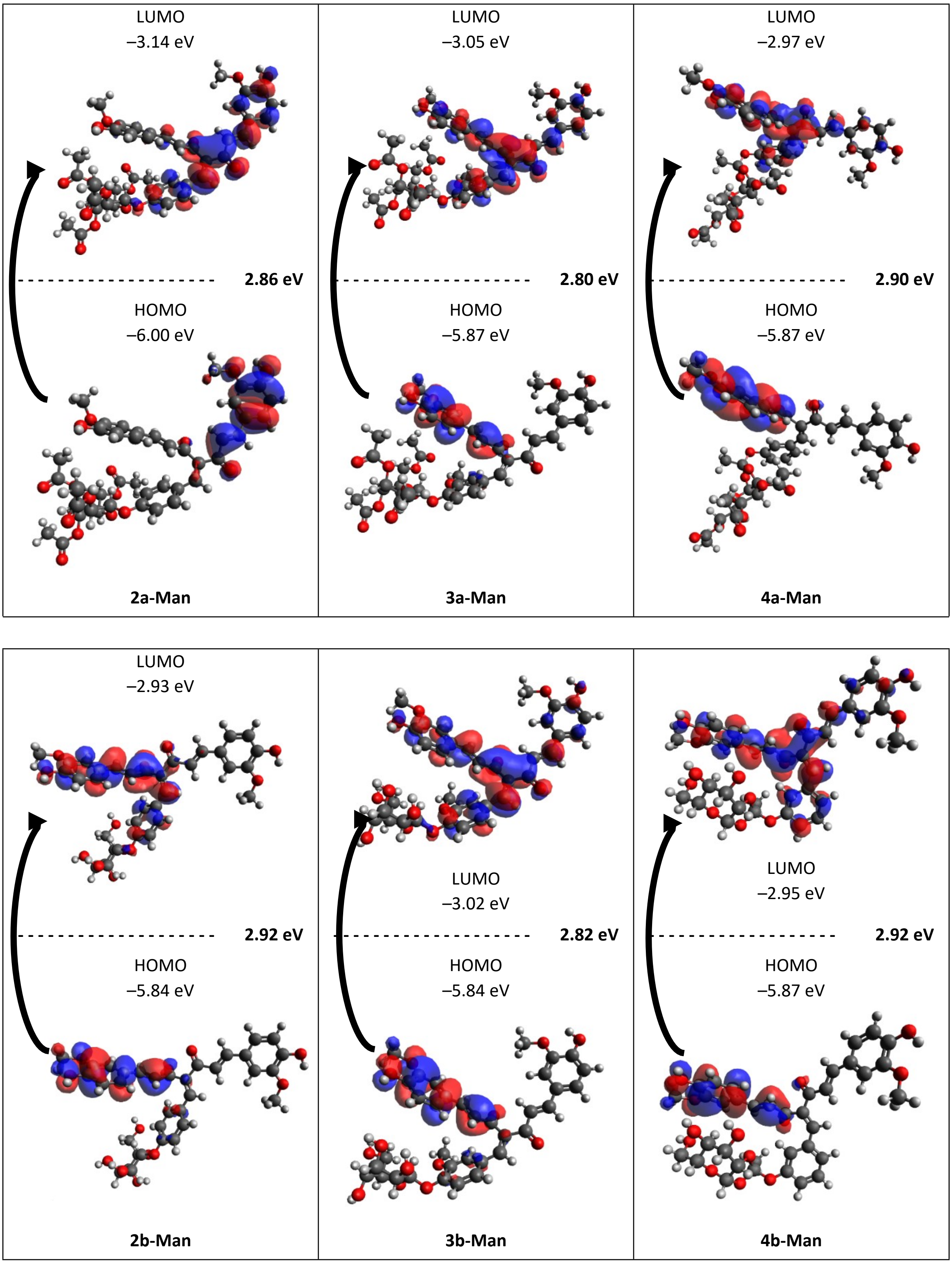
LUMO
–3.14 eV
2.86 eV
HOMO
–6.00 eV
2a-Man
LUMO
–3.05 eV
2.80 eV
HOMO
–5.87 eV
3a-Man
LUMO
–2.97 eV
2.90 eV
HOMO
–5.87 eV
4a-Man
LUMO
–2.93 eV
2.92 eV
HOMO
–5.84 eV
2b-Man
LUMO
–3.02 eV
2.82 eV
HOMO
–5.84 eV
3b-Man
LUMO
–2.95 eV
2.92 eV
HOMO
–5.87 eV
4b-Man

7. Haemin binding potential

Isothermal titration calorimetry profiles of the interaction between HMN with glycoside compounds, acquired in a 10 mM sodium phosphate buffer (pH 7.4) at 37 °C

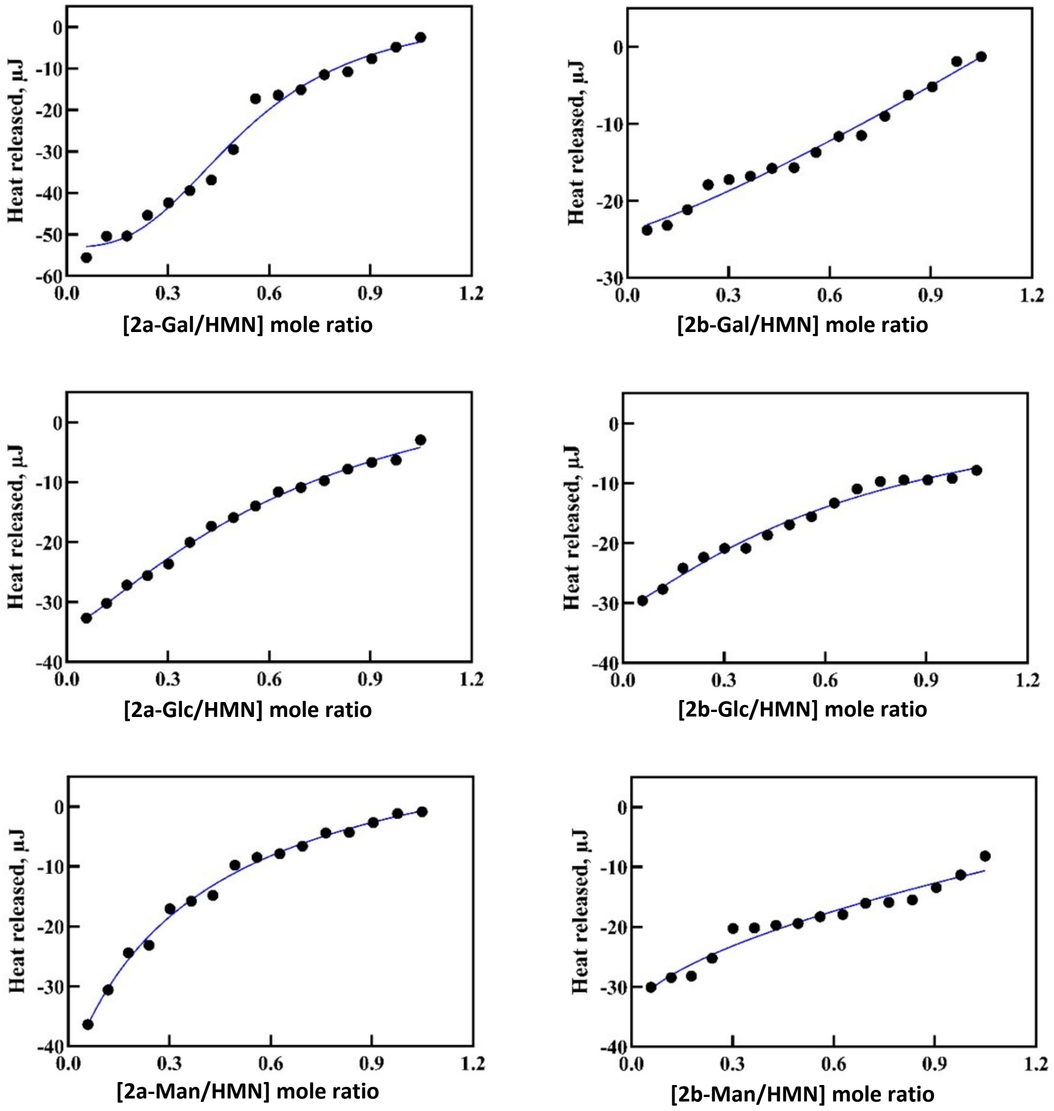

8. References

[1] R. Hazarika and B. Kalita, Elucidating the therapeutic activity of selective curcumin analogues: DFT-based reactivity analysis. *Struct. Chem*. 2021, 32, 1701-1715.

[2] A. N. Malik, A. Ali, M. Ashfaq, M. N. Tahir, M. M. Alam, M. S. Mostafa and A. Kuznetsov, Asynthetic approachtowardsdrugmodification:2 hydroxy-1-naphthaldehyde based imine-zwitterion preparation, single-crystal study, Hirshfeld surface analysis, and computational investigation. *RSC Adv*. 2024, 14, 6476-6493.

[3] R. G. Parr and R. G. Pearson Absolute hardness: companion parameter to absolute electronegativity. *J. Am. Chem. Soc*. 1983, 105, 7512-7516.
